# A 3.3 Å‐Resolution Structure of Hyperthermophilic Respiratory Complex III Reveals the Mechanism of Its Thermal Stability

**DOI:** 10.1002/anie.201911554

**Published:** 2019-11-28

**Authors:** Guoliang Zhu, Hui Zeng, Shuangbo Zhang, Jana Juli, Xiaoyun Pang, Jan Hoffmann, Yan Zhang, Nina Morgner, Yun Zhu, Guohong Peng, Hartmut Michel, Fei Sun

**Affiliations:** ^1^ National Laboratory of Biomacromolecules Institute of Biophysics (IBP) Chinese Academy of Sciences 15 Datun Road, Chaoyang District Beijing 100101 China; ^2^ Department of Molecular Membrane Biology Max Planck Institute of Biophysics Max-von Laue-Strasse 3 60438 Frankfurt am Main Germany; ^3^ Institute of Physical and Theoretical Chemistry Goethe University Max-von Laue-Strasse 7 60438 Frankfurt am Main Germany; ^4^ University of Chinese Academy of Sciences Beijing 100101 China

**Keywords:** cytochrome bc_1_ complex, enzyme catalysis, hyperthermophilic species, protein structures, protein–protein interactions

## Abstract

Respiratory chain complexes convert energy by coupling electron flow to transmembrane proton translocation. Owing to a lack of atomic structures of cytochrome *bc*_1_ complex (Complex III) from thermophilic bacteria, little is known about the adaptations of this macromolecular machine to hyperthermophilic environments. In this study, we purified the cytochrome *bc_1_* complex of *Aquifex aeolicus*, one of the most extreme thermophilic bacteria known, and determined its structure with and without an inhibitor at 3.3 Å resolution. Several residues unique for thermophilic bacteria were detected that provide additional stabilization for the structure. An extra transmembrane helix at the N‐terminus of cyt. *c*_1_ was found to greatly enhance the interaction between cyt. *b* and cyt. *c*_1_, and to bind a phospholipid molecule to stabilize the complex in the membrane. These results provide the structural basis for the hyperstability of the cytochrome *bc_1_* complex in an extreme thermal environment.

## Graphical abstract

**A hot take on thermophiles**: The hyperstability of respiratory complex III from *Aquifex aeolicus* provides a suitable environment for the internal electron transfer reaction at high temperature.

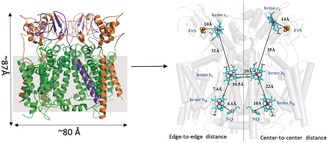

## Introduction

Cellular respiration complexes convert redox energy into a transmembrane electrochemical proton gradient, which is used to synthesize adenosine triphosphate (ATP) or to transport various substances. In this process, the cytochrome *bc*_1_ complex (also known as complex III) plays a key role by catalyzing the electron transfer from quinols to cytochrome *c* simultaneously transporting protons across the membrane according to the “Q‐cycle” mechanism.^1^ At present, various structures of cytochrome *bc*_1_ complexes from vertebrates,^2^ yeast,^3^ and α‐proteobacteria^4^ are available. They have provided many details that have helped to understand the structural arrangement and the catalytic mechanism of these protein assemblies. The subunit composition of the complex varies between species,2d, 3a, 4d but three conserved core subunits are always present, namely cytochrome *b* (cyt. *b*) with the cofactor heme *b*_L_ and heme *b*_H_, cytochrome *c*_1_ (cyt. *c*_1_) with cofactor heme *c*_1_, and a Rieske iron‐sulfur protein (ISP) with a binuclear iron sulfur cluster (2Fe‐2S).2a, 5 Each cytochrome *bc*_1_ complex contains two quinol/quinone binding sites, the oxidation site Q_o_ and the reduction site Q_i_, which are the targets for natural and designed inhibitors.1d, 6 All cytochrome *bc*_1_ complexes are nearly symmetric dimers. Our knowledge of cytochrome *bc*_1_ complex structures has been so far limited to those from mesophilic species, so little information is available on its structures from thermophiles, which hinders our understanding of its unique thermal stability allowing maintenance of the electron transfer reaction under extreme conditions.

*Aquifex aeolicus* is a hyperthermophilic chemoautotrophic *ϵ*‐proteobacterium with adaptive growth temperatures in the range of 85–95 °C.^7^ As one of the most hyperthermophilic bacteria known, *A. aeolicus* is thought to be one of the oldest bacterial species. It is distributed in hydrothermal environments on land and in oceans throughout the world, including hot compost piles or deep gold mines. *A. aeolicus* is recognized as the representative organism not only of the *Aquifex* genus but also of the *Aquificaceae* family and the order *Aquificales*.^8^ In order to live at extremely high temperature, the proteins, nucleic acids, lipids, and other biomolecules of the organism must be adapted. This feature makes of *A. aeolicus* an ideal organism to study the structure and function of thermophilic proteins.

*A. aeolicus* is a chemolithotrophic hydrogen oxidizer, so its respiratory chain complexes usually use hydrogen as the primary electron donor and oxygen as electron acceptor in order to provide energy for metabolism. In particular, its cytochrome *bc*_1_ complex uses a naphthoquinone derivative, 2‐VI,VII‐tetrahydromultiprenyl‐1,4‐naphthoquinone (NQ) (23), as special substrate for electron transfer, with NQ being reduced to NQH_2_ by electrons from hydrogen oxidation in previous reactions.^9^ Moreover, this complex has a significantly increased stability at extremely high temperatures. In the present study, using single‐particle electron cryomicroscopy (cryo‐EM), we determined the structure of the cytochrome *bc*_1_ complex from *A. aeolicus* without and with an inhibitor at 3.3 Å resolution. These structures not only reveal the precise arrangement of the *bc*_1_ core subunits but also provide information about the causes for its thermostability and suggest possible mechanisms by which electrons are transferred in extreme thermal environments.

## Results

### Structure Determination and Overall Structure of Cytochrome *bc*_1_ Complex

**Figure 1:**
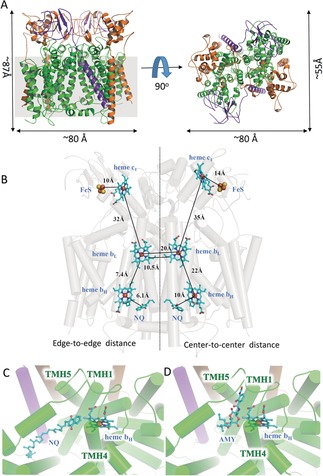
Overall structure of the cytochrome *bc*_1_ complex from *Aquifex aeolicus*.^17^ A) The protein structure (ribbon model) of the cytochrome *bc*_1_ complex is shown in cartoon representation in two different views. The subunits cyt. *b*, cyt. *c*_1_ and ISP are colored in green, orange and purple, respectively. The scales are indicated besides the model, and the grey square represents the cell membrane. B) Location of the cofactors and the substrates (1,4‐naphthoquinone, NQ) of the cytochrome *bc*_1_ complex is shown using cyan stick representation, while the proteins are transparent. The edge‐to‐edge and center‐to‐center distances between cofactors are provided. C) View into the Q_i_ binding site with NQ in the native structure. D) View into the Q_i_ binding site with antimycin A (AMY) in the inhibitory structure.

The cytochrome bc_1_ complex from *A. aeolicus* was solubilized with dodecyl β‐d‐maltoside from membranes. The sample contains a mixture of complex III and complex IV as identified by laser‐induced liquid bead ion desorption (LILBID) MS (Supporting Information, Figure S1).^10^ *A. aeolicus* complex III contains all three core catalytic subunits: cyt. *b* with heme *b*_L_ and heme *b*_H_, cyt. *c*_1_ with heme *c*_1_ and the ISP with the binuclear Fe–S cluster (2Fe‐2S).^11^ The structure of complex III was determined after high‐resolution refinement with 93 622 particles. The overall structure of the dimeric cytochrome *bc*_1_ complex reaches a resolution of 3.3 Å according to the gold standard FSC_0.143_ (Fourier Shell Correlation) criterion (Figures S2 A–E and S3). It is the first structure of a respiratory chain complex from *A. aeolicus*, as well as the first of a 1,4‐naphthoquinol oxidizing cytochrome *bc*_1_ complex. It has dimensions of ca. 87 Å in height and 80 Å in length (Figure 1 A). In this map, models for the three core subunits of cyt. *b*, cyt. *c*_1_ and ISP, together with their cofactors (hemes *b*_H_, *b*_L_, *c*_1_, 2Fe‐2S cluster) and substrates (a 1,4‐naphthoquinone), could be built (Figure 1 B and Figure S4). All three subunits form a *C*_2_‐symmetric dimeric structure through the interaction of a cyt. b dimer.

### 1,4‐Naphthoquinones Are Involved in the “Q‐Cycle”

In the classic Q‐cycle mechanism, a complete enzymatic reaction of the cytochrome *bc*_1_ complex involves two cycles, in which two ubiquinols (UQH_2_) are oxidized in the Q_o_ site. The first UQH_2_ delivers one electron to cytochrome *c*_1_ through the ISP, whereas the second electron is transferred to a UQ in the Q_i_ site leading to the formation of a semiquinone radical. The two protons from UQH_2_ are released to the external space. This process has to be repeated in order to generate a stable doubly reduced and protonated UQ thus yielding a UQH_2_ in the Q_i_ site, which is released immediately and can be used as a substrate in the Q_o_ site.^12^ The cytochrome *bc*_1_ complex from *A. aeolicus* has been shown to use a 1,4‐naphthoquinone (NQ), as a special substrate, to mediate electron transfer.9a In our cryo‐EM structure, several NQ molecules can be clearly traced in both the Q_i_ site and on the cytoplasmic side, but none are around the Q_o_ site. In particular, a well‐defined density map for a NQ is clearly visible in the Q_i_ pocket formed by TMH1, TMH4 and TMH5 of the cyt. *b* (Figure 1 C). This observation makes good sense because NQ is not a substrate, but a product at the Q_o_ site. Its tight binding might lead to product inhibition. The edge‐to‐edge distance between heme *b*_H_ and NQ in the Q_i_ site is 6.1 Å, this short distance ensures efficient electron transfer from heme *b*_H_ to NQ.

We also determined the cryo‐EM structure of the cytochrome *bc_1_* complex from *A. aeolicus* with the Q_i_ site inhibitor antimycin A (AMY). Antimycin A was found to block the electron transfer from the high spin heme *b*_H_ to the quinone or semiquinone. The entire structure of the inhibited enzyme complex is identical with the native structure, except for the Q_i_ site (Figure 1 D). In the native state, a 1,4‐naphthoquinone molecule is located near heme *b*_H_, but in the structure with the inhibitor, it is replaced by AMY, suggesting that this inhibitor competitively occupies the Q_i_ site and prevents the entry of the substrate NQ, thus blocking the electron transport and the entire respiratory chain reactions.

### The Three Protein Subunits of the Cytochrome *bc*_1_ Complex from *A. aeolicus*

**Figure 2:**
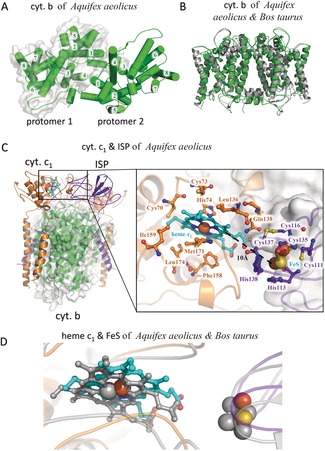
Cyt. *b*, cyt. *c*_1_, and ISP of *Aquifex aeolicus*. A) The dimers formed by two cyt. *b* subunits are shown in green as a cartoon representation, indicating eight TMHs (1–8) and two protomers (with or without white surface). B) The structure superposition of the cyt. *b* subunits of *Aquifex aeolicus* (green) and *Bos taurus* (gray). C) The cyt. *c*_1_ (orange) and ISP (blue‐purple) subunits of *Aquifex aeolicus* shown as cartoon representations, indicating cofactors and important residues. D) Structure superposition of cyt. c_1_ and ISP subunits of *Aquifex aeolicus* (coloured) and *Bos taurus* (grey).

The cyt. *b* subunit of *A. aeolicus* has 409 amino acid residues forming 13 helices, including eight transmembrane helices (TMHs), which interact in the membrane with two TMHs of cyt. *c*_1_ and one TMH of the ISP (Figure 1 A). The two cyt. *b* protomers bind to each other mainly through TMH1 and TMH4 to form a stable dimer (Figure 2 A). Its overall conformation is basically the same as that of other cyt. *b* subunits, with RMSD values around 2 Å (Figure 2 B). The subunit cyt. *c*_1_ of *A. aeolicus* contains 5 helices and one heme *c*_1_ as cofactor, which is held by the conserved CXXCH motif with the residues Cys70, Cys73, and His74 (Figure 2 C). Several conserved hydrophobic residues, like Ile159, Met171, Leu136 and Phe158, also interact with heme *c*_1_. Importantly, the N‐terminal TMH1 of cyt. *c*_1_ forms a unique structure that will be discussed later.

The third subunit, the ISP, contains one TMH, and one functional domain containing the [2Fe‐2S] cluster, and an interconnecting linker region. Around the 2Fe‐2S cluster a series of highly conserved coordinating residues can be identified including His138, His113, Cys111, Cys135, Cys116, and Cys137 which bind and stabilize the 2Fe‐2S cluster (Figure 2 C). In our structure, the 2Fe‐2S cluster containing domain is found near cytochrome *c*_1_, the edge‐to‐edge distance between heme *c*_1_ and the 2Fe‐2S cluster is only 10 Å. This result suggests that the electron transfer between these two cofactors of *A. aeolicus* can be rapid and efficient. Their relative positions and orientations are the same as in the corresponding subunits of Bos *taurus* (Figure 2 D).

### The Sequence Characteristics of the Cytochrome *bc*_1_ Complex of *A. aeolicus*

**Figure 3:**
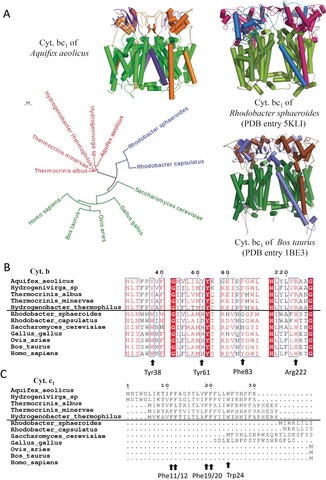
The sequence characteristics of cytochrome bc_1_ complex from *Aquifex aeolicus*. A) The evolutionary tree of the three core subunits of the cytochrome bc_1_ complex from different species. Three representative complex structures are shown as cartoons, while the subunits cyt. *b*, cyt. *c*_1_, and ISP are colored differently. B) Sequence alignment of cyt. *b* subunit from different species, indicating important residues. C) Sequence alignment of the cyt. *c*_1_ subunits from different species, indicating important residues.

The overall structure of the cytochrome *bc*_1_ complex of *A. aeolicus* is similar to other reported cytochrome *bc*_1_ complexes, such as those from *Rhodobacter sphaeroides* (PDB entry: 5KLI) and *B. taurus* (PDB entry: 1BE3) (Figure 3 A). However, the hyperthermophilic life of *A. aeolicus* may require adaptive changes of the amino acid sequences and structural characteristics of its important protein complexes. In order to study the adaptations of the cytochrome *bc*_1_ complex to hyperthermophilic life, the amino acid sequences of the cytochrome *bc*_1_ complex from different species, including thermophilic bacteria (*A. aeolicus, Hydrogenivirga sp, Thermocrini salbus, Thermocrinis minervae, and Hydrogenobacter thermophilus*), other prokaryotes (*R. sphaeroides and Rhodobacter capsulatus*) and eukaryotes (*Saccharomyces cerevisiae, Gallus gallus, Ovis aries, B. taurus and Homo sapiens*) were aligned and an evolutionary tree was constructed. The results show that all three protein subunits of the cytochrome *bc*_1_ complexes show obvious sequence differences between the three groups of species (Figure 3 A). The amino acid sequences of the cytochrome *bc*_1_ complex protein subunits of all five thermophilic bacteria are closely related, suggesting that the same or similar changes of the amino acid sequences occurred during the adaptation to their thermophilic environment. Alternatively, the thermophilic cytochrome *bc*_1_ complexes might have been acquired by horizontal gene transfer. The high sequence similarity of the TMH1s of cyt. *c*_1_ argues for this latter possibility.

According to the sequence alignment result, we have identified several unique residues that are present in the thermophilic bacteria but different in mesophilic species. In the cyt. *b* subunit, the amino acid residues specific for the thermophilic species include Tyr38, Tyr61, Phe83, and Arg222 (Figure 3 B). In the cyt. *c*_1_ subunit, the whole N‐terminal TMH1 spanning 1–30 amino acids, is totally missing in all mesophilic species (Figure 3 C). There are seven phenylalanine (Phe11–12, 19–21, 25, 29) and two tryptophan residues (Trp4, 24) located in this helix, forming a unique “WF‐rich motif”. Based on the cryo‐EM structure of the cytochrome *bc*_1_ complex from *A. aeolicus*, we were able to analyze the important functions of these unique residues in thermophilic bacteria.

### Heme *b*_H_ and 1,4‐Naphthoquinone Binding are Further Stabilized in *A. aeolicus*

**Figure 4:**
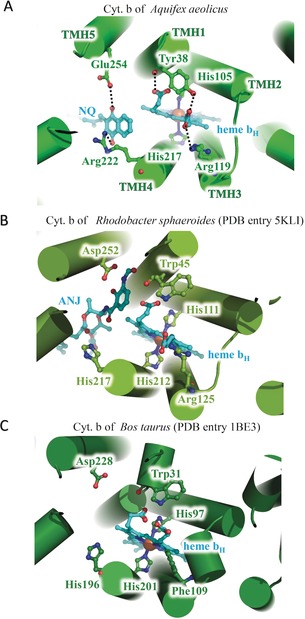
Structure comparison of the heme *b*_H_ sites in the cyt. *b* subunits of A) *Aquifex aeolicus*, B) *Rhodobacter sphaeroides and* C) *Bos taurus*. The proteins are shown as cartoon representations, while important residues and ligands are indicated and shown as stick models.

In the cyt. *b* subunit of the cytochrome *bc*_1_ complex from *A. aeolicus*, heme *b*_H_ interacts with residues of TMH1, TMH2, and TMH4. The heme–Fe coordinating residues are identified as the highly conserved His105 and His217 for heme *b*_H_ (Figure 4 A). These interactions and the spatial conformation of heme *b*_H_ are the same in all the cyt. *b* proteins from different species, and serve to protect the electron transfer reaction at the Q_i_ site (Figure 4 B,C). However, in the structure of the cytochrome *bc*_1_ complex from *A. aeolicus*, the carboxyl groups of heme *b*_H_ additionally interact with Tyr38 and Arg119 (Figure 4 A) leading to a stabilization of heme *b*_H_ binding. It is worth noting that the carbonyl oxygen and phenyl‐hydroxy group of Tyr38 bind to both carboxyl groups of heme *b*_H_. The tyrosine residue is highly conserved in the thermophilic bacteria (Figure 3 B) but replaced by a tryptophan residue in the mesophilic species. In the structure of the cytochrome *bc*_1_ complex of *R. sphaeroides* (PDB entry 5KLI) or *B. taurus* (PDB entry 1BE3), Trp45 or Trp31 interact with only one of the two carboxyl groups of heme *b*_H_. Thus, the additional interaction we observe probably stabilizes heme *b*_H_ binding at high temperatures.

In our structure, there are two 1,4‐naphthoquinone molecules in each Q_i_ site (Figure 7 A), one of them being located close to the heme *b*_H_ molecule with an edge‐to‐edge distance of 6.1 Å, allowing fast electron transfer. The plane of the NQ head‐group is almost perpendicular to the porphyrin plane of heme *b*_H_. Interestingly, the binding of this active NQ is stabilized by interactions with Glu254 of TMH5 and Arg222 from TMH4 of cyt. *b*. Importantly, Arg222 residue is replaced by a histidine in *R. sphaeroides* and *B. taurus* (Figure 3 B). Compared with the short histidine residue, the positively charged side chain of Arg222 is closer to the oxygen atom of NQ. Thus, in the proteins from the hyperthermophilic species, Arg222 could stabilize the binding of the substrate NQ at the center Q_i_ site, to orient NQ favourably and optimize the distance to heme *b*_H_.

### More Stable TMHs and Increased Affinity with the Q‐pool in *A. aeolicus*

**Figure 5:**
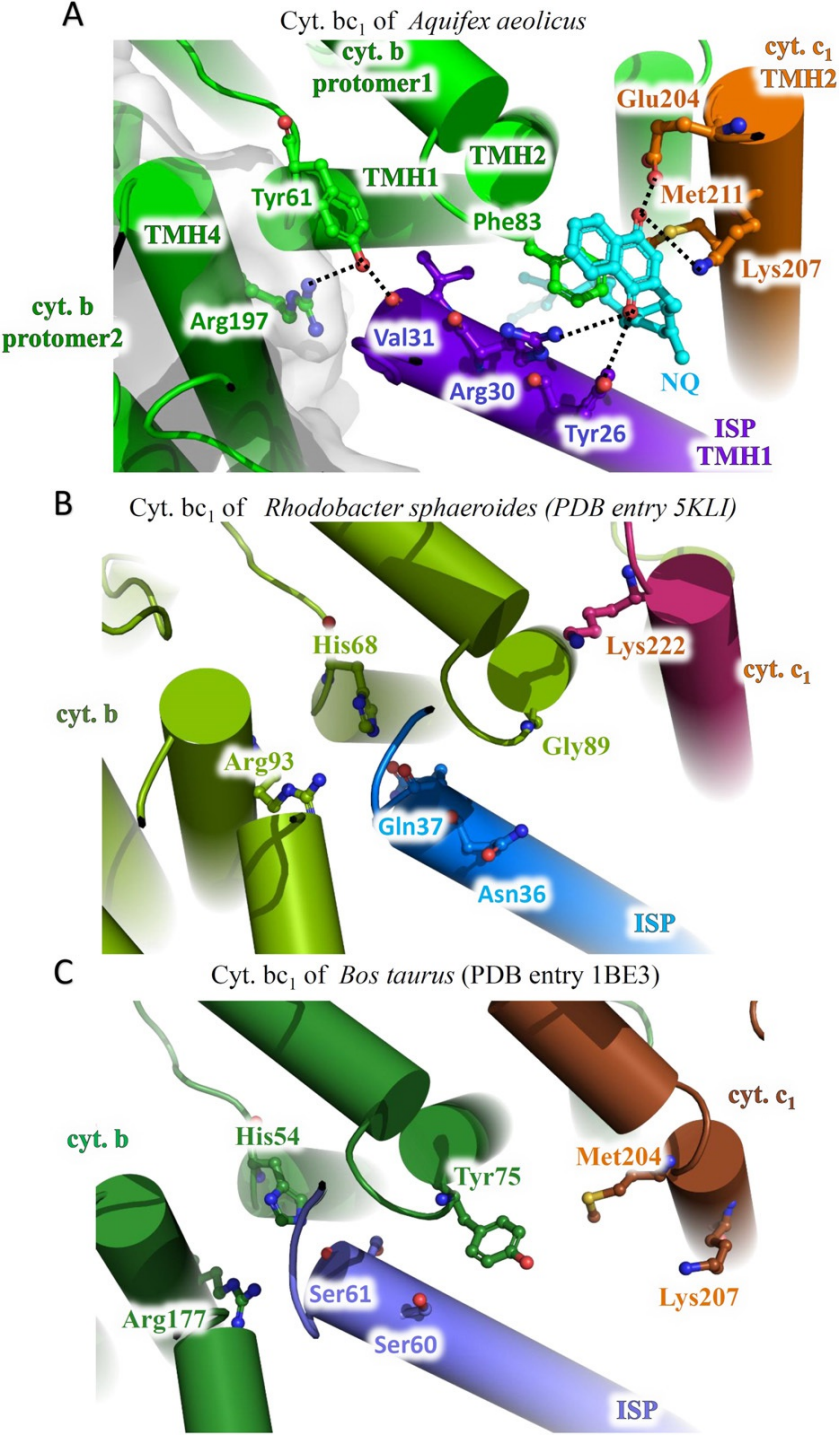
Structure comparison around TMH1 of ISP subunits of A) *Aquifex aeolicus*, B) *Rhodobacter sphaeroides and* C) *Bos taurus*. The proteins are shown as cartoon representations, while important residues and ligands are indicated and shown as sticks.

In the dimeric structure of the cytochrome *bc*_1_ complex of *A. aeolicus*, two cyt. *b* protomers bind to each other, mainly through their TMH regions. We find that a residue unique to the thermophiles, Tyr61 of cyt. *b*, is involved in this dimer interaction. The phenyl‐hydroxy of Tyr61 in TMH1 of one protomer binds to the nitrogen atom of Arg197 in TMH4 of another protomer enhancing the interactions between the two cyt. *b* protomers (Figure 5 A). Moreover, Tyr61 is also close to the carbonyl oxygen of Val31 in TMH1 of the ISP subunit, which enhances the interactions between these two subunits in the complex. In the structure of cytochrome *bc*_1_ complex from *R. sphaeroides* and *B. taurus*, this tyrosine residue is replaced by a histidine residue (His68 or His54), which cannot interact with the adjacent arginine residue in TMH4 of the other protomer (Figure 5 B,C). Tyr61 is highly conserved in all thermophilic bacteria (Figure 3 B). Consequently, Tyr61 of cyt. *b* helps to stabilize the TMH region in the complex.

Interestingly, in our complex structure, there is a 1,4‐naphthoquinone molecule buried in a hydrophobic pocket formed by TMH2 of cyt. *b*, TMH1 of the ISP and TMH2 of cyt. *c*_1_ (Figure 5 A). This pocket is located away from both the Q_i_ and Q_o_ site but close to the phospholipid layer around the complex (Figure 7 B). This NQ molecule remained tightly bound to the cytochrome *bc*_1_ complex of *A. aeolicus* throughout the protein purification process, and likely originates from the Q‐pool in the phospholipid layer. This type of quinone binding has not been found in other cytochrome *bc*_1_ structures (Figure 5 B,C). In the cytochrome *bc*_1_ complex of *A. aeolicus* Glu204 and Lys207 of TMH2 of the cyt. *c*_1_ subunit bind to one carbonyl oxygen of NQ, while Arg30 and Tyr26 from TMH1 of the ISP subunit bind to the other carbonyl oxygen of NQ. The Phe83 on TMH2 of the cyt. *b* subunit and Met211 of the cyt. *c*_1_ subunit interact with the benzene ring of NQ. Among these residues, Phe83 of cyt. *b,* a highly conserved residue in the thermophilic bacteria (Figure 3 B). In the complex structures of *R. sphaeroides* or *B. taurus*, this phenylalanine residue is replaced by a glycine or a tyrosine residue, respectively, and the internal chemical environment of this pocket has also changed a lot. Therefore, no quinone molecules have been found inside these pockets in their structures. This observation suggests that Phe83 of cyt. *b* enhances the binding of NQ to the cytochrome *bc*_1_ complex.

### TMH1 of cyt. *c*_1_ Improves the Stability of the Complex in the Membrane

**Figure 6:**
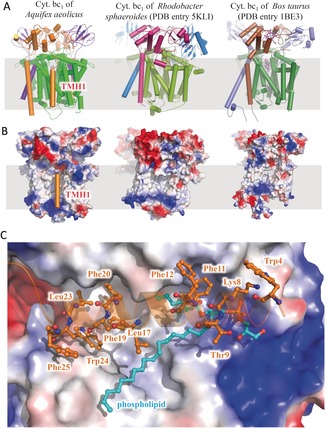
The extra N‐terminal TMH (TMH1) of the cyt. *c*_1_ subunits from *Aquifex aeolicus*. A) The cytochrome *bc*_1_ complex from *Aquifex aeolicus*, *Rhodobacter sphaeroides*, and *Bos taurus* are shown as cartoon representations, indicating the location of the extra TMH of cyt. *c*_1_ from *Aquifex aeolicus*. B) The cytochrome *bc*_1_ complex from three species is shown as electrostatic surface except for the TMH1 of cyt. *c*_1_. C) Interaction details between TMH1 of cyt. *c*_1_ with the complex and the phospholipid ligand.

The cyt. *c*_1_ subunit of *A. aeolicus* possesses two TMHs, an N‐terminal one and a C‐terminal one. On the contrary, in all other known cytochrome *bc*_1_ structures, the cyt. *c*_1_ subunit possesses only the C‐terminally located TMH (Figure 6 A). The sequence alignment shows that, this N‐terminal TMH1 of the cyt. *c*_1_ subunit of *A. aeolicus* contains a phenylalanine/tryptophan rich (WF‐rich) motif, which is found in all investigated thermophilic bacteria but is missing in the mesophilic species (Figure 3 C). In our structure, this WF‐rich TMH1 binds to the hydrophobic surface of the TMH region of the complex in the phospholipid layer (Figure 6 B). Importantly, this kind of interaction leads to the formation of a deep hydrophobic groove, which traps a phospholipid molecule. The hydrophilic head group binds to the positively charged region of the complex through its phosphate moiety, while its long hydrophobic tail is buried by a series of hydrophobic residues on TMH1 of cyt. *c*_1_ subunit, including Phe11, Leu17, Phe19, and Phe20. Other hydrophobic residues of TMH1 bind to the hydrophobic surface of cyt. *b* to stabilize this pocket, including Tyr4, Phe12, Leu23, Tyr24 and Phe25 (Figure 6 C). By calculating the surface area of the protein complex, we found that the addition of this TMH1 motif increases the interface between cyt. *b* and cyt. *c*_1_ subunits by 60 %, from 1560 Å^2^ to 2494 Å^2^. Therefore, the unique N‐terminal TMH1 of cyt. *c*_1_ not only enhances the subunit interactions inside the cytochrome bc_1_ complex but also sequesters a phospholipid molecule inside the complex. This feature may lead to an additional stabilization of the complex in the membrane.

## Discussion

The structural and functional studies of respiratory complexes have continued for many years.2e, 3d, 13 However, there has been a lack of atomic structures of the complexes from thermophilic bacteria apart from the respiratory complex I and the cytochrome *ba*_3_ from *Thermus thermophilus*.^14^ In the present study, we purified the cytochrome *bc*_1_ complex from *A. aeolicus* and determined its structure at 3.3 Å resolution using single‐particle cryo‐EM. This structure reveals the conformations of the three core subunits, namely cyt. *b*, cyt. *c*_1_ and the ISP, as well as the mode of binding of the cofactors and of the 1,4‐naphthoquinone substrate (Figure 1 A). The relative locations and distances among hemes, the 2Fe‐2S center and 1,4‐naphthoquinones support the existence of a Q cycle reaction mechanism in the respiratory chain of *A. aeolicus* (Figure 1 B). Using this structure, we have identified several sequences and structural characteristics for thermophilic bacteria, which are not found in other structures and could protect the structure and catalytic activity of the complex at extremely high temperature.

**Figure 7:**
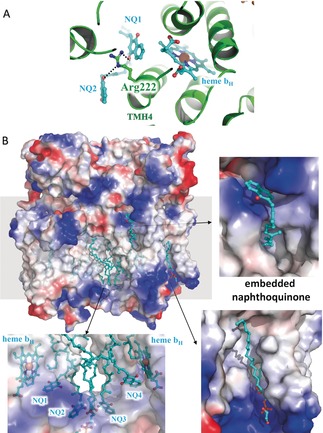
The potential 1,4‐naphthoquinone channel around the Q_i_ site of cyt. *b* of *Aquifex aeolicus*. A) Two NQ molecules are located in the Q_i_ site of cyt. *b* subunit of *Aquifex aeolicus*. The cytochrome *bc*_1_ complex from *Aquifex aeolicus* is shown as cartoon representation, while Arg222 and the ligands are indicated and shown as stick models. B) The potential 1,4‐naphthoquinone channel around the Q_i_ site, with the embedded naphthoquinone and the phospholipid. The cytochrome bc_1_ complex from *Aquifex aeolicus* is shown as electrostatic surface, while the ligands are indicated and shown as stick models.

On one hand, the cytochrome *bc*_1_ complex from *A. aeolicus* has enhanced overall stability in the membrane. Tyr61 of cyt. *b* interacts with both the ISP and another cyt. *b* protomer to form a tighter complex (Figure 5 A). A NQ substrate from the Q‐pool is trapped inside a hydrophobic pocket and binds to a series of hydrophobic and hydrophilic residues in the three subunits of the cytochrome *bc*_1_ complex (Figure 5 A). Moreover, there is an extra TMH at the N‐terminus of cyt. *c*_1_, which strongly binds to cyt. *b* and traps a phospholipid molecule inside the complex. Therefore, the cytochrome *bc*_1_ complex from *A. aeolicus* is able to grab a 1,4‐naphthoquinone from the Q‐pool and to sequester a phospholipid in the membrane (Figure 7 B), thus forming a more stable conformation at high temperature and providing a suitable environment for the internal electron transfer reaction.

On the other hand, the electron transport pathway within the cytochrome *bc*_1_ complex from *A. aeolicus* appears to be stabilized by enhanced binding of the prosthetic groups. In the cyt. *b* subunit of *A. aeolicus*, Tyr38 and Arg119 bind to the two carboxyl groups of the cofactor heme *b*_H_, then Glu254 and Arg222 bind to the two carbonyl groups of the 1,4‐naphthoquinone substrate. Thus, both cofactor and substrate in the Q_i_ reaction site are more stabilized in the cytochrome *bc*_1_ complex of *A. aeolicus*, compared to the complexes from other species. At high temperatures, the thermal motions of molecules are enhanced, so their relative positions and distances change quickly, which might reduce the electron transfer efficiency between them.

In a previous study, the crystal structure of cytochrome *c*_555_ from *A. aeolicus* was determined at 1.15 Å resolution.^15^ Interestingly, there is also a unique 14‐residue long extra helix in this structure, which strongly binds to the core structure of *c*_555_. This helix motif is demonstrated to contribute to the hyperstability of the *c*_555_, and to help *A. aeolicus* to adapt to the hyperthermophilic environment. In our structure, the N‐terminal extra helix TMH1 of cyt. *c*_1_ is much longer than that of *c*_555_, and the various hydrophobic residues of TMH1 increase the interface between the cyt. *b* and cyt. *c*_1_ subunits by 60 % (Figure 6). This additional interaction could also contribute to the hyperstability of the overall complex. More importantly, TMH1 of cyt. *c*_1_ fixes a phospholipid molecule in a unique hydrophobic groove. This kind of conformation has not been found in other mesophilic species, which could help *A. aeolicus* to adapt to the hyperthermophilic environment.

In the structure, we found density for two 1,4‐naphthoquinone substrates in the Q_i_ site of cyt. *b*, around Arg222 residues on TMH4 (Figure 7 A). The tail of the 1,4‐naphthoquinone stretches towards the membrane core outside of the binding pocket without interacting with cyt. *b*. However, the tail of the ubiquinone identified in previous structures stretches towards heme *b*_H_.3a In addition, the entire antimycin A inhibitor was found in the Q_i_ pocket and shows a strong interaction with Glu254. There are five hydrogen bonds seen between antimycin A and cyt. *b*. In contrast, previously only the head group of this inhibitor was reported to be bound to the Q_i_ site.1d, 2c, 16 It is possible that this location represents the NQ channel from the Q‐pool, in which the substrates are transferred into or out of the catalytic center (Figure 7 B). The unique Arg222 residue helps to stabilize the substrate binding and may help the substrates to reach their binding site close to heme *b*_H_ in the hyperthermophilic environment. Data collection and structure determination details are summarized in Table 1.

**Table 1:** Statistics of data collection, image processing, and model building.

Sample	native cytochrome bc_1_ complex	Inhibited cytochrome bc_1_ complex
Data collection		
Microscope	FEI Titan Krios	
Voltage [kV]	300	
Detector	Gatan Bioquantum K2	
Energy filter	20 eV	
Pixel size [Å/pixel)]	1.04	
Electron dose [e^−^ Å^−2^]	60	
Defocus range [μm]	−1.5 to −2.5	
Reconstruction		
Software	RELION 3.0‐beta/ RELION 2.0	
Number of used particles	93 622	81 350
Accuracy of rotation	1.748	
Accuracy of translations (pixel)	0.697	
Symmetry	C2	
Map sharpening B‐factor [Å^2^]	−140	−56
Final resolution [Å]	3.28	3.22
Model building		
Software	Coot	
Model refinement		
Software	PHENIX	
Map CC (whole unit cell)	0.76	0.77
Map CC (around atoms)	0.73	0.74
Rmsd (bonds) [Å]	0.008	0.009
Rmsd (angle) [°]	1.181	1.249
Model composition		
Protein residues	1530	
Heme groups	6	
[2Fe‐2S] clusters	2	
Validation		
Ramachandran plot		
Outliers [%]	0.26	0.40
Allowed [%]	9.67	8.74
Favored [%]	90.07	90.86
Rotamer outliers [%]	0.77	1.23

## Conclusion

In summary, we solved the 3.3 Å structure of respiratory complex III from the hyperthermophilic chemoautotrophic ϵ‐proteobacterium *Aquifex aeolicus* and revealed the structural basis for the hyperstability of proteins in an extreme thermal environment. It is the first 1,4‐naphthoquinone structure in the Q_i_ sites. Several residues unique for thermophilic bacteria were detected that provide additional stabilization for ligand binding and for the structure of the whole complex. It was able to grab 1,4‐naphthoquinones and to sequester phospholipids in the membrane, thus forming a more stable conformation at high temperature and providing a suitable environment for the internal electron transfer reaction. These results provide structural basis for the hyperstability of the cytochrome *bc_1_* complex in an extreme thermal environment.

## Conflict of interest

The authors declare no conflict of interest.

## Supporting information

As a service to our authors and readers, this journal provides supporting information supplied by the authors. Such materials are peer reviewed and may be re‐organized for online delivery, but are not copy‐edited or typeset. Technical support issues arising from supporting information (other than missing files) should be addressed to the authors.

Supplementary

Supplementary
