# Supplementary material for: A 3.3 Å‐Resolution Structure of Hyperthermophilic Respiratory Complex III Reveals the Mechanism of Its Thermal Stability
Source: Angew Chem Int Ed Engl. 2019 Nov 28;59(1):343–51. doi: 10.1002/anie.201911554 (PMC7004027; doi:10.1002/anie.201911554)
Supplement: Supplementary file 1 — Supplementary [file ANIE-59-343-s001.pdf]

Angewandte Chemie
Eine Zeitschrift der Gesellschaft Deutscher Chemiker

Supporting Information

A 3.3 Å-Resolution Structure of Hyperthermophilic Respiratory Complex III Reveals the Mechanism of its Thermal Stability

Guoliang Zhu+, Hui Zeng+, Shuangbo Zhang+, Jana Juli, Xiaoyun Pang, Jan Hoffmann, Yan Zhang, Nina Morgner, Yun Zhu, Guohong Peng,* Hartmut Michel,* and Fei Sun**

anie_201911554_sm_miscellaneous_information.pdf
anie_201911554_sm_Movie_S1.mp4

Author Contributions

F.S., H.M. and G.P. initiated and supervised the project. G.Z., H.Z., S.Z., X.P., J.J., J.H., Y.Z. and G.P. performed all the experiments. G.Z. and S.Z. performed image processing and solved the cryo-EM structure. H.Z., G.Z., J.H., N.M., G.P, and Y.Z. analyzed the data and wrote the paper with substantial input from H.M. and F.S.

Table of Contents

Experimental Procedures .... S 2
Fig. S1. Identification of the cytochrome bc_1 complex by LILBID-MS .... S 4
Fig. S2. Structure determination of the cytochrome bc_1 complex from *A. aeolicus*. .... S 5
Fig. S3. Local resolution map of the final 3D density map. .... S 6
Fig. S4. Representative cryo-EM densities of the proteins and ligands of cytochrome bc_1 complex from *Aquifex aeolicus*. .... S 7
Movie. S1 (separate file). The overall map with model fitted .... S 8
References .... S 8
Author Contributions .... S 8
Dataset. S1. Full wwPDB/EMDataBank EM Map/Model Validation Report of native cytochrome bc1 complex .... S 9
Dataset. S2. Full wwPDB/EMDataBank EM Map/Model Validation Report of inhibited cytochrome bc_1 complex S55

Experimental Procedures

Purification and identification of the cytochrome *bc*$_1$ complex

The cytochrome *bc*$_1$ complex was purified directly from the cells of the hyperthermophilic and microaerobic bacterium *A. aeolicus*. As a consequence of the limited availability of cells and lack of multiple methods for expression level optimization, many efforts have been focused on purification optimization according to previously reported methods [1]. The result of detergent screening for solubilization suggested dodecyl-β-D maltoside (DDM) has the best extraction efficiency for the cytochrome *bc*$_1$ complex. Then the DDM-solubilized membrane proteins were separated by ion exchange chromatography (Mono Q 10/100 GL, GE). Then fractions containing the cytochrome *bc*$_1$ complex were pooled and subjected to size exclusion chromatography. The complex was purified by a first size exclusion chromatography step (TSK4000SW, Tosoh) in 20 mM Tris-HCl pH 7.4, 150 mM NaCl and 0.05 % (w/v) DDM. Considering that large micelles of DDM are indistinguishable from protein particles, the purified cytochrome *bc*$_1$ was applied to a second size exclusion chromatography column (Yarra SEC-4000 column, Phenomenex) to exchange the detergent from DDM to digitonin. The major peak fractions were pooled, and the protein concentration was determined by the BCA assay (Pierce) according to the manufacturer's protocol. After degassing and ultrafiltration, the purified cytochrome *bc*$_1$ complex (20 µg) was subjected to laser-induced liquid-bead ion-desorption mass spectrometry (LILBID-MS) [2] to measure the masses of individual subunits and the intact complex. The mass analysis was done using a time of flight mass spectrometer with a Wiley-McLaren-type acceleration region and an ion reflector. Three independent experiments were performed to ensure the accuracy of the results. The inhibitor complex was prepared by adding antimycin A to the purified proteins with 10 times of molar ratio.

Cryo-EM Data Acquisition and Processing

An initial model of the cytochrome *bc*$_1$ complex was obtained after negative stain electron microscopy. Briefly, 5 µl of the purified complex sample (0.01 mg/ml) were applied for one minute to glow-discharged copper grids coated with a thin carbon film. After washing 5 times with ultrapure water and uranyl acetate (2% w/v), the sample was stained by uranyl acetate for one minute. Then images were collected on a FEI Talos microscope operated at 200 kV.

For cryo-EM, 3 µl of the freshly purified complex sample (1.5 mg/ml) were applied to glow-discharged holey carbon film grids (GIG Au R1/1, 300 mesh), which had been treated with H_2 and O_2 mixtures in a Gatan Solarus plasma cleaner for 1 min. Grids were blotted at 16 °C and 100% humidity for 3s, then flash frozen in liquid ethane and stored in liquid nitrogen using a Vitrobot (FEI). High resolution images were collected on a 300kV FEI Titan Krios transmission electron microscope using SerialEM software [3]. A Gatan K2 detector was used in super-resolution counting mode at a calibrated magnification of 130,000 × (yielding a pixel size of 0.52 Å). A quantum energy filter (Gatan, 107 Pleasanton, CA), whose energy slit was set to 20 eV, was applied to remove any inelastic scattering. Defocus values varied from -1.5 to -2.5 µm. 32 frames per stack were collected with a total exposure time of 7.4s. The dose rate was set to ~9 e^-/pixel/s and the total dose was ~60 e^-/ $Å^2$. Frame alignment, exposure weighting, and contrast transfer function parameters were estimated using Center for Biological Imaging(CBI) platform's automated data collection scripts integrated with Motioncor2 [4] and CTFFIND4 [5] during movie data collection.

Image processing

Image processing steps were performed using RELION (version 2.1 and version 3.0-beta) [6] and EMAN2 [7]. A low-resolution reconstruction of the complex was built from 1208 micrographs of the negatively stained sample. Particle picking was performed using Gautomatch (http://www.mrc-lmb.cam.ac.uk/kzhang/Gautomatch/) without a template to yield 124,989 particles. Reference-free classification was performed, and 16 good-looking classes were selected for initial model building. A total of 87 classes were selected for 3D classification, and finally one class was selected as initial model for subsequent cryo-EM data processing.

For cryo-EM data processing, Gautomatch was used to pick around 21k particles from 200 good quality images without template matching. After one round reference-free 2D classification, 10 representative classes were used as reference for automatic particle picking of all micrographs by Gautomatch, yielding an initial dataset of around 379 000 particles. These were extracted using 128×128 pixel-boxes and sorted by two rounds of 2D classifications. Then 200 982 particles from good 2D classes were selected for the 3D classification, with the previously generated initial model as reference. After that, four classes were selected to yield a subset of 148323 particles, which were re-extracted with refined particle shifts from the original micrographs using a 256×256 pixel-boxes. This refinement resulted in an overall structure at a resolution of 3.8Å. After a second round of 3D classification without alignment, one best class containing 93,622 particles from 14 classes were selected for final refinement. After data post-processing, including refining each particle defocus value by CtfRefine, the overall resolution of the structure reached 3.3 Å according to the gold standard $FSC_{0.143}$ (Fourier Shell Correlation) criterion. Local resolution variations were estimated using ResMap [8].

The data for the inhibitor complex was processed in a similar way. Briefly, 108 654 particles were reduced to 107 902 after one round of 2D classification. These particles were processed following refinement and classification without alignment, and finally 81 350 particles were used. Refinement with these particles yielded a map with a resolution of 3.5 Å. Then CtfRefine and Bayesian polishing pushed the resolution to 3.2 Å.

Model building and Refinement

To build atomic models of cytochrome *bc*$_1$ complex of *A. aeolicus*, the homologous structure from the soil bacterium *Paracoccus denitrificans* (PDB entry: 2YIU)[9] was used as initial model. We were able to trace most regions and many side chains of the three

subunits in the map using COOT [10]. The ligands and phospholipids were also docked into densities and refined using COOT and Phenix [11]. All figures were created by Pymol [12] or Chimera [13].

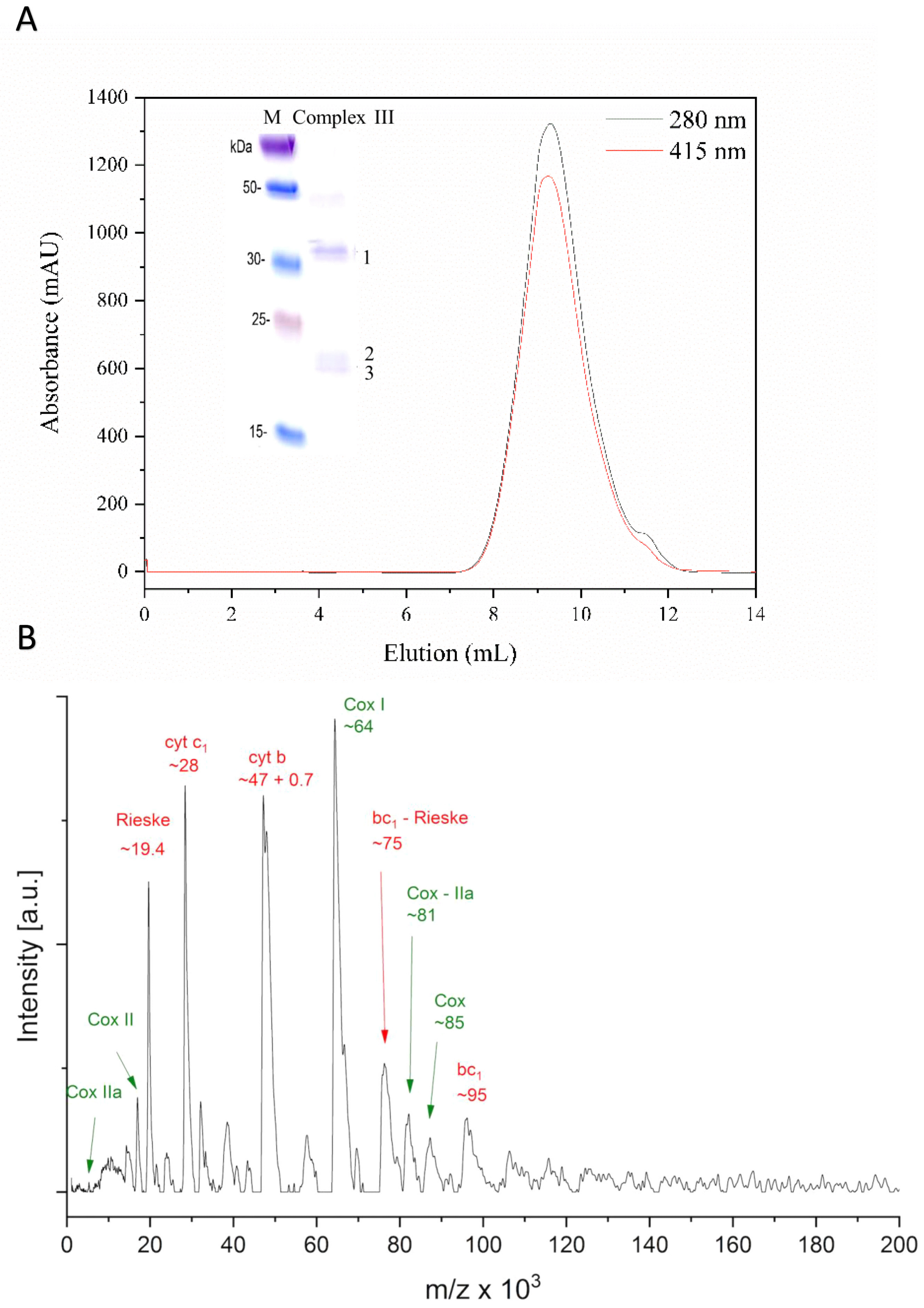

Fig. S1. Identification of the cytochrome *bc*$_1$ complex by LILBID-MS. (A) Size exclusion chromatogram and subunit compositions on SDS-PAGE. SEC elution profile was monitored the absorbance at 280 nm (black) and 415 nm (red). **(B)** Mass positions of the native complexes, subcomplexes and subunits in the sample preparation. Only a monomer *bc*$_1$ complex was identified, with a mass of 95 kDa. The sample also contains a monomer cytochrome c oxidase (Cox), the subcomplex - *bc*$_1$ complex (missing rieske protein) and Cox (missing Cox IIa) with a molecular mass of 85 kDa, 75 kDa and 81 kDa, respectively. The subunits of Cytochrome *bc*$_1$ complex are labeled as Rieske (19.4 kDa), Cyt *c*$_1$ (27.6 kDa) and Cyt *b* (46.9 kDa). The Cox subunits were identified as well, with a mass of 63.9 kDa (Cox I), 16.8 kDa (Cox II), and 5.2 kDa (Cox IIa).

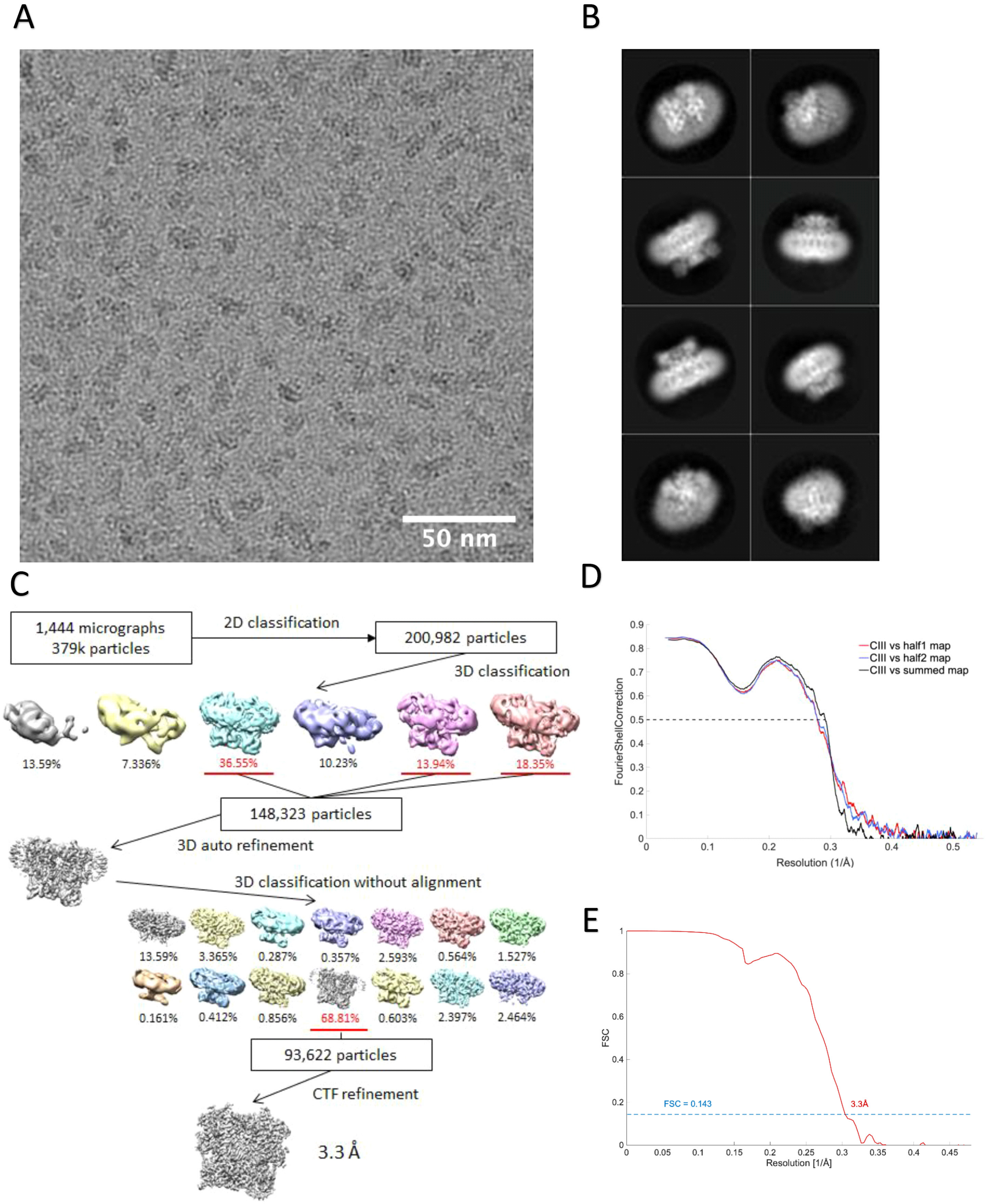

Fig. S2. Structure determination of the cytochrome *bc*$_1$ complex from *A. aeolicus*. (A) Representative micrograph of the 1,444 micrographs. **(B)** Representative 2D class averages obtained from reference-free classification. **(C)** Workflow of 3D classification and reconstruction. **(D)**The map-to-model FSC curve. **(E)** Gold-standard FSC curves for the overall maps.

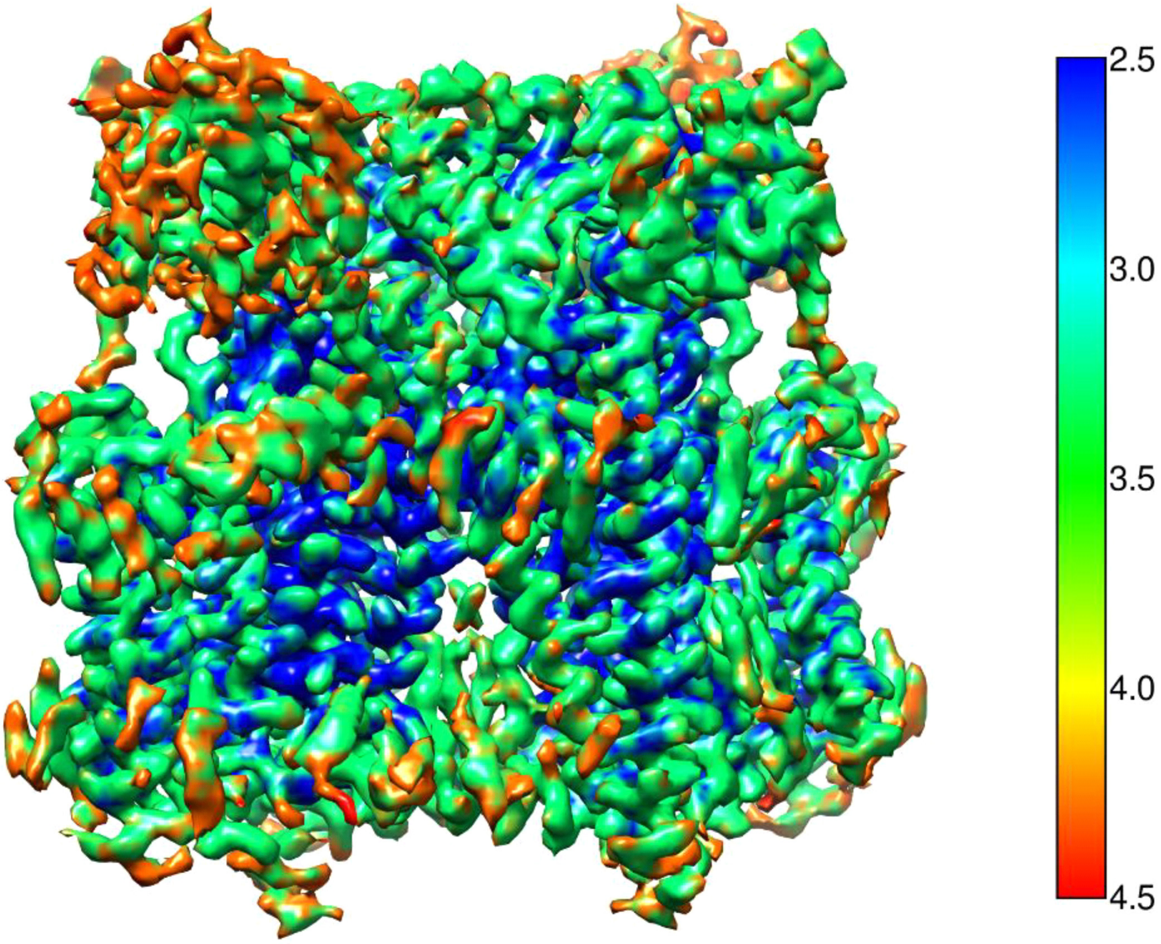

Fig. S3. Local resolution map of the final 3D density map (threshold: 0.035 in Chimera) of cytochrome bc_1 complex from *Aquifex aeolicus*. The local resolution is color-coded according to the scale bar, and the unit of number is angstrom.

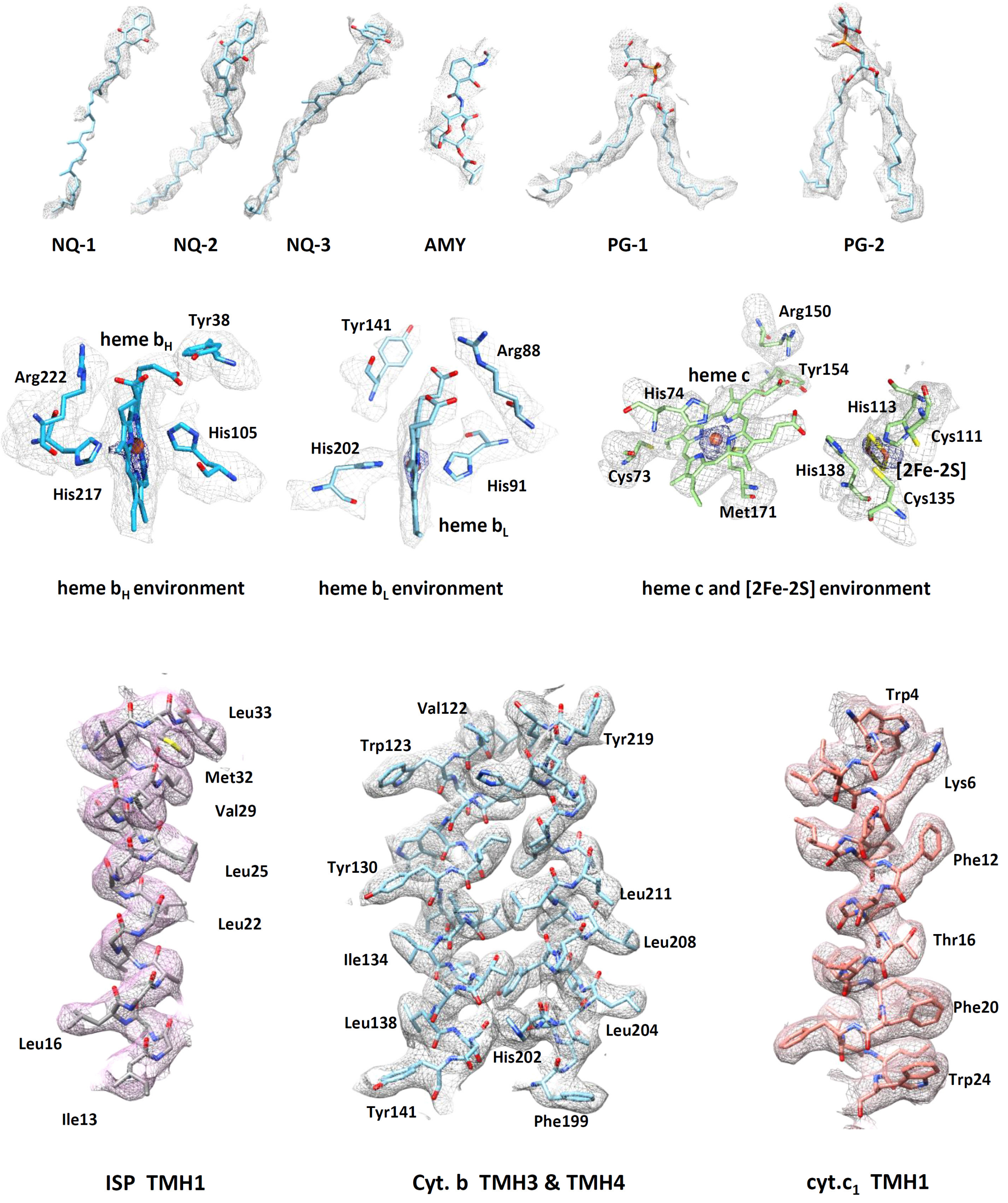

Fig. S4. Representative cryo-EM densities of the proteins and ligands of cytochrome *bc*$_1$ complex from *Aquifex aeolicus*.

Movie. S1 (separate file). The overall map with model fitted. Views are sliced to show the overall quality of the map and model refinement.

References

[1] G. H. Peng, G. Fritzsch, V. Zickermann, H. Schaagger, R. Mentele, F. Lottspeich, M. Bostina, M. Radermacher, R. Huber, K. O. Stetter, H. Michel, *Biochemistry* **2003**, *42*, 3032-3039.
[2] L. Sokolova, I. Wittig, H. D. Barth, H. Schagger, B. Brutschy, U. Brandt, *Proteomics* **2010**, *10*, 1401-1407.
[3] D. N. Mastronarde, *Journal of structural biology* **2005**, *152*, 36-51.
[4] S. Q. Zheng, E. Palovcak, J.-P. Armache, K. A. Verba, Y. Cheng, D. A. Agard, *Nature methods* **2017**, *14*, 331.
[5] A. Rohou, N. Grigorieff, *Journal of structural biology* **2015**, *192*, 216-221.
[6] aS. H. W. Scheres, *Journal of Structural Biology* **2012**, *180*, 519-530; bD. Kimanius, B. O. Forsberg, S. H. Scheres, E. Lindahl, *Elife* **2016**, *5*, e18722.
[7] G. Tang, L. Peng, P. R. Baldwin, D. S. Mann, W. Jiang, I. Rees, S. J. Ludtke, *Journal of structural biology* **2007**, *157*, 38-46.
[8] A. Kucukelbir, F. J. Sigworth, H. D. Tagare, *Nat Methods* **2014**, *11*, 63-65.
[9] T. Kleinschroth, M. Castellani, C. H. Trinh, N. Morgner, B. Brutschy, B. Ludwig, C. Hunte, *Biochim Biophys Acta* **2011**, *1807*, 1606-1615.
[10] P. Emsley, K. Cowtan, *Acta Crystallogr D Biol Crystallogr* **2004**, *60*, 2126-2132.
[11] P. D. Adams, P. V. Afonine, G. Bunkoczi, V. B. Chen, I. W. Davis, N. Echols, J. J. Headd, L. W. Hung, G. J. Kapral, R. W. Grosse-Kunstleve, A. J. McCoy, N. W. Moriarty, R. Oeffner, R. J. Read, D. C. Richardson, J. S. Richardson, T. C. Terwilliger, P. H. Zwart, *Acta Crystallogr D Biol Crystallogr* **2010**, *66*, 213-221.
[12] Schrodinger, LLC, **2015**.
[13] E. F. Pettersen, T. D. Goddard, C. C. Huang, G. S. Couch, D. M. Greenblatt, E. C. Meng, T. E. Ferrin, *J Comput Chem* **2004**, *25*, 1605-1612.

Author Contributions

F. S., H. M. and G. P. initiated and supervised the project. G. Z., H. Z., S. Z., X. P., J. J., J. H., Y. Z. and G. P. performed all the experiments. G. Z. and S. Z. performed image processing and solved the cryo-EM structure. H. Z., G. Z., J. H., N. M., G. P, and Y. Z. analyzed the data and wrote the paper with substantial input from H. M. and F. S.

Full wwPDB/EMDataBank EM Map/Model Validation Report (i)

Aug 15, 2019 – 10:31 AM JST

PDB ID	:	6KLS
EMDB ID:	:	EMD-0716
Title	:	Hyperthermophilic respiratory Complex III
Deposited on	:	2019-07-30
Resolution	:	3.30 Å(reported)

This is a Full wwPDB/EMDataBank EM Map/Model Validation Report.

This report is produced by the wwPDB biocuration pipeline after annotation of the structure.

We welcome your comments at *validation@mail.wwpdb.org*
A user guide is available at
https://www.wwpdb.org/validation/2017/EMValidationReportHelp
with specific help available everywhere you see the (i) symbol.

MolProbity	:	4.02b-467
Mogul	:	1.8.0 (224370), CSD as540be (2019)
Percentile statistics	:	20171227.v01 (using entries in the PDB archive December 27th 2017)
Ideal geometry (proteins)	:	Engh & Huber (2001)
Ideal geometry (DNA, RNA)	:	Parkinson et. al. (1996)
Validation Pipeline (wwPDB-VP)	:	2.4

CONFIDENTIAL VALIDATION REPORT

1 Overall quality at a glance (i)

The following experimental techniques were used to determine the structure:
ELECTRON MICROSCOPY

The reported resolution of this entry is 3.30 Å.

Percentile scores (ranging between 0-100) for global validation metrics of the entry are shown in the following graphic. The table shows the number of entries on which the scores are based.

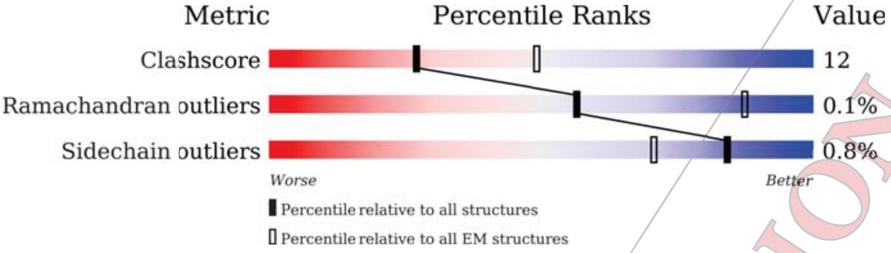

Metric	Whole archive (#Entries)	EM structures (#Entries)
Clashscore	136327	1886
Ramachandran outliers	132723	1663
Sidechain outliers	132532	1531

The table below summarises the geometric issues observed across the polymeric chains. The red, orange, yellow and green segments on the bar indicate the fraction of residues that contain outliers for >=3, 2, 1 and 0 types of geometric quality criteria. A grey segment represents the fraction of residues that are not modelled. The numeric value for each fraction is indicated below the corresponding segment, with a dot representing fractions <=5%

Mol	Chain	Length	Quality of chain
1	A	181	51% 23% • 26%
1	D	181	52% 22% • 26%
2	B	410	73% 23% •
2	E	410	73% 23% •
3	C	240	70% 28% •
3	F	240	72% 26% •

The following table lists non-polymeric compounds, carbohydrate monomers and non-standard residues in protein, DNA, RNA chains that are outliers for geometric or electron-density-fit criteria:

CONFIDENTIAL VALIDATION REPORT

Mol	Type	Chain	Res	Chirality	Geometry	Clashes	Electron density
6	DLX	B	504	-	X	-	-
6	DLX	C	301	-	X	-	-
6	DLX	E	504	-	X	-	-
6	DLX	F	301	-	X	-	-

CONFIDENTIAL VALIDATION REPORT

2 Entry composition (i)

There are 8 unique types of molecules in this entry. The entry contains 13011 atoms, of which 0 are hydrogens and 0 are deuteriums.

In the tables below, the AltConf column contains the number of residues with at least one atom in alternate conformation and the Trace column contains the number of residues modelled with at most 2 atoms.

- Molecule 1 is a protein called Rieske-I iron sulfur protein.

Mol	Chain	Residues	Atoms					AltConf	Trace
			Total	C	N	O	S		
1	A	134	1012	668	169	169	6	0	0
			Total	C	N	O	S		
1	D	134	1012	668	169	169	6	0	0

- Molecule 2 is a protein called cytochrome b subunit.

Mol	Chain	Residues	Atoms					AltConf	Trace
			Total	C	N	O	S		
2	B	395	3226	2208	491	515	12	0	0
			Total	C	N	O	S		
2	E	395	3226	2208	491	515	12	0	0

- Molecule 3 is a protein called Cytochrome c.

Mol	Chain	Residues	Atoms					AltConf	Trace
			Total	C	N	O	S		
3	C	236	1917	1263	312	334	8	0	0
			Total	C	N	O	S		
3	F	236	1917	1263	312	334	8	0	0

- Molecule 4 is FE2/S2 (INORGANIC) CLUSTER (three-letter code: FES) (formula: Fe_2S_2).

CONFIDENTIAL VALIDATION REPORT

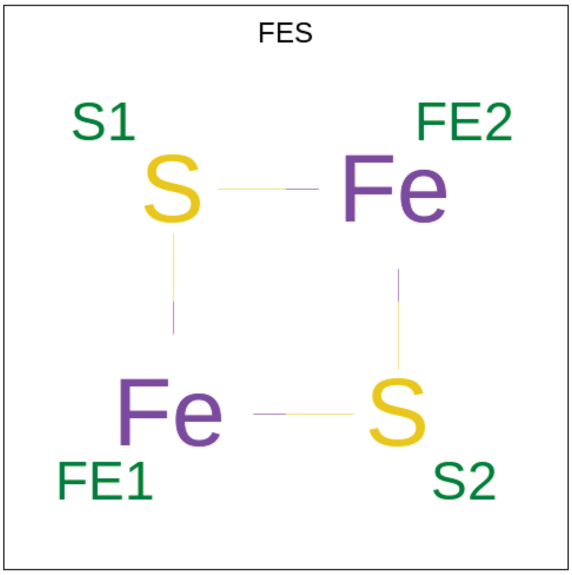

Mol	Chain	Residues	Atoms			AltConf
4	A	1	Total<br>4	Fe<br>2	S<br>2	0
4	D	1	Total<br>4	Fe<br>2	S<br>2	0

- Molecule 5 is PROTOPORPHYRIN IX CONTAINING FE (three-letter code: HEM) (formula: $C_{34}H_{32}FeN_4O_4$).

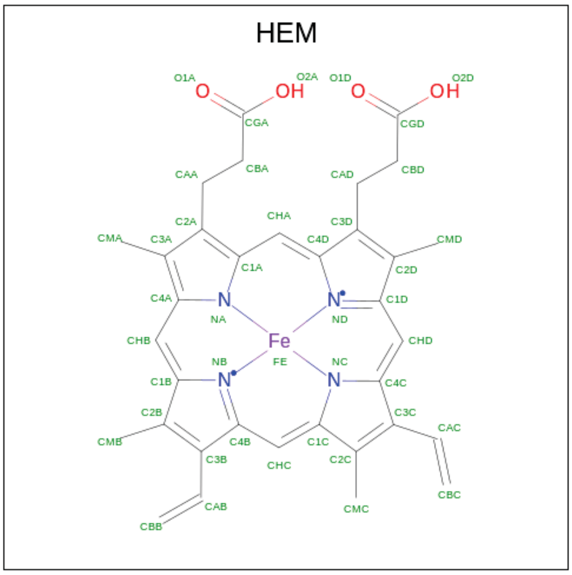

Mol	Chain	Residues	Atoms					AltConf
5	B	1	Total<br>86	C<br>68	Fe<br>2	N<br>8	O<br>8	0

Continued on next page...

CONFIDENTIAL VALIDATION REPORT

Continued from previous page...

Mol	Chain	Residues	Atoms	AltConf
5	B	1	Total C Fe N O<br>86 68 2 8 8	0
5	E	1	Total C Fe N O<br>86 68 2 8 8	0
5	E	1	Total C Fe N O<br>86 68 2 8 8	0

- Molecule 6 is 2-[(2 {E},6 {E},10 {Z},14 {Z},18 {Z},23 {R})-3,7,11,15,19,23,27-heptamethyloctacosa-2,6,10,14,18-pentaenyl]naphthalene-1,4-dione (three-letter code: DLX) (formula: $C_{45}H_{66}O_2$).

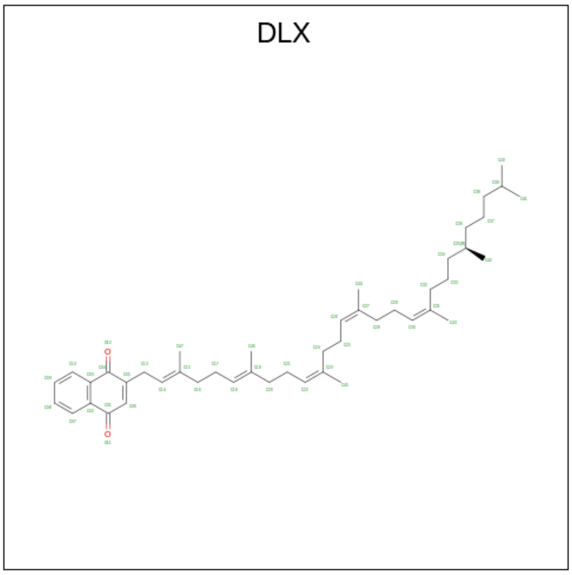

Mol	Chain	Residues	Atoms	AltConf
6	B	1	Total C O<br>94 90 4	0
6	B	1	Total C O<br>94 90 4	0
6	C	1	Total C O<br>47 45 2	0
6	E	1	Total C O<br>94 90 4	0
6	E	1	Total C O<br>94 90 4	0
6	F	1	Total C O<br>47 45 2	0

- Molecule 7 is (1R)-2-{[{[(2S)-2,3-DIHYDROXYPROPYL]OXY}(HYDROXY)PHOSPHORYL]OXY}-1-[(PALMITOYLOXY)METHYL]ETHYL (11E)-OCTADEC-11-ENOATE

CONFIDENTIAL VALIDATION REPORT

(three-letter code: PGV) (formula: $C_{40}H_{77}O_{10}P$).

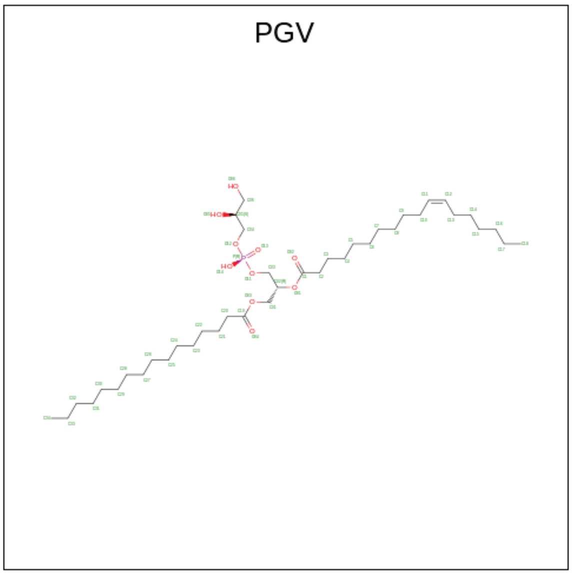

Mol	Chain	Residues	Atoms	AltConf
7	B	1	Total C O P<br>102 80 20 2	0
7	B	1	Total C O P<br>102 80 20 2	0
7	E	1	Total C O P<br>51 40 10 1	0

- Molecule 8 is HEME C (three-letter code: HEC) (formula: $C_{34}H_{34}FeN_4O_4$).

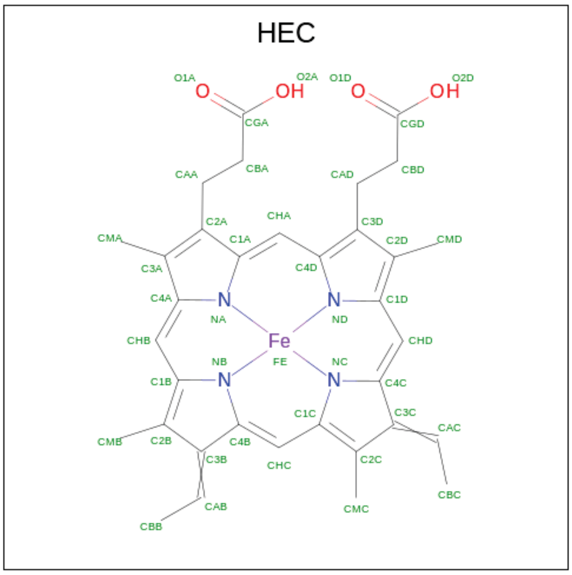

VALIDATION REPORT

Mol	Chain	Residues	Atoms					AltConf
8	C	1	Total<br>43	C<br>34	Fe<br>1	N<br>4	O<br>4	0
8	F	1	Total<br>43	C<br>34	Fe<br>1	N<br>4	O<br>4	0

CONFIDENTIAL VALIDATION REPORT

3 Residue-property plots (i)

These plots are drawn for all protein, RNA and DNA chains in the entry. The first graphic for a chain summarises the proportions of the various outlier classes displayed in the second graphic. The second graphic shows the sequence view annotated by issues in geometry. Residues are color-coded according to the number of geometric quality criteria for which they contain at least one outlier: green = 0, yellow = 1, orange = 2 and red = 3 or more. Stretches of 2 or more consecutive residues without any outlier are shown as a green connector. Residues present in the sample, but not in the model, are shown in grey.

- Molecule 1: Rieske-I iron sulfur protein

Chain A:

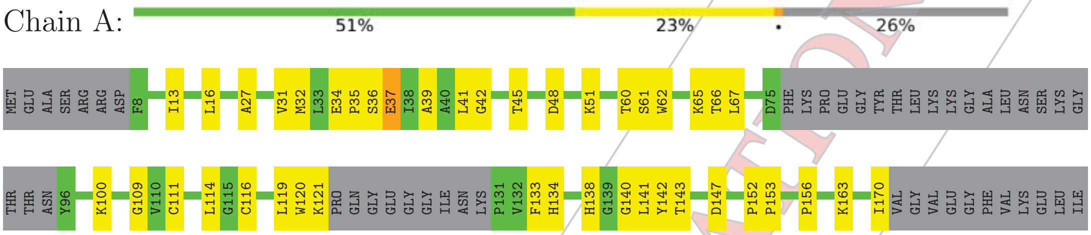

- Molecule 1: Rieske-I iron sulfur protein

Chain D:

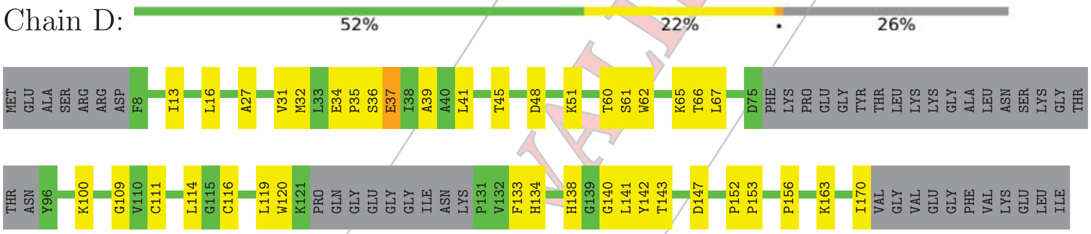

- Molecule 2: cytochrome b subunit

Chain B:

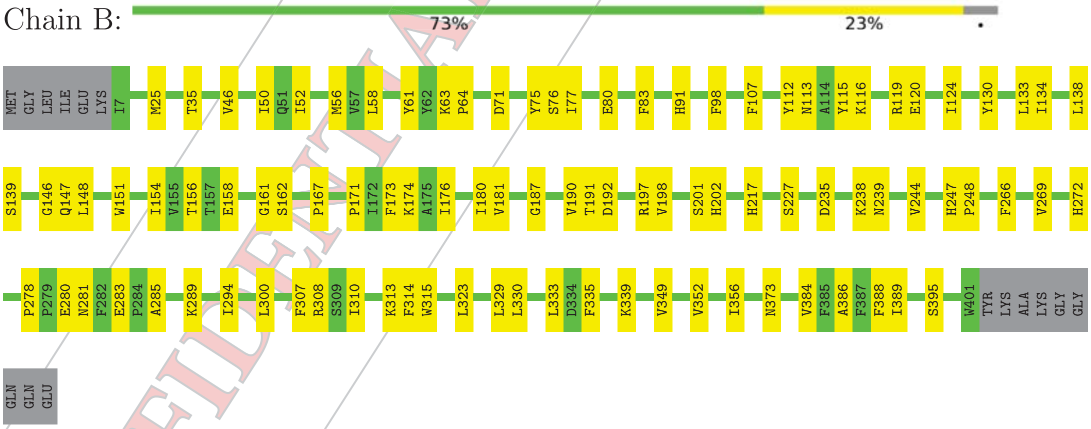

- Molecule 2: cytochrome b subunit

Chain E:

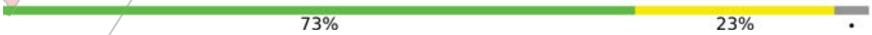

CONFIDENTIAL VALIDATION REPORT

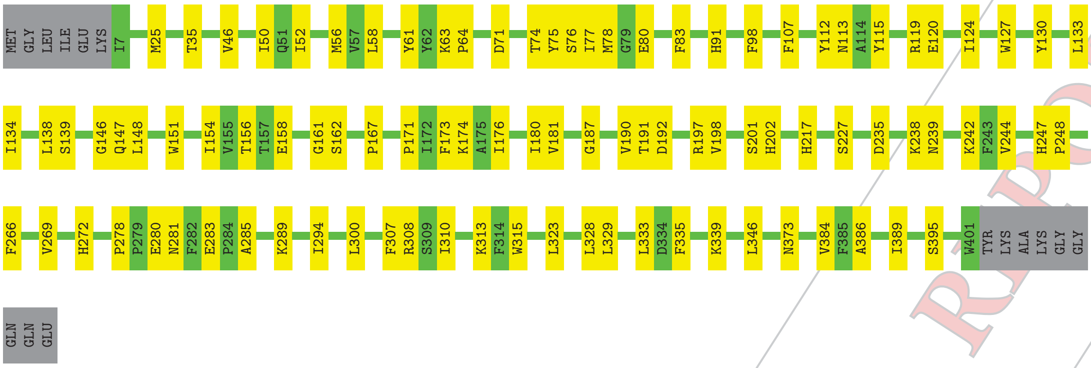

- Molecule 3: Cytochrome c

Chain C:

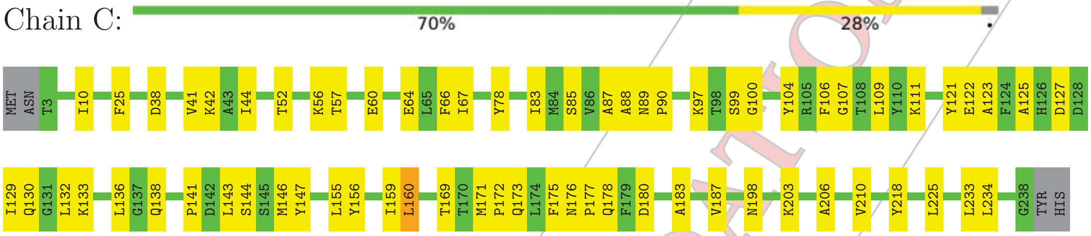

- Molecule 3: Cytochrome c

Chain F:

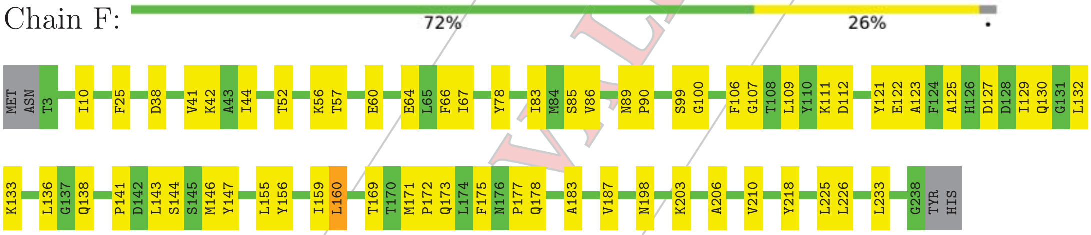

CONFIDENTIAL VALIDATION REPORT

4 Experimental information ⓘ

Property	Value	Source
Reconstruction method	SINGLE PARTICLE	Depositor
Imposed symmetry	POINT, Not provided	Depositor
Number of particles used	93622	Depositor
Resolution determination method	FSC 0.143 CUT-OFF	Depositor
CTF correction method	NONE	Depositor
Microscope	FEI TITAN KRIOS	Depositor
Voltage (kV)	300	Depositor
Electron dose $(e^{-}/\text{Å}^{2})$	60	Depositor
Minimum defocus (nm)	Not provided	Depositor
Maximum defocus (nm)	Not provided	Depositor
Magnification	Not provided	Depositor
Image detector	GATAN K2 SUMMIT (4k x 4k)	Depositor

CONFIDENTIAL VALIDATION REPORT

5 Model quality (i)

5.1 Standard geometry (i)

Bond lengths and bond angles in the following residue types are not validated in this section: PGV, HEM, DLX, FES, HEC

The Z score for a bond length (or angle) is the number of standard deviations the observed value is removed from the expected value. A bond length (or angle) with $|Z| > 5$ is considered an outlier worth inspection. RMSZ is the root-mean-square of all Z scores of the bond lengths (or angles).

Mol	Chain	Bond lengths		Bond angles	
		RMSZ	#|Z| >2	RMSZ	#|Z| >2
1	A	0.26	0/1039	0.48	0/1410
1	D	0.26	0/1039	0.48	0/1410
2	B	0.28	0/3346	0.44	0/4570
2	E	0.28	0/3346	0.44	0/4570
3	C	0.27	0/1974	0.48	0/2674
3	F	0.27	0/1974	0.48	0/2674
All	All	0.27	0/12718	0.46	0/17308

There are no bond length outliers.

There are no bond angle outliers.

There are no chirality outliers.

There are no planarity outliers.

5.2 Too-close contacts (i)

In the following table, the Non-H and H(model) columns list the number of non-hydrogen atoms and hydrogen atoms in the chain respectively. The H(added) column lists the number of hydrogen atoms added and optimized by MolProbity. The Clashes column lists the number of clashes within the asymmetric unit, whereas Symm-Clashes lists symmetry related clashes.

Mol	Chain	Non-H	H(model)	H(added)	Clashes	Symm-Clashes
1	A	1012	0	1057	36	0
1	D	1012	0	1057	32	0
2	B	3226	0	3280	66	0
2	E	3226	0	3280	65	0
3	C	1917	0	1929	49	0
3	F	1917	0	1929	48	0
4	A	4	0	0	1	0

Continued on next page...

CONFIDENTIAL VALIDATION REPORT

Continued from previous page...

Mol	Chain	Non-H	H(model)	H(added)	Clashes	Symm-Clashes
4	D	4	0	0	1	0
5	B	86	0	60	7	0
5	E	86	0	60	7	0
6	B	94	0	0	13	0
6	C	47	0	0	0	0
6	E	94	0	0	8	0
6	F	47	0	0	0	0
7	B	102	0	151	15	0
7	E	51	0	75	1	0
8	C	43	0	29	9	0
8	F	43	0	29	8	0
All	All	13011	0	12936	305	0

The all-atom clashscore is defined as the number of clashes found per 1000 atoms (including hydrogen atoms). The all-atom clashscore for this structure is 12.

All (305) close contacts within the same asymmetric unit are listed below, sorted by their clash magnitude.

Atom-1	Atom-2	Interatomic distance (Å)	Clash overlap (Å)
6:B:503:DLX:C23	7:B:506:PGV:H341	1.90	1.01
3:F:159:ILE:CD1	8:F:302:HEC:HMB2	1.99	0.93
3:C:159:ILE:CD1	8:C:302:HEC:HMB2	1.98	0.93
3:C:159:ILE:HD13	8:C:302:HEC:HMB2	1.53	0.89
3:F:159:ILE:HD13	8:F:302:HEC:HMB2	1.54	0.89
6:B:503:DLX:C24	7:B:506:PGV:C33	2.60	0.80
5:B:501:HEM:HHC	5:B:501:HEM:HBB2	1.66	0.77
5:E:501:HEM:HBB2	5:E:501:HEM:HHC	1.66	0.76
1:A:48:ASP:HB3	1:A:51:LYS:HG3	1.67	0.74
1:D:48:ASP:HB3	1:D:51:LYS:HG3	1.67	0.74
2:E:238:LYS:HD3	2:E:239:ASN:H	1.53	0.74
5:B:501:HEM:HBC2	5:B:501:HEM:HHD	1.70	0.73
2:E:335:PHE:O	2:E:395:SER:OG	2.06	0.73
2:B:335:PHE:O	2:B:395:SER:OG	2.06	0.73
5:E:501:HEM:HHD	5:E:501:HEM:HBC2	1.70	0.73
2:B:238:LYS:HD3	2:B:239:ASN:H	1.52	0.73
3:F:159:ILE:HD11	8:F:302:HEC:HMB2	1.75	0.69
2:E:167:PRO:HD2	2:E:171:PRO:HA	1.75	0.68
2:B:167:PRO:HD2	2:B:171:PRO:HA	1.75	0.66
2:B:248:PRO:HG3	3:C:233:LEU:HD22	1.77	0.66
1:A:140:GLY:HA2	1:A:152:PRO:HD2	1.78	0.66
3:C:159:ILE:HD11	8:C:302:HEC:HMB2	1.74	0.65

Continued on next page...

CONFIDENTIAL VALIDATION REPORT

Continued from previous page...

Atom-1	Atom-2	Interatomic distance (Å)	Clash overlap (Å)
6:B:503:DLX:C24	7:B:506:PGV:H331	2.25	0.65
2:E:248:PRO:HG3	3:F:233:LEU:HD22	1.77	0.65
3:F:129:ILE:HD11	3:F:133:LYS:HB3	1.78	0.65
2:E:310:ILE:HG21	2:E:315:TRP:HD1	1.62	0.65
3:F:177:PRO:O	3:F:178:GLN:HG2	1.97	0.64
1:D:140:GLY:HA2	1:D:152:PRO:HD2	1.78	0.64
1:D:62:TRP:HB2	1:D:67:LEU:HD22	1.79	0.64
3:C:129:ILE:HD11	3:C:133:LYS:HB3	1.78	0.64
2:B:310:ILE:HG21	2:B:315:TRP:HD1	1.62	0.64
3:C:177:PRO:O	3:C:178:GLN:HG2	1.97	0.64
3:C:169:THR:HG22	3:C:171:MET:H	1.62	0.64
1:A:134:HIS:HB3	1:A:141:LEU:HD23	1.79	0.64
1:A:62:TRP:HB2	1:A:67:LEU:HD22	1.79	0.64
1:D:134:HIS:HB3	1:D:141:LEU:HD23	1.79	0.63
7:B:506:PGV:H171	6:E:503:DLX:C24	2.29	0.63
3:C:41:VAL:HG23	3:C:42:LYS:H	1.62	0.62
3:F:169:THR:HG22	3:F:171:MET:H	1.62	0.62
3:F:41:VAL:HG23	3:F:42:LYS:H	1.63	0.62
1:D:13:ILE:HG22	3:F:225:LEU:HB3	1.82	0.62
3:C:160:LEU:HB3	3:C:187:VAL:HG21	1.81	0.62
3:F:160:LEU:HB3	3:F:187:VAL:HG21	1.81	0.61
1:A:13:ILE:HG22	3:C:225:LEU:HB3	1.82	0.61
6:B:503:DLX:C45	7:B:506:PGV:H341	2.30	0.60
7:B:506:PGV:H183	6:E:503:DLX:C46	2.32	0.59
2:B:162:SER:HB2	2:B:313:LYS:HB3	1.85	0.59
6:B:503:DLX:C23	7:B:506:PGV:C34	2.75	0.59
2:E:162:SER:HB2	2:E:313:LYS:HB3	1.85	0.59
2:B:113:ASN:ND2	2:B:339:LYS:O	2.32	0.58
2:E:113:ASN:ND2	2:E:339:LYS:O	2.32	0.58
2:B:187:GLY:HA3	1:D:39:ALA:HB1	1.85	0.58
5:B:502:HEM:HBC2	5:B:502:HEM:HMC2	1.84	0.58
1:D:34:GLU:OE2	1:D:36:SER:OG	2.21	0.58
3:C:147:TYR:HB3	3:C:198:ASN:HD22	1.69	0.58
3:C:160:LEU:O	3:C:173:GLN:NE2	2.37	0.58
1:A:34:GLU:OE2	1:A:36:SER:OG	2.21	0.57
3:F:147:TYR:HB3	3:F:198:ASN:HD22	1.69	0.57
3:F:160:LEU:O	3:F:173:GLN:NE2	2.37	0.57
1:D:31:VAL:HG22	2:E:83:PHE:HB2	1.87	0.57
2:B:238:LYS:HD3	2:B:239:ASN:N	2.19	0.57
2:E:154:ILE:O	2:E:158:GLU:HG2	2.05	0.57
2:B:154:ILE:O	2:B:158:GLU:HG2	2.05	0.56

Continued on next page...

CONFIDENTIAL VALIDATION REPORT

Continued from previous page...

Atom-1	Atom-2	Interatomic distance (Å)	Clash overlap (Å)
2:B:192:ASP:OD1	2:E:63:LYS:HE3	2.05	0.56
1:D:27:ALA:O	1:D:31:VAL:HG23	2.06	0.56
1:A:39:ALA:HB1	2:E:187:GLY:HA3	1.87	0.56
1:D:35:PRO:HB2	1:D:39:ALA:HB2	1.87	0.56
1:A:35:PRO:HB2	1:A:39:ALA:HB2	1.87	0.56
6:B:503:DLX:C24	7:B:506:PGV:H332	2.34	0.56
1:A:31:VAL:HG22	2:B:83:PHE:HB2	1.87	0.56
1:A:60:THR:HG22	1:A:61:SER:H	1.71	0.55
1:A:27:ALA:O	1:A:31:VAL:HG23	2.06	0.55
2:E:238:LYS:HD3	2:E:239:ASN:N	2.19	0.55
2:B:63:LYS:HE3	2:E:192:ASP:OD1	2.06	0.55
2:E:148:LEU:HD23	2:E:280:GLU:HG2	1.88	0.55
6:E:503:DLX:C44	6:E:503:DLX:C30	2.85	0.55
2:B:181:VAL:HA	2:B:187:GLY:HA2	1.89	0.54
1:D:60:THR:HG22	1:D:61:SER:H	1.71	0.54
1:D:138:HIS:HB2	4:D:501:FES:S2	2.48	0.54
2:B:148:LEU:HD23	2:B:280:GLU:HG2	1.88	0.54
6:B:503:DLX:C30	6:B:503:DLX:C44	2.85	0.54
6:E:503:DLX:C26	6:E:503:DLX:C45	2.85	0.54
1:A:138:HIS:HB2	4:A:501:FES:S2	2.48	0.53
3:C:175:PHE:HB3	3:C:183:ALA:HB1	1.90	0.53
2:E:181:VAL:HA	2:E:187:GLY:HA2	1.88	0.53
3:C:52:THR:OG1	3:C:56:LYS:O	2.24	0.53
2:B:35:THR:HG21	2:B:112:TYR:OH	2.09	0.53
2:E:35:THR:HG21	2:E:112:TYR:OH	2.09	0.53
2:B:138:LEU:HD11	2:B:156:THR:HG21	1.90	0.53
3:F:175:PHE:HB3	3:F:183:ALA:HB1	1.90	0.53
6:B:503:DLX:C45	6:B:503:DLX:C26	2.85	0.52
1:D:41:LEU:HD11	3:F:111:LYS:HE3	1.91	0.52
2:E:76:SER:HA	2:E:80:GLU:HB2	1.90	0.52
2:E:138:LEU:HD11	2:E:156:THR:HG21	1.91	0.52
2:B:139:SER:OG	2:B:202:HIS:HB2	2.09	0.52
2:B:76:SER:HA	2:B:80:GLU:HB2	1.90	0.52
2:E:139:SER:OG	2:E:202:HIS:HB2	2.09	0.52
3:C:172:PRO:HD3	8:C:302:HEC:HBC3	1.92	0.52
1:D:114:LEU:HD12	1:D:138:HIS:NE2	2.25	0.52
3:F:52:THR:OG1	3:F:56:LYS:O	2.24	0.51
1:A:41:LEU:HD11	3:C:111:LYS:HE3	1.91	0.51
1:A:114:LEU:HD12	1:A:138:HIS:NE2	2.25	0.51
2:B:161:GLY:HA3	2:B:180:ILE:HD11	1.93	0.51
2:B:138:LEU:HD12	2:B:300:LEU:HD11	1.92	0.51

Continued on next page...

CONFIDENTIAL VALIDATION REPORT

Continued from previous page...

Atom-1	Atom-2	Interatomic distance (Å)	Clash overlap (Å)
3:F:83:ILE:O	3:F:89:ASN:HB2	2.11	0.51
3:C:83:ILE:O	3:C:89:ASN:HB2	2.11	0.50
2:E:173:PHE:HA	2:E:176:ILE:HG12	1.94	0.50
5:E:502:HEM:HMC1	5:E:502:HEM:HBC2	1.93	0.50
3:F:172:PRO:HD3	8:F:302:HEC:HBC3	1.92	0.50
2:E:154:ILE:HD11	2:E:190:VAL:HG21	1.93	0.50
2:E:138:LEU:HD12	2:E:300:LEU:HD11	1.92	0.50
2:E:91:HIS:NE2	5:E:501:HEM:ND	2.60	0.50
6:B:503:DLX:C24	7:B:506:PGV:H341	2.41	0.50
2:B:173:PHE:HA	2:B:176:ILE:HG12	1.94	0.50
2:B:91:HIS:NE2	5:B:501:HEM:ND	2.60	0.50
2:E:161:GLY:HA3	2:E:180:ILE:HD11	1.93	0.49
6:B:503:DLX:C42	6:B:503:DLX:C32	2.90	0.49
2:B:154:ILE:HD11	2:B:190:VAL:HG21	1.93	0.49
2:B:272:HIS:CE1	3:C:25:PHE:HB3	2.48	0.49
6:E:503:DLX:C42	6:E:503:DLX:C32	2.90	0.49
3:C:206:ALA:O	3:C:210:VAL:HG23	2.13	0.49
3:C:146:MET:HG2	8:C:302:HEC:HMA3	1.95	0.49
3:F:206:ALA:O	3:F:210:VAL:HG23	2.13	0.48
3:C:87:ALA:HA	3:C:88:ALA:HA	1.61	0.48
2:E:107:PHE:HE2	7:E:505:PGV:H291	1.78	0.48
3:F:146:MET:HG2	8:F:302:HEC:HMA3	1.95	0.48
1:D:114:LEU:HD12	1:D:138:HIS:HE2	1.78	0.48
2:E:323:LEU:HD11	2:E:384:VAL:HG12	1.96	0.48
2:E:71:ASP:O	2:E:75:TYR:HB3	2.14	0.48
1:A:114:LEU:HD12	1:A:138:HIS:HE2	1.78	0.48
2:E:238:LYS:CD	2:E:239:ASN:H	2.26	0.48
2:E:98:PHE:CE2	2:E:133:LEU:HD11	2.49	0.48
1:D:143:THR:HG22	1:D:147:ASP:O	2.14	0.47
3:F:130:GLN:HG2	3:F:132:LEU:HG	1.96	0.47
2:E:272:HIS:CE1	3:F:25:PHE:HB3	2.48	0.47
1:A:45:THR:HG21	1:A:62:TRP:HD1	1.79	0.47
7:B:506:PGV:H22	6:E:504:DLX:C13	2.44	0.47
2:B:107:PHE:HE2	7:B:505:PGV:H291	1.78	0.47
2:B:71:ASP:O	2:B:75:TYR:HB3	2.14	0.47
2:E:151:TRP:HB3	2:E:294:ILE:HG12	1.97	0.47
1:D:45:THR:HG21	1:D:62:TRP:HD1	1.79	0.47
1:A:111:CYS:HB3	1:A:116:CYS:H	1.80	0.47
2:B:98:PHE:CE2	2:B:133:LEU:HD11	2.49	0.47
3:C:99:SER:N	3:C:100:GLY:HA3	2.30	0.47
1:D:100:LYS:HA	1:D:100:LYS:HD2	1.61	0.47

Continued on next page...

CONFIDENTIAL VALIDATION REPORT

Continued from previous page...

Atom-1	Atom-2	Interatomic distance (Å)	Clash overlap (Å)
3:F:78:TYR:CD2	3:F:144:SER:HB3	2.50	0.47
1:A:143:THR:HG22	1:A:147:ASP:O	2.14	0.46
3:C:172:PRO:HD3	8:C:302:HEC:CBC	2.46	0.46
2:B:323:LEU:HD11	2:B:384:VAL:HG12	1.96	0.46
2:E:307:PHE:O	2:E:308:ARG:HG3	2.15	0.46
5:B:502:HEM:HBB2	5:B:502:HEM:HMB1	1.96	0.46
1:D:111:CYS:HB3	1:D:116:CYS:H	1.80	0.46
2:E:146:GLY:O	2:E:285:ALA:HB2	2.16	0.46
3:F:109:LEU:HD23	3:F:109:LEU:H	1.81	0.46
3:F:89:ASN:ND2	3:F:125:ALA:HB1	2.31	0.46
2:B:307:PHE:O	2:B:308:ARG:HG3	2.15	0.46
2:B:120:GLU:O	2:B:124:ILE:HG12	2.16	0.46
3:C:106:PHE:HA	3:C:107:GLY:HA2	1.65	0.46
3:F:99:SER:N	3:F:100:GLY:HA3	2.30	0.46
3:C:130:GLN:HG2	3:C:132:LEU:HG	1.96	0.46
5:E:502:HEM:HMB1	5:E:502:HEM:HBB2	1.96	0.46
2:B:58:LEU:HD13	5:B:501:HEM:HBD1	1.97	0.46
2:E:386:ALA:HA	2:E:389:ILE:HG12	1.98	0.46
3:F:172:PRO:HD3	8:F:302:HEC:CBC	2.46	0.46
2:B:151:TRP:HB3	2:B:294:ILE:HG12	1.97	0.46
3:C:78:TYR:CD2	3:C:144:SER:HB3	2.50	0.46
2:E:120:GLU:O	2:E:124:ILE:HG12	2.16	0.46
2:E:58:LEU:HD13	5:E:501:HEM:HBD1	1.97	0.46
2:B:146:GLY:O	2:B:285:ALA:HB2	2.15	0.45
1:A:100:LYS:HD2	1:A:100:LYS:HA	1.61	0.45
1:D:120:TRP:HA	1:D:133:PHE:HB2	1.98	0.45
3:C:89:ASN:ND2	3:C:125:ALA:HB1	2.31	0.45
1:A:152:PRO:N	1:A:153:PRO:HD2	2.32	0.45
1:A:16:LEU:HG	3:C:218:TYR:CE1	2.52	0.45
1:D:163:LYS:HB2	1:D:170:ILE:HG13	1.98	0.45
2:E:329:LEU:O	2:E:333:LEU:N	2.50	0.45
3:F:121:TYR:O	3:F:122:GLU:HG2	2.17	0.45
1:D:16:LEU:HG	3:F:218:TYR:CE1	2.52	0.45
2:E:235:ASP:OD2	2:E:239:ASN:HB2	2.17	0.45
1:A:36:SER:O	1:A:37:GLU:HG3	2.17	0.45
2:B:329:LEU:O	2:B:333:LEU:N	2.50	0.45
3:C:57:THR:OG1	3:C:60:GLU:HG3	2.17	0.45
2:E:139:SER:HB2	2:E:198:VAL:HG13	1.99	0.45
3:C:109:LEU:H	3:C:109:LEU:HD23	1.81	0.45
1:D:65:LYS:HA	1:D:66:THR:HA	1.58	0.45
3:F:160:LEU:C	3:F:160:LEU:CD1	2.85	0.45

Continued on next page...

CONFIDENTIAL VALIDATION REPORT

Continued from previous page...

Atom-1	Atom-2	Interatomic distance (Å)	Clash overlap (Å)
1:A:111:CYS:SG	1:A:142:TYR:OH	2.69	0.44
1:A:120:TRP:HA	1:A:133:PHE:HB2	1.98	0.44
2:B:116:LYS:O	2:B:119:ARG:N	2.44	0.44
2:B:386:ALA:HA	2:B:389:ILE:HG12	1.98	0.44
3:C:121:TYR:O	3:C:122:GLU:HG2	2.17	0.44
3:C:160:LEU:C	3:C:160:LEU:CD1	2.85	0.44
1:A:163:LYS:HB2	1:A:170:ILE:HG13	1.99	0.44
6:B:503:DLX:C24	7:B:506:PGV:C34	2.95	0.44
2:E:61:TYR:HB2	2:E:77:ILE:HD11	1.99	0.44
2:B:235:ASP:OD2	2:B:239:ASN:HB2	2.17	0.44
3:F:57:THR:OG1	3:F:60:GLU:HG3	2.17	0.44
1:D:152:PRO:N	1:D:153:PRO:HD2	2.32	0.44
6:E:503:DLX:C38	6:E:503:DLX:C42	2.96	0.44
8:C:302:HEC:HBA2	8:C:302:HEC:HHA	2.00	0.44
3:C:60:GLU:O	3:C:64:GLU:HG2	2.18	0.44
1:D:36:SER:O	1:D:37:GLU:HG3	2.17	0.44
2:B:61:TYR:HB2	2:B:77:ILE:HD11	1.99	0.43
3:F:38:ASP:HB2	3:F:156:TYR:CE2	2.53	0.43
6:B:503:DLX:C42	6:B:503:DLX:C38	2.96	0.43
3:C:159:ILE:HD13	8:C:302:HEC:CMB	2.38	0.43
3:C:41:VAL:HB	3:C:44:ILE:HG12	2.01	0.43
1:A:66:THR:OG1	1:A:109:GLY:O	2.36	0.43
2:B:278:PRO:HG2	2:B:281:ASN:HB2	2.00	0.43
1:D:66:THR:OG1	1:D:109:GLY:O	2.36	0.43
3:C:38:ASP:HB2	3:C:156:TYR:CE2	2.53	0.43
1:A:41:LEU:HD12	1:A:41:LEU:HA	1.90	0.43
2:E:130:TYR:CZ	2:E:134:ILE:HD11	2.54	0.43
2:B:139:SER:HB2	2:B:198:VAL:HG13	1.99	0.43
2:B:52:ILE:O	2:B:56:MET:HG3	2.19	0.43
3:C:64:GLU:HA	3:C:67:ILE:HG12	2.01	0.42
3:F:64:GLU:HA	3:F:67:ILE:HG12	2.00	0.42
2:B:217:HIS:NE2	5:B:502:HEM:ND	2.67	0.42
3:F:130:GLN:N	3:F:130:GLN:OE1	2.52	0.42
3:F:60:GLU:O	3:F:64:GLU:HG2	2.18	0.42
3:F:41:VAL:HB	3:F:44:ILE:HG12	2.00	0.42
2:B:247:HIS:CE1	2:B:248:PRO:HB3	2.55	0.42
6:B:503:DLX:C22	7:B:506:PGV:H341	2.45	0.42
2:B:289:LYS:HE2	1:D:114:LEU:HD22	2.01	0.42
2:E:191:THR:OG1	2:E:192:ASP:N	2.52	0.42
2:E:266:PHE:HA	2:E:269:VAL:HB	2.00	0.42
2:E:278:PRO:HG2	2:E:281:ASN:HB2	2.00	0.42

Continued on next page...

CONFIDENTIAL VALIDATION REPORT

Continued from previous page...

Atom-1	Atom-2	Interatomic distance (Å)	Clash overlap (Å)
2:E:308:ARG:HH22	2:E:373:ASN:ND2	2.18	0.42
2:E:52:ILE:O	2:E:56:MET:HG3	2.19	0.42
8:F:302:HEC:HHA	8:F:302:HEC:HBA2	2.00	0.42
2:B:130:TYR:CZ	2:B:134:ILE:HD11	2.54	0.42
3:C:130:GLN:OE1	3:C:130:GLN:N	2.52	0.42
3:F:155:LEU:O	3:F:159:ILE:HG13	2.20	0.42
3:C:155:LEU:O	3:C:159:ILE:HG13	2.19	0.42
1:D:111:CYS:SG	1:D:142:TYR:OH	2.69	0.42
1:D:147:ASP:HA	1:D:156:PRO:HB3	2.01	0.42
2:E:113:ASN:HA	2:E:115:TYR:CE2	2.55	0.42
2:E:201:SER:OG	2:E:202:HIS:N	2.53	0.42
1:A:119:LEU:O	1:A:133:PHE:HB2	2.19	0.42
2:B:113:ASN:HA	2:B:115:TYR:CE2	2.55	0.42
2:B:266:PHE:HA	2:B:269:VAL:HB	2.01	0.42
7:B:506:PGV:H101	7:B:506:PGV:H131	1.83	0.42
3:C:136:LEU:HD21	8:C:302:HEC:HAC	2.02	0.42
2:E:119:ARG:CZ	2:E:227:SER:HB3	2.50	0.42
1:A:32:MET:O	2:E:197:ARG:HD3	2.20	0.42
2:E:217:HIS:NE2	5:E:502:HEM:ND	2.67	0.42
2:B:25:MET:HB3	2:B:247:HIS:HB2	2.02	0.42
3:C:85:SER:O	3:C:85:SER:OG	2.38	0.42
2:B:197:ARG:HD3	1:D:32:MET:O	2.20	0.42
2:B:191:THR:OG1	2:B:192:ASP:N	2.52	0.41
2:B:201:SER:OG	2:B:202:HIS:N	2.53	0.41
2:B:244:VAL:O	2:B:244:VAL:HG13	2.20	0.41
3:C:89:ASN:N	3:C:90:PRO:HD2	2.35	0.41
3:C:97:LYS:HD3	3:C:97:LYS:HA	1.78	0.41
2:E:247:HIS:CE1	2:E:248:PRO:HB3	2.54	0.41
2:E:46:VAL:O	2:E:50:ILE:HG12	2.21	0.41
2:B:64:PRO:HD2	2:E:64:PRO:HD2	2.02	0.41
3:F:112:ASP:N	3:F:112:ASP:OD1	2.53	0.41
3:F:171:MET:HA	3:F:172:PRO:HD3	1.94	0.41
2:B:238:LYS:CD	2:B:239:ASN:H	2.26	0.41
3:C:138:GLN:HB3	1:D:116:CYS:SG	2.60	0.41
3:F:226:LEU:HD23	3:F:226:LEU:HA	1.95	0.41
1:A:41:LEU:HA	1:A:42:GLY:HA2	1.68	0.41
2:B:310:ILE:HD13	2:B:315:TRP:CD1	2.56	0.41
1:A:114:LEU:HD22	2:E:289:LYS:HE2	2.01	0.41
1:A:121:LYS:HE2	1:A:121:LYS:HB2	1.91	0.41
1:D:119:LEU:O	1:D:133:PHE:HB2	2.19	0.41
3:F:89:ASN:N	3:F:90:PRO:HD2	2.35	0.41

Continued on next page...

CONFIDENTIAL VALIDATION REPORT

Continued from previous page...

Atom-1	Atom-2	Interatomic distance (Å)	Clash overlap (Å)
1:A:65:LYS:HA	1:A:66:THR:HA	1.58	0.41
2:B:294:ILE:HA	2:B:294:ILE:HD12	1.91	0.41
3:C:234:LEU:HD23	3:C:234:LEU:HA	1.93	0.41
3:F:136:LEU:HD21	8:F:302:HEC:HAC	2.02	0.41
3:F:106:PHE:HA	3:F:107:GLY:HA2	1.65	0.41
2:B:119:ARG:CZ	2:B:227:SER:HB3	2.50	0.41
2:B:349:VAL:O	2:B:352:VAL:HG12	2.21	0.41
2:E:113:ASN:HB2	2:E:339:LYS:O	2.21	0.41
2:E:127:TRP:CD2	2:E:328:LEU:HG	2.56	0.41
7:B:506:PGV:C18	6:E:503:DLX:C46	2.98	0.41
2:B:147:GLN:N	2:B:283:GLU:O	2.54	0.41
2:B:356:ILE:HA	2:B:356:ILE:HD13	1.96	0.41
2:B:46:VAL:O	2:B:50:ILE:HG12	2.21	0.41
2:E:25:MET:HB3	2:E:247:HIS:HB2	2.02	0.41
1:A:147:ASP:HA	1:A:156:PRO:HB3	2.01	0.41
2:E:242:LYS:HB2	2:E:242:LYS:HE2	1.95	0.41
2:E:154:ILE:HD13	2:E:154:ILE:HA	1.91	0.40
3:F:123:ALA:HA	3:F:127:ASP:HB2	2.02	0.40
3:C:104:TYR:HA	3:C:109:LEU:HA	2.03	0.40
3:C:176:ASN:O	3:C:180:ASP:HB2	2.22	0.40
2:E:244:VAL:O	2:E:244:VAL:HG13	2.20	0.40
2:E:74:THR:O	2:E:78:MET:HB2	2.21	0.40
3:F:85:SER:HA	3:F:86:VAL:HA	1.83	0.40
1:A:114:LEU:HD23	1:A:114:LEU:HA	1.82	0.40
2:B:113:ASN:HB2	2:B:339:LYS:O	2.21	0.40
3:C:123:ALA:HA	3:C:127:ASP:HB2	2.02	0.40
3:C:66:PHE:CE1	3:C:143:LEU:HD12	2.57	0.40
2:E:147:GLN:N	2:E:283:GLU:O	2.54	0.40
2:E:346:LEU:HD12	2:E:346:LEU:HA	1.96	0.40
2:B:308:ARG:HH22	2:B:373:ASN:ND2	2.18	0.40
2:B:330:LEU:HD12	2:B:388:PHE:HE1	1.87	0.40
2:B:167:PRO:HB3	2:B:314:PHE:CE2	2.57	0.40
2:E:310:ILE:HD13	2:E:315:TRP:CD1	2.56	0.40
3:F:132:LEU:O	3:F:136:LEU:HB2	2.22	0.40
1:A:116:CYS:SG	3:F:138:GLN:HB3	2.62	0.40
3:F:41:VAL:HG23	3:F:42:LYS:N	2.34	0.40
3:F:66:PHE:CE1	3:F:143:LEU:HD12	2.57	0.40

There are no symmetry-related clashes.

CONFIDENTIAL VALIDATION REPORT

5.3 Torsion angles (i)

5.3.1 Protein backbone (i)

In the following table, the Percentiles column shows the percent Ramachandran outliers of the chain as a percentile score with respect to all PDB entries followed by that with respect to all EM entries.

The Analysed column shows the number of residues for which the backbone conformation was analysed, and the total number of residues.

Mol	Chain	Analysed	Favoured	Allowed	Outliers	Percentiles	
1	A	128/181 (71%)	111 (87%)	17 (13%)	0	100	100
1	D	128/181 (71%)	111 (87%)	17 (13%)	0	100	100
2	B	393/410 (96%)	358 (91%)	35 (9%)	0	100	100
2	E	393/410 (96%)	359 (91%)	34 (9%)	0	100	100
3	C	234/240 (98%)	204 (87%)	29 (12%)	1 (0%)	36	69
3	F	234/240 (98%)	204 (87%)	29 (12%)	1 (0%)	36	69
All	All	1510/1662 (91%)	1347 (89%)	161 (11%)	2 (0%)	56	84

All (2) Ramachandran outliers are listed below:

Mol	Chain	Res	Type
3	C	141	PRO
3	F	141	PRO

5.3.2 Protein sidechains (i)

In the following table, the Percentiles column shows the percent sidechain outliers of the chain as a percentile score with respect to all PDB entries followed by that with respect to all EM entries.

The Analysed column shows the number of residues for which the sidechain conformation was analysed, and the total number of residues.

Mol	Chain	Analysed	Rotameric	Outliers	Percentiles	
1	A	108/145 (74%)	107 (99%)	1 (1%)	81	89
1	D	108/145 (74%)	107 (99%)	1 (1%)	81	89
2	B	339/350 (97%)	338 (100%)	1 (0%)	93	97
2	E	339/350 (97%)	338 (100%)	1 (0%)	93	97
3	C	206/210 (98%)	203 (98%)	3 (2%)	67	84

Continued on next page...

CONFIDENTIAL VALIDATION REPORT

Continued from previous page...

Mol	Chain	Analysed	Rotameric	Outliers	Percentiles	
3	F	206/210 (98%)	203 (98%)	3 (2%)	67	84
All	All	1306/1410 (93%)	1296 (99%)	10 (1%)	84	91

All (10) residues with a non-rotameric sidechain are listed below:

Mol	Chain	Res	Type
1	A	37	GLU
2	B	174	LYS
3	C	10	ILE
3	C	160	LEU
3	C	203	LYS
1	D	37	GLU
2	E	174	LYS
3	F	10	ILE
3	F	160	LEU
3	F	203	LYS

Some sidechains can be flipped to improve hydrogen bonding and reduce clashes. All (16) such sidechains are listed below:

Mol	Chain	Res	Type
1	A	113	HIS
2	B	24	GLN
2	B	51	GLN
2	B	96	ASN
2	B	147	GLN
2	B	373	ASN
3	C	176	ASN
3	C	198	ASN
1	D	113	HIS
2	E	24	GLN
2	E	51	GLN
2	E	96	ASN
2	E	147	GLN
2	E	373	ASN
3	F	176	ASN
3	F	198	ASN

5.3.3 RNA ⓘ

There are no RNA molecules in this entry.

CONFIDENTIAL VALIDATION REPORT

5.4 Non-standard residues in protein, DNA, RNA chains (i)

There are no non-standard protein/DNA/RNA residues in this entry.

5.5 Carbohydrates (i)

There are no carbohydrates in this entry.

5.6 Ligand geometry (i)

17 ligands are modelled in this entry.

In the following table, the Counts columns list the number of bonds (or angles) for which Mogul statistics could be retrieved, the number of bonds (or angles) that are observed in the model and the number of bonds (or angles) that are defined in the Chemical Component Dictionary. The Link column lists molecule types, if any, to which the group is linked. The Z score for a bond length (or angle) is the number of standard deviations the observed value is removed from the expected value. A bond length (or angle) with $|Z| > 2$ is considered an outlier worth inspection. RMSZ is the root-mean-square of all Z scores of the bond lengths (or angles).

Mol	Type	Chain	Res	Link	Bond lengths			Bond angles		
					Counts	RMSZ	$\#\|Z\|>2$	Counts	RMSZ	$\#\|Z\|>2$
4	FES	A	501	1	0,4,4	0.00	-	-		
5	HEM	B	501	2	27,50,50	1.92	4 (14%)	17,82,82	1.31	1 (5%)
5	HEM	B	502	2	27,50,50	1.77	4 (14%)	17,82,82	1.54	4 (23%)
6	DLX	B	503	-	48,48,48	1.56	6 (12%)	59,61,61	1.62	14 (23%)
6	DLX	B	504	-	48,48,48	4.51	27 (56%)	59,61,61	3.11	22 (37%)
7	PGV	B	505	2	50,50,50	1.20	4 (8%)	53,56,56	0.86	2 (3%)
7	PGV	B	506	-	50,50,50	0.88	3 (6%)	53,56,56	0.86	3 (5%)
6	DLX	C	301	-	48,48,48	4.48	27 (56%)	59,61,61	3.11	23 (38%)
8	HEC	C	302	3	26,50,50	2.35	3 (11%)	18,82,82	1.57	4 (22%)
4	FES	D	501	1	0,4,4	0.00	-	-		
5	HEM	E	501	2	27,50,50	1.92	4 (14%)	17,82,82	1.31	1 (5%)
5	HEM	E	502	2	27,50,50	1.77	4 (14%)	17,82,82	1.52	4 (23%)
6	DLX	E	503	-	48,48,48	1.56	6 (12%)	59,61,61	1.63	14 (23%)
6	DLX	E	504	-	48,48,48	4.51	27 (56%)	59,61,61	3.11	22 (37%)
7	PGV	E	505	2	50,50,50	1.20	4 (8%)	53,56,56	0.86	2 (3%)
6	DLX	F	301	-	48,48,48	4.48	27 (56%)	59,61,61	3.12	23 (38%)
8	HEC	F	302	3	26,50,50	2.35	3 (11%)	18,82,82	1.57	4 (22%)

In the following table, the Chirals column lists the number of chiral outliers, the number of chiral

CONFIDENTIAL VALIDATION REPORT

centers analysed, the number of these observed in the model and the number defined in the Chemical Component Dictionary. Similar counts are reported in the Torsion and Rings columns. '-' means no outliers of that kind were identified.

Mol	Type	Chain	Res	Link	Chirals	Torsions	Rings
4	FES	A	501	1	-	-	0/1/1/1
5	HEM	B	501	2	-	1/6/54/54	-
5	HEM	B	502	2	-	0/6/54/54	-
6	DLX	B	503	-	-	10/41/57/57	0/2/2/2
6	DLX	B	504	-	-	22/41/57/57	0/2/2/2
7	PGV	B	505	2	-	20/55/55/55	-
7	PGV	B	506	-	-	34/55/55/55	-
6	DLX	C	301	-	-	23/41/57/57	0/2/2/2
8	HEC	C	302	3	-	2/6/54/54	-
4	FES	D	501	1	-	-	0/1/1/1
5	HEM	E	501	2	-	1/6/54/54	-
5	HEM	E	502	2	-	0/6/54/54	-
6	DLX	E	503	-	-	10/41/57/57	0/2/2/2
6	DLX	E	504	-	-	22/41/57/57	0/2/2/2
7	PGV	E	505	2	-	20/55/55/55	-
6	DLX	F	301	-	-	23/41/57/57	0/2/2/2
8	HEC	F	302	3	-	2/6/54/54	-

All (153) bond length outliers are listed below:

Mol	Chain	Res	Type	Atoms	Z	Observed(Å)	Ideal(Å)
6	B	504	DLX	C30-C31	13.67	1.66	1.33
6	E	504	DLX	C30-C31	13.67	1.66	1.33
6	F	301	DLX	C30-C31	13.59	1.66	1.33
6	C	301	DLX	C30-C31	13.57	1.66	1.33
6	E	504	DLX	C22-C23	11.16	1.60	1.33
6	B	504	DLX	C22-C23	11.16	1.60	1.33
6	F	301	DLX	C22-C23	11.07	1.59	1.33
6	C	301	DLX	C22-C23	11.06	1.59	1.33
6	C	301	DLX	C26-C27	10.44	1.58	1.33
6	B	504	DLX	C26-C27	10.43	1.58	1.33
6	E	504	DLX	C26-C27	10.42	1.58	1.33
6	F	301	DLX	C26-C27	10.41	1.58	1.33
6	B	504	DLX	O11-C01	9.51	1.45	1.24
6	E	504	DLX	O11-C01	9.49	1.45	1.24
6	F	301	DLX	O11-C01	9.46	1.45	1.24
6	C	301	DLX	O11-C01	9.46	1.45	1.24

Continued on next page...

CONFIDENTIAL VALIDATION REPORT

Continued from previous page...

Mol	Chain	Res	Type	Atoms	Z	Observed(Å)	Ideal(Å)
6	B	504	DLX	C18-C19	8.97	1.54	1.33
6	E	504	DLX	C18-C19	8.95	1.54	1.33
6	F	301	DLX	C18-C19	8.90	1.54	1.33
6	C	301	DLX	C18-C19	8.87	1.54	1.33
6	E	504	DLX	C14-C15	8.28	1.53	1.33
6	B	504	DLX	C14-C15	8.24	1.53	1.33
6	C	301	DLX	C14-C15	8.23	1.53	1.33
6	F	301	DLX	C14-C15	8.21	1.53	1.33
8	C	302	HEC	C3B-C2B	-6.35	1.34	1.40
8	C	302	HEC	C3C-C2C	-6.35	1.34	1.40
8	F	302	HEC	C3C-C2C	-6.35	1.34	1.40
6	C	301	DLX	C32-C31	6.33	1.64	1.51
8	F	302	HEC	C3B-C2B	-6.33	1.34	1.40
6	F	301	DLX	C32-C31	6.30	1.64	1.51
6	B	504	DLX	C32-C31	6.23	1.64	1.51
6	E	504	DLX	C32-C31	6.23	1.64	1.51
6	B	504	DLX	O12-C04	5.93	1.35	1.23
6	E	504	DLX	O12-C04	5.93	1.35	1.23
6	F	301	DLX	O12-C04	5.89	1.35	1.23
6	C	301	DLX	O12-C04	5.86	1.35	1.23
6	E	504	DLX	C13-C05	5.42	1.56	1.51
6	B	504	DLX	C13-C05	5.40	1.56	1.51
8	F	302	HEC	C3D-C2D	5.35	1.53	1.37
8	C	302	HEC	C3D-C2D	5.33	1.53	1.37
6	E	503	DLX	C02-C01	-5.27	1.39	1.48
6	B	503	DLX	C02-C01	-5.22	1.39	1.48
6	F	301	DLX	C13-C05	5.20	1.56	1.51
6	C	301	DLX	C13-C05	5.20	1.56	1.51
6	E	503	DLX	C05-C04	-5.03	1.38	1.48
6	E	503	DLX	C03-C04	-5.02	1.38	1.48
6	B	503	DLX	C03-C04	-5.02	1.38	1.48
5	E	501	HEM	C3C-C2C	-5.00	1.33	1.40
5	B	501	HEM	C3C-C2C	-5.00	1.33	1.40
6	B	503	DLX	C05-C04	-5.00	1.38	1.48
5	E	501	HEM	C3B-C2B	-4.85	1.33	1.40
5	B	501	HEM	C3B-C2B	-4.85	1.33	1.40
6	E	504	DLX	C29-C30	4.48	1.65	1.50
6	C	301	DLX	C05-C04	-4.48	1.39	1.48
6	F	301	DLX	C05-C04	-4.47	1.39	1.48
6	B	504	DLX	C29-C30	4.47	1.65	1.50
6	F	301	DLX	C29-C30	4.46	1.65	1.50
6	C	301	DLX	C29-C30	4.46	1.65	1.50

Continued on next page...

CONFIDENTIAL VALIDATION REPORT

Continued from previous page...

Mol	Chain	Res	Type	Atoms	Z	Observed(Å)	Ideal(Å)
6	E	504	DLX	C05-C04	-4.40	1.39	1.48
6	B	504	DLX	C05-C04	-4.36	1.39	1.48
6	F	301	DLX	C28-C27	4.13	1.60	1.51
6	C	301	DLX	C28-C27	4.13	1.60	1.51
6	B	504	DLX	C28-C27	4.08	1.60	1.51
6	E	504	DLX	C28-C27	4.08	1.60	1.51
5	E	502	HEM	C3B-C2B	-3.89	1.35	1.40
6	B	504	DLX	C43-C31	3.86	1.60	1.50
5	B	502	HEM	C3C-C2C	-3.85	1.35	1.40
6	E	504	DLX	C43-C31	3.85	1.60	1.50
5	E	502	HEM	C3C-C2C	-3.85	1.35	1.40
6	C	301	DLX	C43-C31	3.84	1.60	1.50
5	B	502	HEM	C3B-C2B	-3.83	1.35	1.40
6	F	301	DLX	C43-C31	3.82	1.60	1.50
5	E	502	HEM	C3C-CAC	3.69	1.55	1.47
5	B	502	HEM	C3C-CAC	3.68	1.55	1.47
7	B	505	PGV	O03-C19	3.60	1.44	1.33
7	E	505	PGV	O03-C19	3.58	1.44	1.33
5	B	502	HEM	C3B-CAB	3.56	1.55	1.47
5	E	502	HEM	C3B-CAB	3.52	1.55	1.47
5	B	501	HEM	C3B-CAB	3.45	1.55	1.47
5	E	501	HEM	C3B-CAB	3.45	1.55	1.47
6	F	301	DLX	C44-C27	3.42	1.59	1.50
6	C	301	DLX	C44-C27	3.42	1.59	1.50
5	E	501	HEM	C3C-CAC	3.42	1.54	1.47
5	B	501	HEM	C3C-CAC	3.42	1.54	1.47
6	E	504	DLX	C28-C29	3.37	1.65	1.53
6	B	504	DLX	C28-C29	3.36	1.65	1.53
6	B	504	DLX	C10-C03	3.35	1.45	1.39
6	E	504	DLX	C10-C03	3.35	1.45	1.39
6	F	301	DLX	C28-C29	3.34	1.65	1.53
6	B	504	DLX	C44-C27	3.34	1.59	1.50
6	C	301	DLX	C28-C29	3.33	1.64	1.53
6	E	504	DLX	C44-C27	3.33	1.59	1.50
6	B	504	DLX	C25-C26	3.25	1.61	1.50
6	C	301	DLX	C10-C03	3.25	1.45	1.39
6	E	504	DLX	C25-C26	3.23	1.61	1.50
6	F	301	DLX	C10-C03	3.21	1.45	1.39
6	F	301	DLX	C25-C26	3.20	1.61	1.50
6	C	301	DLX	C25-C26	3.19	1.61	1.50
6	B	504	DLX	C13-C14	3.15	1.55	1.50
6	F	301	DLX	C13-C14	3.13	1.55	1.50

Continued on next page...

CONFIDENTIAL VALIDATION REPORT

Continued from previous page...

Mol	Chain	Res	Type	Atoms	Z	Observed(Å)	Ideal(Å)
6	E	504	DLX	C13-C14	3.13	1.55	1.50
7	E	505	PGV	O01-C1	3.13	1.43	1.34
7	B	505	PGV	O01-C1	3.13	1.43	1.34
6	C	301	DLX	C13-C14	3.11	1.55	1.50
6	E	504	DLX	C16-C15	3.06	1.57	1.51
6	B	504	DLX	C16-C15	3.06	1.57	1.51
6	F	301	DLX	C06-C05	3.04	1.42	1.34
6	B	504	DLX	C06-C05	3.02	1.42	1.34
6	E	504	DLX	C06-C05	3.02	1.42	1.34
6	C	301	DLX	C06-C05	3.01	1.42	1.34
6	F	301	DLX	C16-C15	2.95	1.57	1.51
6	C	301	DLX	C16-C15	2.95	1.57	1.51
6	E	504	DLX	C07-C02	2.91	1.44	1.39
6	B	504	DLX	C07-C02	2.90	1.44	1.39
6	B	503	DLX	C06-C01	-2.89	1.38	1.44
6	E	503	DLX	C06-C01	-2.85	1.38	1.44
6	C	301	DLX	C07-C02	2.81	1.44	1.39
6	F	301	DLX	C07-C02	2.80	1.44	1.39
6	C	301	DLX	C24-C23	2.75	1.57	1.51
6	E	504	DLX	C24-C23	2.74	1.57	1.51
6	F	301	DLX	C24-C23	2.71	1.57	1.51
6	B	504	DLX	C24-C23	2.70	1.57	1.51
6	E	504	DLX	C06-C01	-2.59	1.39	1.44
6	F	301	DLX	C06-C01	-2.57	1.39	1.44
6	B	504	DLX	C06-C01	-2.57	1.39	1.44
6	C	301	DLX	C06-C01	-2.56	1.39	1.44
6	B	504	DLX	C03-C04	-2.53	1.43	1.48
6	B	503	DLX	O11-C01	-2.52	1.18	1.24
6	E	504	DLX	C03-C04	-2.52	1.43	1.48
6	E	503	DLX	O11-C01	-2.51	1.18	1.24
6	E	504	DLX	C17-C18	2.50	1.58	1.50
6	B	504	DLX	C17-C18	2.47	1.58	1.50
6	F	301	DLX	C03-C04	-2.45	1.43	1.48
6	C	301	DLX	C03-C04	-2.45	1.43	1.48
7	B	506	PGV	O01-C02	-2.44	1.40	1.46
6	F	301	DLX	C17-C18	2.44	1.58	1.50
6	C	301	DLX	C17-C18	2.44	1.58	1.50
6	C	301	DLX	C02-C01	-2.40	1.44	1.48
6	B	504	DLX	C21-C22	2.39	1.58	1.50
6	E	504	DLX	C21-C22	2.38	1.58	1.50
7	B	506	PGV	O03-C19	2.37	1.40	1.33
6	E	504	DLX	C02-C01	-2.36	1.44	1.48

Continued on next page...

CONFIDENTIAL VALIDATION REPORT

Continued from previous page...

Mol	Chain	Res	Type	Atoms	Z	Observed(Å)	Ideal(Å)
6	F	301	DLX	C02-C01	-2.35	1.44	1.48
6	B	504	DLX	C02-C01	-2.34	1.44	1.48
6	F	301	DLX	C21-C22	2.28	1.58	1.50
6	C	301	DLX	C21-C22	2.28	1.58	1.50
6	B	503	DLX	O12-C04	-2.23	1.18	1.23
6	E	503	DLX	O12-C04	-2.22	1.18	1.23
7	B	505	PGV	O01-C02	-2.08	1.41	1.46
7	E	505	PGV	O01-C02	-2.07	1.41	1.46
7	B	506	PGV	O01-C1	2.07	1.40	1.34
7	E	505	PGV	C20-C19	2.02	1.56	1.50
7	B	505	PGV	C20-C19	2.01	1.56	1.50

All (143) bond angle outliers are listed below:

Mol	Chain	Res	Type	Atoms	Z	Observed(^{o})	Ideal(^{o})
6	F	301	DLX	C13-C14-C15	-8.66	112.37	126.79
6	C	301	DLX	C13-C14-C15	-8.65	112.39	126.79
6	E	504	DLX	C13-C14-C15	-8.51	112.63	126.79
6	B	504	DLX	C13-C14-C15	-8.50	112.64	126.79
6	F	301	DLX	C17-C18-C19	-6.61	111.43	127.67
6	C	301	DLX	C17-C18-C19	-6.59	111.48	127.67
6	C	301	DLX	C21-C22-C23	-6.45	111.83	127.67
6	F	301	DLX	C21-C22-C23	-6.44	111.86	127.67
6	B	504	DLX	C17-C18-C19	-6.33	112.12	127.67
6	E	504	DLX	C17-C18-C19	-6.33	112.12	127.67
6	F	301	DLX	C25-C26-C27	-6.17	112.52	127.67
6	C	301	DLX	C25-C26-C27	-6.16	112.53	127.67
6	B	504	DLX	C21-C22-C23	-6.16	112.54	127.67
6	E	504	DLX	C21-C22-C23	-6.15	112.57	127.67
6	E	504	DLX	C29-C30-C31	-6.06	112.78	127.67
6	B	504	DLX	C29-C30-C31	-6.05	112.81	127.67
6	C	301	DLX	C29-C30-C31	-5.98	112.97	127.67
6	E	504	DLX	C25-C26-C27	-5.98	112.99	127.67
6	F	301	DLX	C29-C30-C31	-5.98	112.99	127.67
6	B	504	DLX	C25-C26-C27	-5.97	113.00	127.67
6	C	301	DLX	C16-C15-C14	-5.75	109.29	121.11
6	F	301	DLX	C16-C15-C14	-5.75	109.29	121.11
6	F	301	DLX	C46-C19-C18	-5.64	109.03	123.68
6	C	301	DLX	C46-C19-C18	-5.62	109.08	123.68
6	B	504	DLX	C46-C19-C18	-5.59	109.16	123.68
6	E	504	DLX	C46-C19-C18	-5.58	109.18	123.68
6	B	504	DLX	C20-C19-C18	-5.56	109.68	121.11

Continued on next page...

CONFIDENTIAL VALIDATION REPORT

Continued from previous page...

Mol	Chain	Res	Type	Atoms	Z	Observed(^{o})	Ideal(^{o})
6	F	301	DLX	C20-C19-C18	-5.56	109.69	121.11
6	C	301	DLX	C20-C19-C18	-5.56	109.70	121.11
6	E	504	DLX	C20-C19-C18	-5.55	109.71	121.11
6	E	504	DLX	C16-C15-C14	-5.55	109.71	121.11
6	B	504	DLX	C16-C15-C14	-5.55	109.72	121.11
6	B	504	DLX	C47-C15-C14	-5.37	109.72	123.68
6	E	504	DLX	C47-C15-C14	-5.36	109.75	123.68
6	F	301	DLX	C47-C15-C14	-5.35	109.77	123.68
6	C	301	DLX	C47-C15-C14	-5.34	109.81	123.68
6	B	504	DLX	C32-C31-C30	-5.19	110.44	121.11
6	E	504	DLX	C32-C31-C30	-5.19	110.44	121.11
6	C	301	DLX	C45-C23-C22	-5.01	110.67	123.68
6	F	301	DLX	C45-C23-C22	-5.00	110.67	123.68
6	F	301	DLX	C32-C31-C30	-4.94	110.95	121.11
6	C	301	DLX	C32-C31-C30	-4.94	110.97	121.11
6	B	504	DLX	C45-C23-C22	-4.88	111.00	123.68
6	E	504	DLX	C43-C31-C30	-4.87	111.01	123.68
6	E	504	DLX	C45-C23-C22	-4.87	111.01	123.68
6	B	504	DLX	C43-C31-C30	-4.87	111.01	123.68
6	B	504	DLX	C28-C27-C26	-4.87	111.11	121.11
6	E	504	DLX	C28-C27-C26	-4.86	111.12	121.11
6	E	504	DLX	C44-C27-C26	-4.86	111.05	123.68
6	B	504	DLX	C44-C27-C26	-4.85	111.07	123.68
6	B	504	DLX	C24-C23-C22	-4.82	111.22	121.11
6	E	504	DLX	C24-C23-C22	-4.80	111.24	121.11
6	F	301	DLX	C43-C31-C30	-4.77	111.28	123.68
6	C	301	DLX	C43-C31-C30	-4.77	111.29	123.68
6	F	301	DLX	C24-C23-C22	-4.61	111.63	121.11
6	B	503	DLX	C13-C14-C15	-4.61	119.12	126.79
6	C	301	DLX	C24-C23-C22	-4.60	111.65	121.11
6	E	503	DLX	C13-C14-C15	-4.60	119.14	126.79
6	C	301	DLX	C44-C27-C26	-4.54	111.88	123.68
6	F	301	DLX	C44-C27-C26	-4.54	111.89	123.68
6	C	301	DLX	C28-C27-C26	-4.47	111.93	121.11
6	F	301	DLX	C28-C27-C26	-4.46	111.95	121.11
6	E	503	DLX	C13-C05-C04	4.06	120.17	116.88
6	B	503	DLX	C13-C05-C04	4.00	120.13	116.88
7	B	505	PGV	O01-C1-C2	3.97	120.17	111.51
7	E	505	PGV	O01-C1-C2	3.94	120.10	111.51
7	B	506	PGV	O01-C1-C2	3.91	120.05	111.51
6	B	504	DLX	C13-C05-C04	3.80	119.96	116.88
6	F	301	DLX	C13-C05-C04	3.75	119.93	116.88

Continued on next page...

CONFIDENTIAL VALIDATION REPORT

CONFIDENTIAL VALIDATION REPORT

Continued from previous page...

Mol	Chain	Res	Type	Atoms	Z	Observed(^{o})	Ideal(^{o})
6	E	504	DLX	C13-C05-C04	3.74	119.92	116.88
6	C	301	DLX	C13-C05-C04	3.71	119.89	116.88
6	E	503	DLX	C17-C18-C19	-3.67	118.67	127.67
6	B	503	DLX	C17-C18-C19	-3.65	118.70	127.67
6	F	301	DLX	C46-C19-C20	-3.56	109.15	115.29
6	B	504	DLX	C47-C15-C16	-3.54	109.18	115.29
6	C	301	DLX	C46-C19-C20	-3.54	109.18	115.29
6	F	301	DLX	C47-C15-C16	-3.54	109.18	115.29
6	C	301	DLX	C47-C15-C16	-3.54	109.19	115.29
6	E	504	DLX	C47-C15-C16	-3.53	109.21	115.29
6	E	504	DLX	C46-C19-C20	-3.47	109.31	115.29
6	B	504	DLX	C46-C19-C20	-3.46	109.33	115.29
6	B	503	DLX	C21-C22-C23	-2.99	120.33	127.67
6	E	503	DLX	C21-C22-C23	-2.99	120.33	127.67
8	C	302	HEC	CMC-C2C-C1C	-2.95	123.93	128.46
8	F	302	HEC	CMC-C2C-C1C	-2.94	123.95	128.46
6	E	503	DLX	C13-C05-C06	-2.91	119.47	123.30
6	B	503	DLX	C47-C15-C16	2.89	120.26	115.29
6	E	503	DLX	C47-C15-C16	2.89	120.26	115.29
6	E	503	DLX	C45-C23-C24	2.88	120.25	115.29
6	B	503	DLX	C13-C05-C06	-2.87	119.53	123.30
6	B	503	DLX	C45-C23-C24	2.87	120.22	115.29
6	B	503	DLX	C44-C27-C28	2.79	120.09	115.29
6	E	503	DLX	C44-C27-C28	2.77	120.06	115.29
6	E	503	DLX	C46-C19-C20	2.73	119.99	115.29
6	E	504	DLX	C44-C27-C28	-2.73	110.59	115.29
6	B	503	DLX	C46-C19-C20	2.71	119.95	115.29
6	B	504	DLX	C44-C27-C28	-2.71	110.62	115.29
8	F	302	HEC	CAD-CBD-CGD	-2.66	108.12	112.66
6	B	504	DLX	C03-C04-C05	2.65	120.13	116.59
8	C	302	HEC	CAD-CBD-CGD	-2.64	108.15	112.66
6	E	504	DLX	C03-C04-C05	2.62	120.09	116.59
7	B	505	PGV	O03-C19-C20	2.60	120.29	111.93
7	E	505	PGV	O03-C19-C20	2.60	120.28	111.93
6	E	503	DLX	C29-C30-C31	-2.59	121.31	127.67
6	B	503	DLX	C29-C30-C31	-2.58	121.34	127.67
6	B	503	DLX	C25-C26-C27	-2.58	121.34	127.67
6	F	301	DLX	C03-C04-C05	2.57	120.02	116.59
6	E	503	DLX	C25-C26-C27	-2.56	121.37	127.67
6	C	301	DLX	C03-C04-C05	2.54	119.98	116.59
6	E	503	DLX	C03-C04-C05	2.53	119.98	116.59
6	B	503	DLX	C03-C04-C05	2.53	119.97	116.59

Continued on next page...

Continued from previous page...

Mol	Chain	Res	Type	Atoms	Z	Observed(^{o})	Ideal(^{o})
6	B	504	DLX	C43-C31-C32	-2.52	110.94	115.29
7	B	506	PGV	O03-C19-C20	2.50	119.97	111.93
6	E	504	DLX	C43-C31-C32	-2.49	111.00	115.29
6	E	503	DLX	C43-C31-C32	2.46	119.52	115.29
6	F	301	DLX	C13-C05-C06	-2.41	120.13	123.30
6	B	503	DLX	C43-C31-C32	2.41	119.44	115.29
6	C	301	DLX	C13-C05-C06	-2.40	120.15	123.30
6	F	301	DLX	C44-C27-C28	-2.37	111.20	115.29
6	C	301	DLX	C44-C27-C28	-2.37	111.20	115.29
6	E	504	DLX	C13-C05-C06	-2.35	120.21	123.30
5	B	502	HEM	CMC-C2C-C3C	2.34	129.20	124.80
5	E	502	HEM	CMC-C2C-C3C	2.34	129.19	124.80
6	B	504	DLX	C13-C05-C06	-2.33	120.24	123.30
8	F	302	HEC	CMB-C2B-C1B	-2.31	124.92	128.46
8	C	302	HEC	CMB-C2B-C1B	-2.31	124.92	128.46
5	B	502	HEM	CAD-CBD-CGD	-2.29	108.75	112.66
6	F	301	DLX	O12-C04-C05	-2.27	119.49	121.96
6	C	301	DLX	C43-C31-C32	-2.27	111.38	115.29
5	B	502	HEM	CMB-C2B-C3B	2.27	129.05	124.80
5	E	502	HEM	CAD-CBD-CGD	-2.27	108.79	112.66
6	F	301	DLX	C43-C31-C32	-2.24	111.42	115.29
6	C	301	DLX	O12-C04-C05	-2.23	119.53	121.96
5	E	502	HEM	CMB-C2B-C3B	2.22	128.96	124.80
8	C	302	HEC	C1D-C2D-C3D	-2.21	105.46	107.00
5	B	502	HEM	CMA-C3A-C4A	-2.18	125.11	128.46
8	F	302	HEC	C1D-C2D-C3D	-2.17	105.48	107.00
5	E	502	HEM	CMA-C3A-C4A	-2.15	125.16	128.46
7	B	506	PGV	C02-O01-C1	-2.05	112.70	117.82
5	B	501	HEM	CBD-CAD-C3D	-2.04	108.58	112.47
5	E	501	HEM	CBD-CAD-C3D	-2.02	108.62	112.47
6	B	503	DLX	O12-C04-C05	-2.01	119.77	121.96
6	E	503	DLX	O12-C04-C05	-2.00	119.78	121.96

There are no chirality outliers.

All (190) torsion outliers are listed below:

Mol	Chain	Res	Type	Atoms
6	F	301	DLX	C29-C30-C31-C43
6	F	301	DLX	C25-C26-C27-C44
6	F	301	DLX	C45-C23-C24-C25
6	F	301	DLX	C21-C22-C23-C45
6	F	301	DLX	C21-C22-C23-C24

Continued on next page...

CONFIDENTIAL VALIDATION REPORT

Continued from previous page...

Mol	Chain	Res	Type	Atoms
6	F	301	DLX	C17-C18-C19-C46
6	F	301	DLX	C17-C18-C19-C20
6	F	301	DLX	C13-C14-C15-C47
8	C	302	HEC	C1A-C2A-CAA-CBA
5	E	501	HEM	C3D-CAD-CBD-CGD
6	B	503	DLX	C45-C23-C24-C25
6	B	503	DLX	C22-C23-C24-C25
6	B	504	DLX	C29-C30-C31-C32
6	B	504	DLX	C25-C26-C27-C44
6	B	504	DLX	C45-C23-C24-C25
6	B	504	DLX	C21-C22-C23-C45
6	B	504	DLX	C21-C22-C23-C24
6	B	504	DLX	C17-C18-C19-C46
6	B	504	DLX	C13-C14-C15-C47
7	B	506	PGV	C03-O11-P-O13
7	B	506	PGV	C03-O11-P-O14
7	B	506	PGV	C04-O12-P-O13
7	B	506	PGV	C2-C1-O01-C02
7	E	505	PGV	C04-O12-P-O14
7	E	505	PGV	O03-C01-C02-O01
7	E	505	PGV	C04-C05-C06-O06
7	B	505	PGV	C04-O12-P-O14
7	B	505	PGV	O03-C01-C02-O01
7	B	505	PGV	C04-C05-C06-O06
8	F	302	HEC	C1A-C2A-CAA-CBA
5	B	501	HEM	C3D-CAD-CBD-CGD
6	C	301	DLX	C29-C30-C31-C43
6	C	301	DLX	C25-C26-C27-C44
6	C	301	DLX	C45-C23-C24-C25
6	C	301	DLX	C21-C22-C23-C45
6	C	301	DLX	C21-C22-C23-C24
6	C	301	DLX	C17-C18-C19-C46
6	C	301	DLX	C17-C18-C19-C20
6	C	301	DLX	C13-C14-C15-C47
6	E	503	DLX	C45-C23-C24-C25
6	E	503	DLX	C22-C23-C24-C25
6	E	504	DLX	C29-C30-C31-C32
6	E	504	DLX	C25-C26-C27-C44
6	E	504	DLX	C45-C23-C24-C25
6	E	504	DLX	C21-C22-C23-C45
6	E	504	DLX	C21-C22-C23-C24
6	E	504	DLX	C17-C18-C19-C46

Continued on next page...

CONFIDENTIAL VALIDATION REPORT

Continued from previous page...

Mol	Chain	Res	Type	Atoms
6	E	504	DLX	C13-C14-C15-C47
7	B	506	PGV	O02-C1-O01-C02
6	B	504	DLX	C43-C31-C32-C33
6	E	504	DLX	C43-C31-C32-C33
6	B	504	DLX	C25-C26-C27-C28
6	B	504	DLX	C17-C18-C19-C20
6	E	504	DLX	C25-C26-C27-C28
6	E	504	DLX	C17-C18-C19-C20
6	B	503	DLX	C31-C32-C33-C34
6	E	503	DLX	C31-C32-C33-C34
6	F	301	DLX	C23-C24-C25-C26
6	F	301	DLX	C15-C16-C17-C18
6	B	504	DLX	C19-C20-C21-C22
6	B	504	DLX	C15-C16-C17-C18
6	C	301	DLX	C23-C24-C25-C26
6	C	301	DLX	C15-C16-C17-C18
6	E	504	DLX	C19-C20-C21-C22
6	E	504	DLX	C15-C16-C17-C18
6	F	301	DLX	C25-C26-C27-C28
6	C	301	DLX	C25-C26-C27-C28
7	B	506	PGV	O05-C05-C06-O06
6	B	503	DLX	C19-C20-C21-C22
6	E	503	DLX	C19-C20-C21-C22
6	F	301	DLX	C36-C37-C38-C39
6	C	301	DLX	C36-C37-C38-C39
7	B	506	PGV	C04-O12-P-O11
7	E	505	PGV	C04-O12-P-O11
7	B	505	PGV	C04-O12-P-O11
7	B	506	PGV	C2-C3-C4-C5
7	B	506	PGV	C5-C6-C7-C8
7	B	506	PGV	C29-C30-C31-C32
7	B	506	PGV	C04-C05-C06-O06
6	F	301	DLX	C47-C15-C16-C17
6	C	301	DLX	C47-C15-C16-C17
7	B	506	PGV	C24-C25-C26-C27
7	B	506	PGV	C23-C24-C25-C26
7	B	506	PGV	C6-C7-C8-C9
7	E	505	PGV	C28-C29-C30-C31
7	B	506	PGV	C27-C28-C29-C30
7	B	505	PGV	C28-C29-C30-C31
6	B	503	DLX	C37-C38-C39-C40
6	E	503	DLX	C37-C38-C39-C40

Continued on next page...

CONFIDENTIAL VALIDATION REPORT

Continued from previous page...

Mol	Chain	Res	Type	Atoms
7	B	506	PGV	C7-C8-C9-C10
7	B	506	PGV	C1-C2-C3-C4
6	F	301	DLX	C26-C27-C28-C29
6	F	301	DLX	C14-C15-C16-C17
6	C	301	DLX	C26-C27-C28-C29
6	C	301	DLX	C14-C15-C16-C17
6	B	503	DLX	C37-C38-C39-C41
6	E	503	DLX	C37-C38-C39-C41
6	F	301	DLX	C13-C14-C15-C16
6	C	301	DLX	C13-C14-C15-C16
7	B	506	PGV	C14-C15-C16-C17
7	E	505	PGV	C25-C26-C27-C28
7	B	505	PGV	C25-C26-C27-C28
7	B	506	PGV	C19-C20-C21-C22
6	F	301	DLX	C43-C31-C32-C33
6	C	301	DLX	C43-C31-C32-C33
6	F	301	DLX	C33-C34-C35-C42
6	C	301	DLX	C33-C34-C35-C42
7	B	506	PGV	C03-O11-P-O12
6	F	301	DLX	C04-C05-C13-C14
6	B	503	DLX	C04-C05-C13-C14
6	C	301	DLX	C04-C05-C13-C14
6	E	503	DLX	C04-C05-C13-C14
7	B	506	PGV	O03-C01-C02-C03
7	E	505	PGV	O03-C01-C02-C03
7	B	505	PGV	O03-C01-C02-C03
6	B	504	DLX	C05-C13-C14-C15
6	E	504	DLX	C05-C13-C14-C15
7	B	506	PGV	C15-C16-C17-C18
6	B	504	DLX	C14-C15-C16-C17
6	E	504	DLX	C14-C15-C16-C17
7	E	505	PGV	O05-C05-C06-O06
7	B	505	PGV	O05-C05-C06-O06
7	E	505	PGV	C21-C22-C23-C24
7	B	505	PGV	C21-C22-C23-C24
7	B	506	PGV	C31-C32-C33-C34
6	F	301	DLX	C29-C30-C31-C32
6	C	301	DLX	C29-C30-C31-C32
7	E	505	PGV	C20-C19-O03-C01
7	B	505	PGV	C20-C19-O03-C01
6	F	301	DLX	C33-C34-C35-C36
6	B	504	DLX	C33-C34-C35-C36

Continued on next page...

CONFIDENTIAL VALIDATION REPORT

Continued from previous page...

Mol	Chain	Res	Type	Atoms
6	C	301	DLX	C33-C34-C35-C36
6	E	504	DLX	C33-C34-C35-C36
7	B	506	PGV	C28-C29-C30-C31
7	B	506	PGV	C01-C02-C03-O11
7	E	505	PGV	C19-C20-C21-C22
7	B	505	PGV	C19-C20-C21-C22
7	B	506	PGV	C26-C27-C28-C29
7	E	505	PGV	C26-C27-C28-C29
7	B	505	PGV	C26-C27-C28-C29
7	B	506	PGV	O01-C02-C03-O11
7	E	505	PGV	C11-C10-C9-C8
7	B	505	PGV	C11-C10-C9-C8
7	E	505	PGV	O04-C19-O03-C01
7	B	505	PGV	O04-C19-O03-C01
7	B	506	PGV	C13-C14-C15-C16
6	B	503	DLX	C36-C37-C38-C39
6	E	503	DLX	C36-C37-C38-C39
6	B	503	DLX	C06-C05-C13-C14
6	E	503	DLX	C06-C05-C13-C14
7	B	506	PGV	O03-C01-C02-O01
6	B	504	DLX	C42-C35-C36-C37
6	E	504	DLX	C42-C35-C36-C37
7	B	506	PGV	C04-O12-P-O14
7	E	505	PGV	C04-O12-P-O13
7	B	505	PGV	C04-O12-P-O13
8	C	302	HEC	C3A-C2A-CAA-CBA
8	F	302	HEC	C3A-C2A-CAA-CBA
6	B	504	DLX	C33-C34-C35-C42
6	E	504	DLX	C33-C34-C35-C42
6	B	504	DLX	C44-C27-C28-C29
6	E	504	DLX	C44-C27-C28-C29
7	B	506	PGV	C12-C13-C14-C15
7	B	506	PGV	O12-C04-C05-C06
6	B	504	DLX	C27-C28-C29-C30
6	E	504	DLX	C27-C28-C29-C30
7	B	506	PGV	C21-C22-C23-C24
7	B	506	PGV	C20-C21-C22-C23
6	F	301	DLX	C06-C05-C13-C14
6	C	301	DLX	C06-C05-C13-C14
6	E	503	DLX	C35-C36-C37-C38
6	B	503	DLX	C35-C36-C37-C38
6	E	504	DLX	C35-C36-C37-C38

Continued on next page...

CONFIDENTIAL VALIDATION REPORT

Continued from previous page...

Mol	Chain	Res	Type	Atoms
6	B	504	DLX	C35-C36-C37-C38
7	E	505	PGV	O03-C19-C20-C21
7	B	505	PGV	O03-C19-C20-C21
7	E	505	PGV	O01-C1-C2-C3
7	B	505	PGV	O01-C1-C2-C3
6	F	301	DLX	C16-C17-C18-C19
6	B	504	DLX	C28-C29-C30-C31
6	C	301	DLX	C16-C17-C18-C19
6	E	504	DLX	C28-C29-C30-C31
7	E	505	PGV	C22-C23-C24-C25
7	B	505	PGV	C22-C23-C24-C25
7	E	505	PGV	O04-C19-C20-C21
7	B	505	PGV	O04-C19-C20-C21
6	B	504	DLX	C32-C33-C34-C35
6	E	504	DLX	C32-C33-C34-C35
7	B	505	PGV	C20-C21-C22-C23
7	E	505	PGV	C20-C21-C22-C23

There are no ring outliers.

14 monomers are involved in 57 short contacts:

Mol	Chain	Res	Type	Clashes	Symm-Clashes
4	A	501	FES	1	0
5	B	501	HEM	4	0
5	B	502	HEM	3	0
6	B	503	DLX	13	0
7	B	505	PGV	1	0
7	B	506	PGV	14	0
8	C	302	HEC	9	0
4	D	501	FES	1	0
5	E	501	HEM	4	0
5	E	502	HEM	3	0
6	E	503	DLX	7	0
6	E	504	DLX	1	0
7	E	505	PGV	1	0
8	F	302	HEC	8	0

The following is a two-dimensional graphical depiction of Mogul quality analysis of bond lengths, bond angles, torsion angles, and ring geometry for all instances of the Ligand of Interest. In addition, ligands with molecular weight > 250 and outliers as shown on the validation Tables will also be included. For torsion angles, if less then 5% of the Mogul distribution of torsion angles is within 10 degrees of the torsion angle in question, then that torsion angle is considered an outlier.

CONFIDENTIAL VALIDATION REPORT

Any bond that is central to one or more torsion angles identified as an outlier by Mogul will be highlighted in the graph. For rings, the root-mean-square deviation (RMSD) between the ring in question and similar rings identified by Mogul is calculated over all ring torsion angles. If the average RMSD is greater than 60 degrees and the minimal RMSD between the ring in question and any Mogul-identified rings is also greater than 60 degrees, then that ring is considered an outlier. The outliers are highlighted in purple. The color gray indicates Mogul did not find sufficient equivalents in the CSD to analyse the geometry.

Ligand HEM B 501

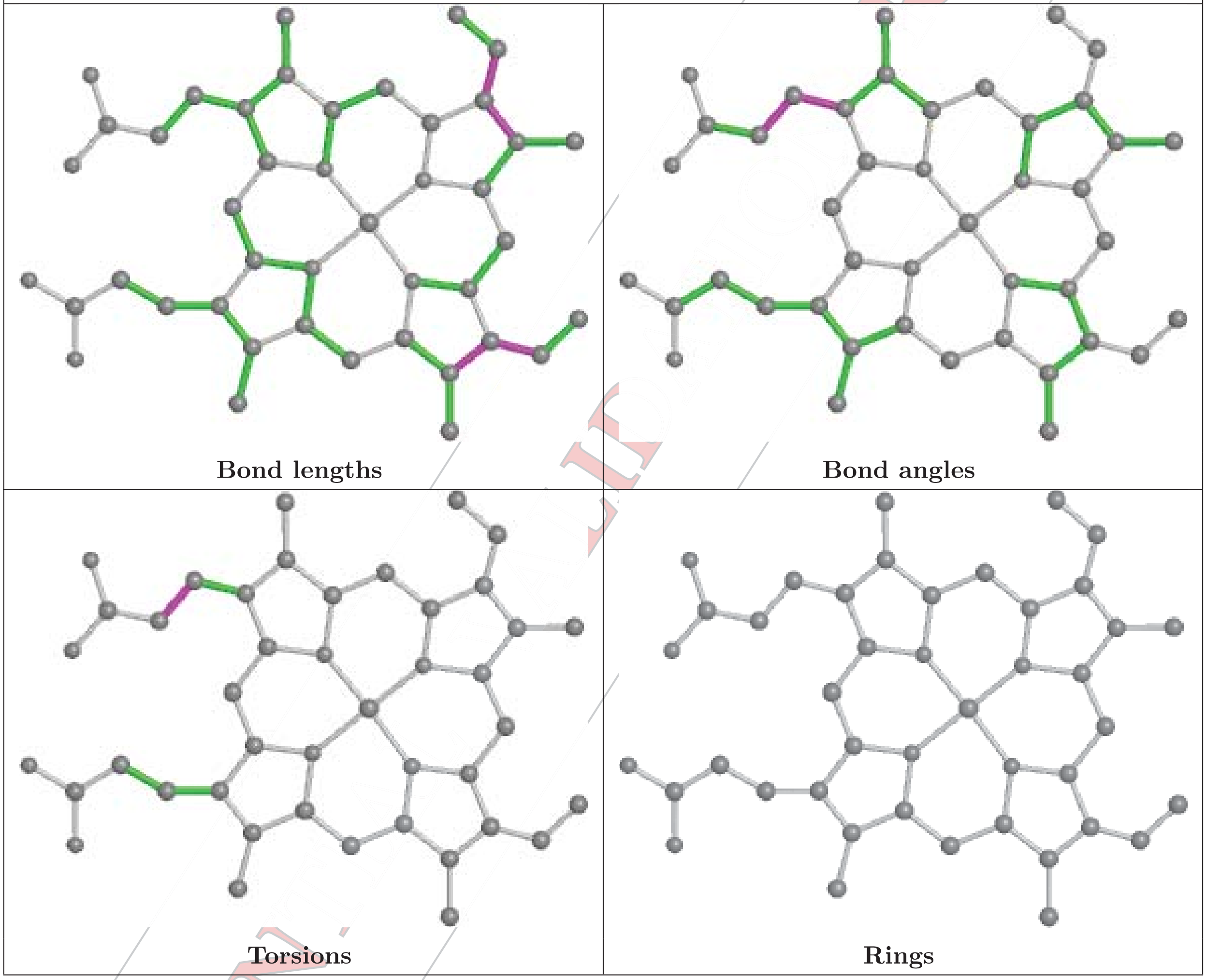

CONFIDENTIAL REPORT

Ligand HEM B 502

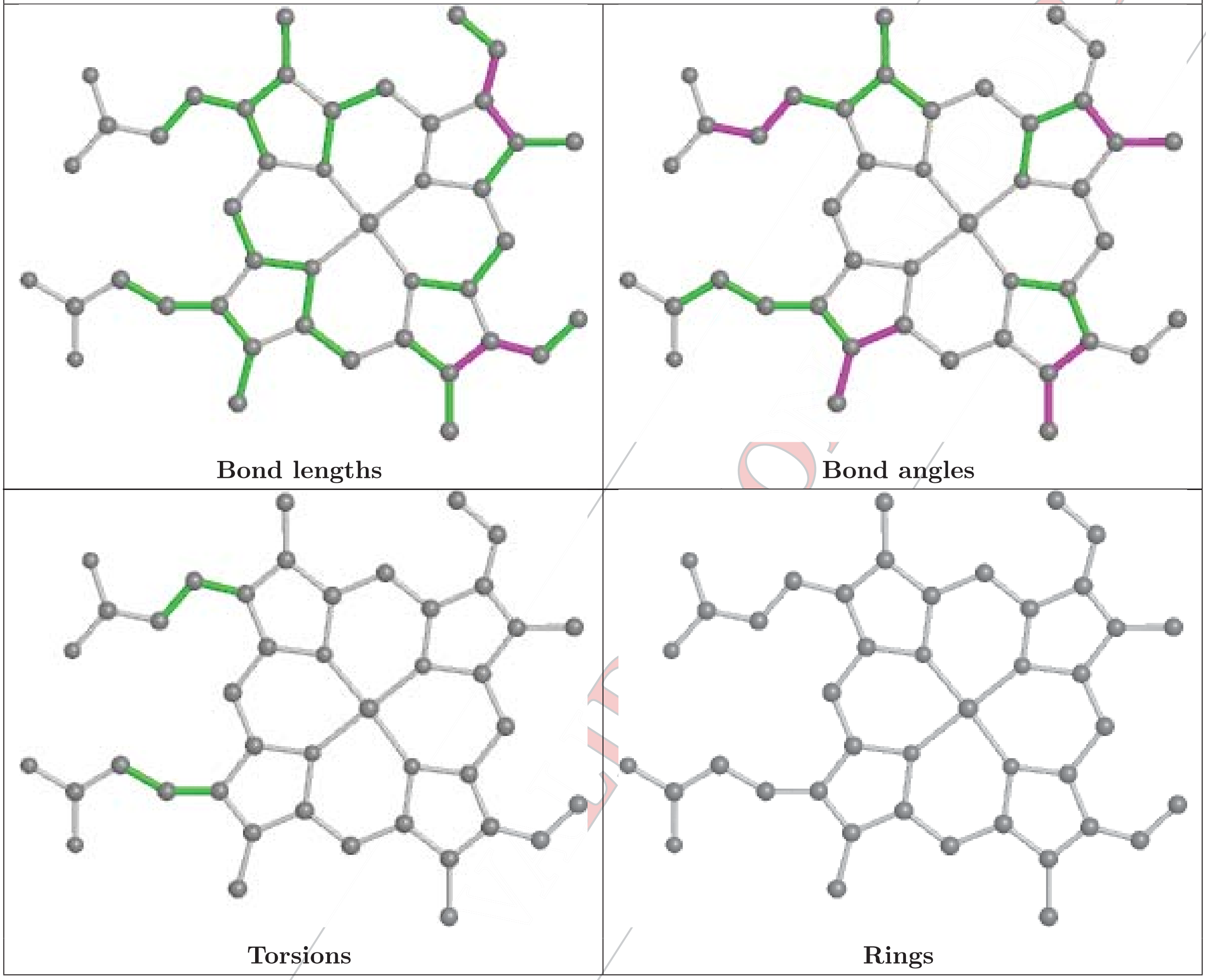

Ligand DLX B 503

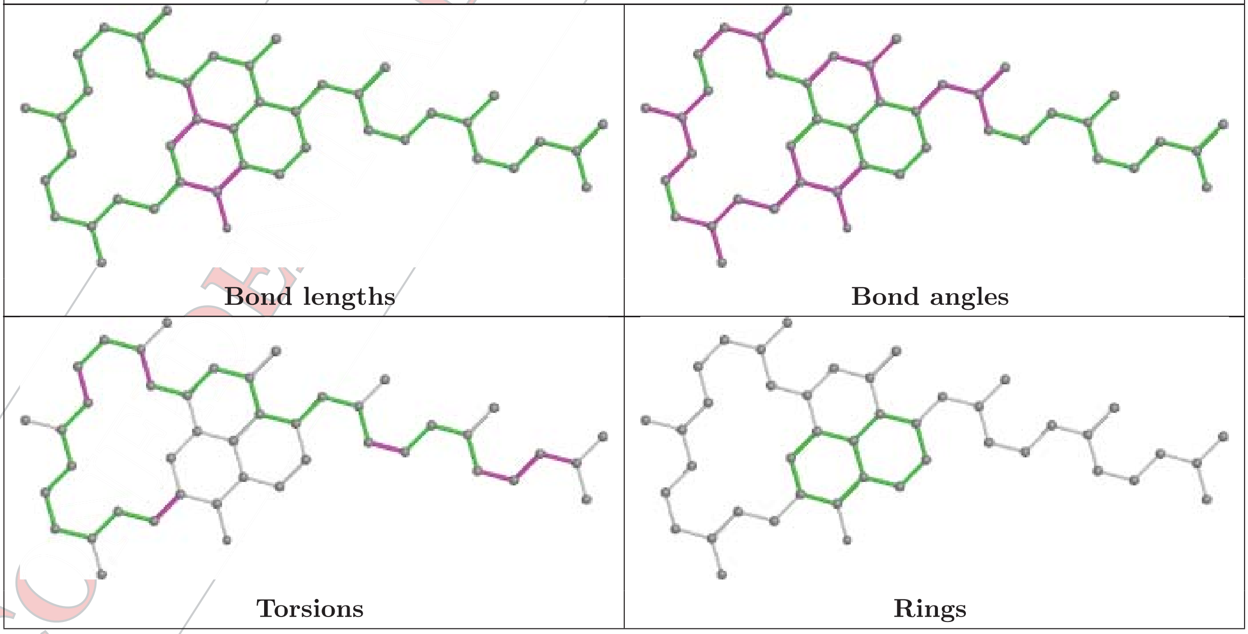

Ligand DLX B 504

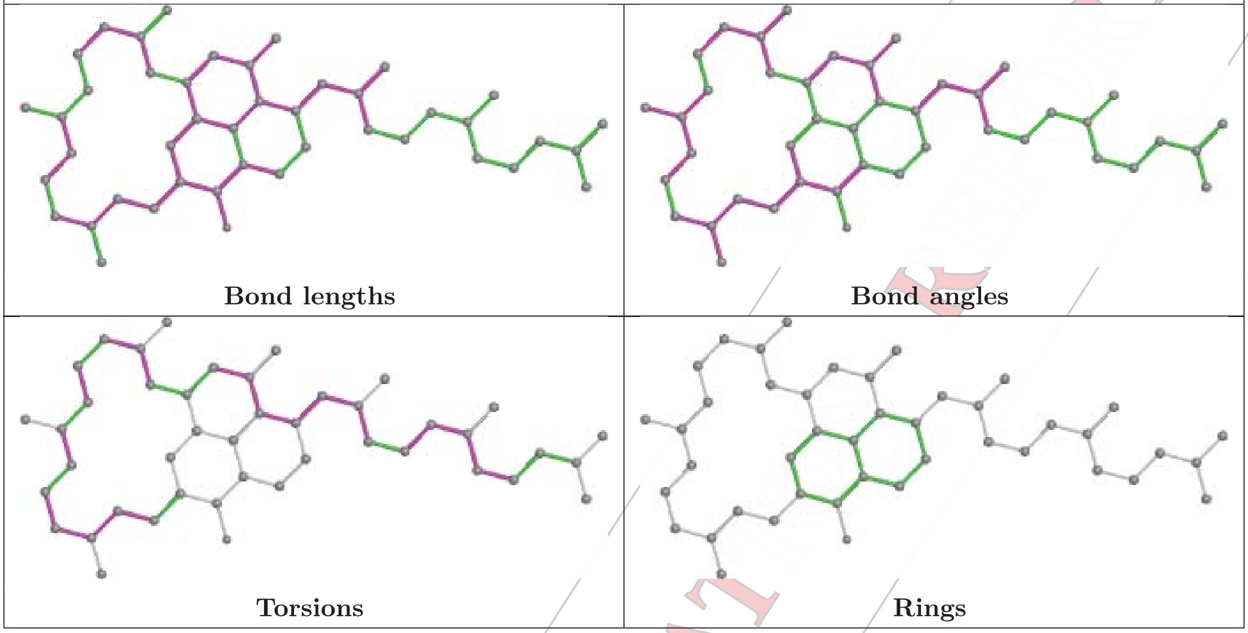

Ligand PGV B 505

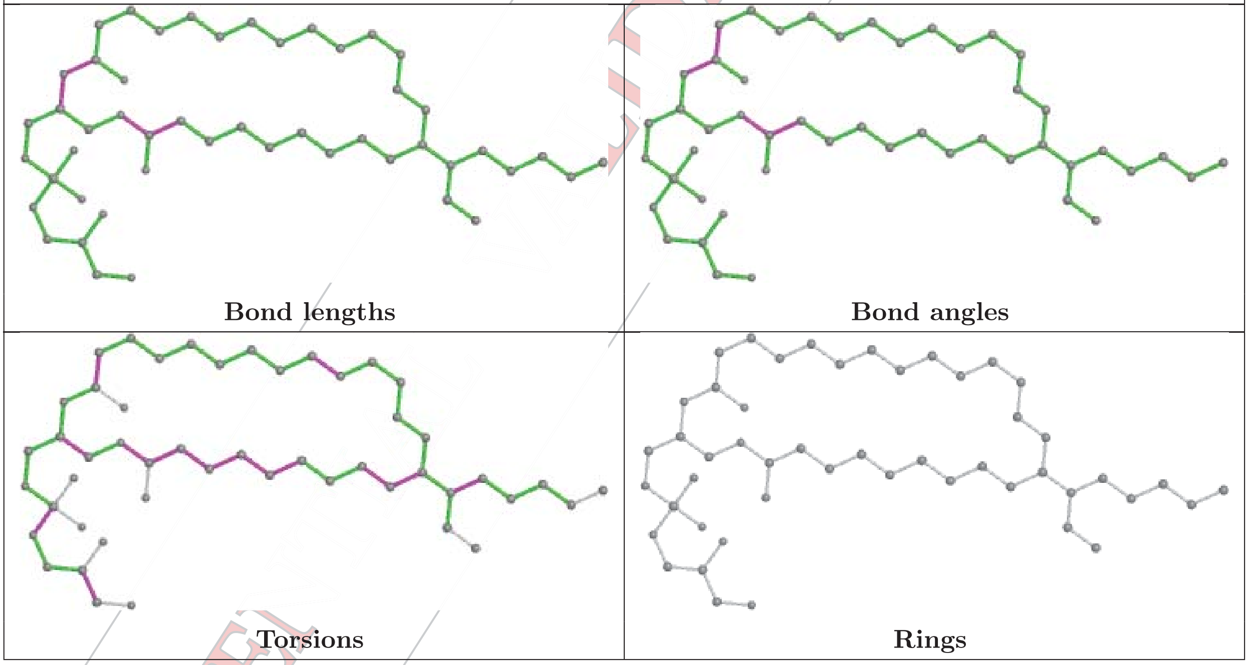

Ligand PGV B 506

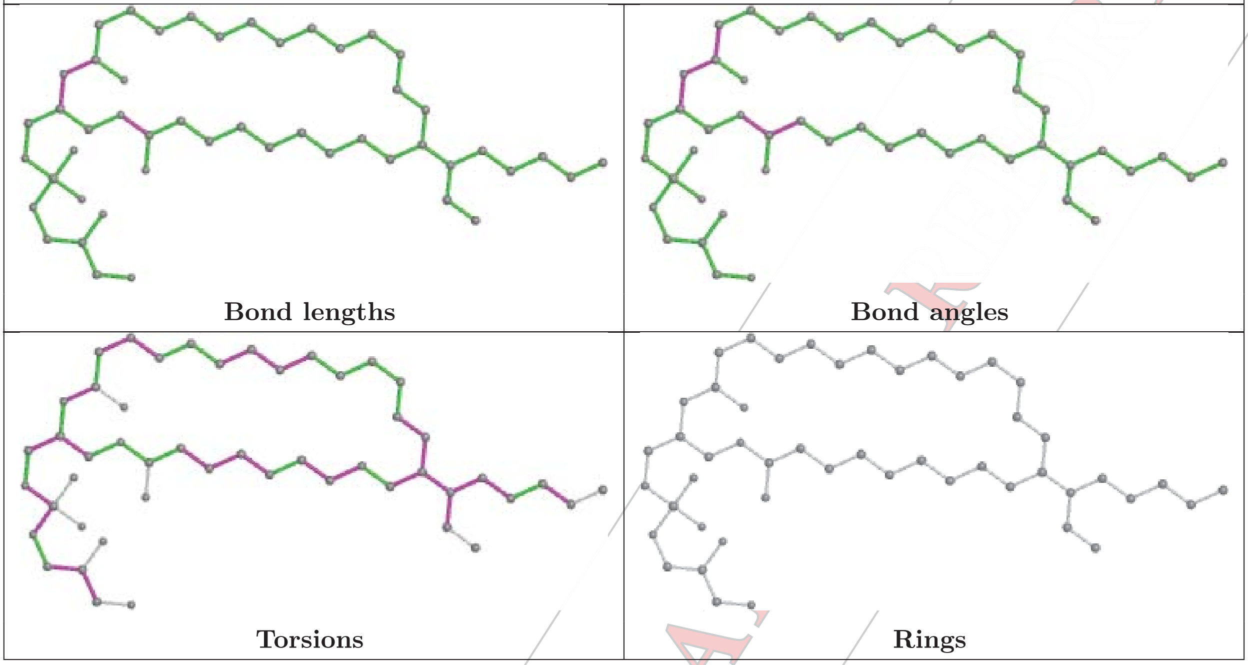

Ligand DLX C 301

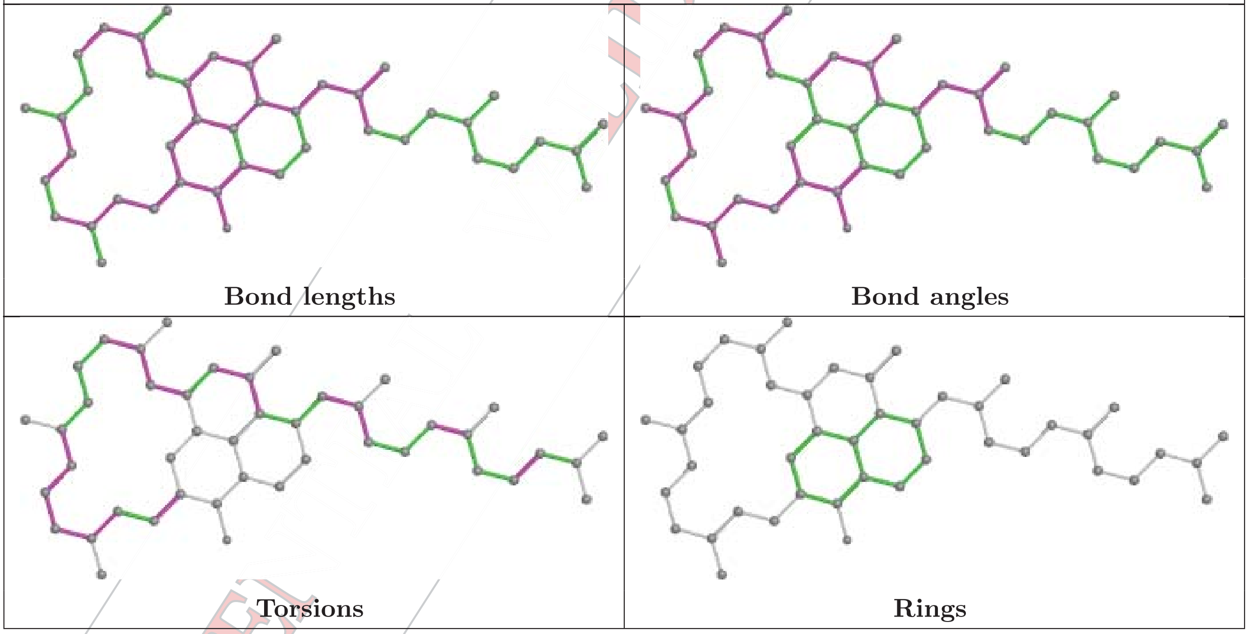

CONFIDENTIAL DRAFT

Ligand HEC C 302

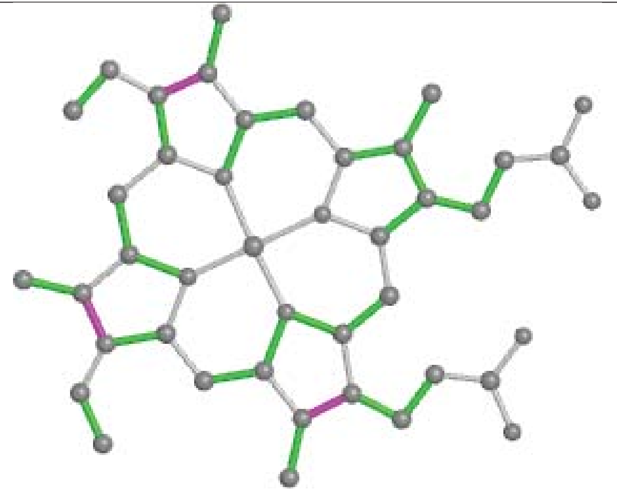

Bond lengths

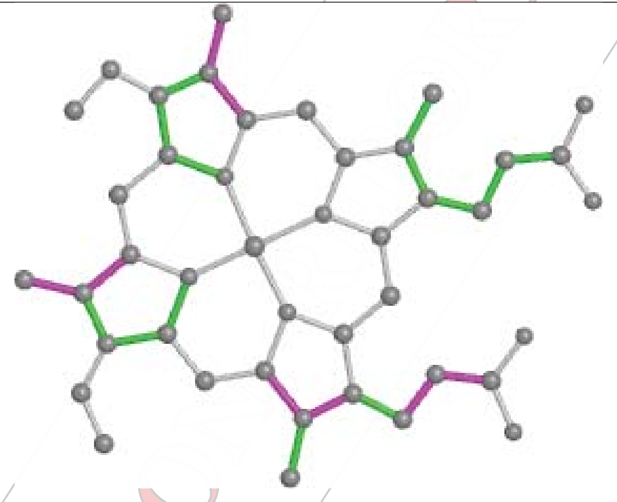

Bond angles

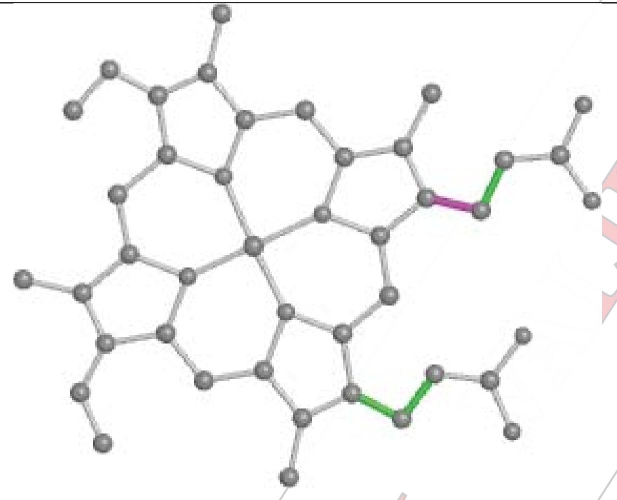

Torsions

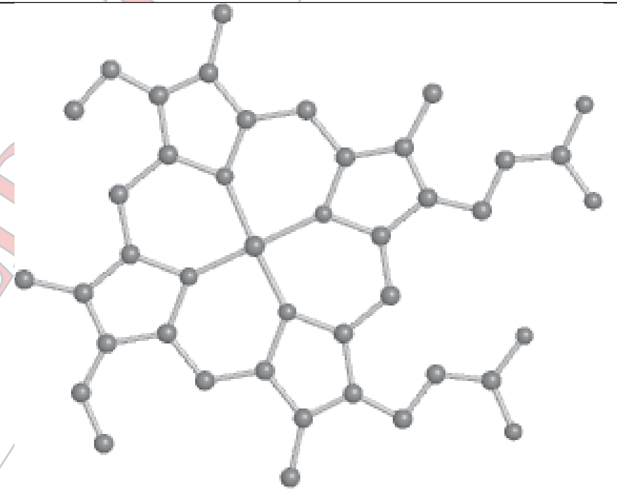

Rings

CONFIDENTIAL

Ligand HEM E 501

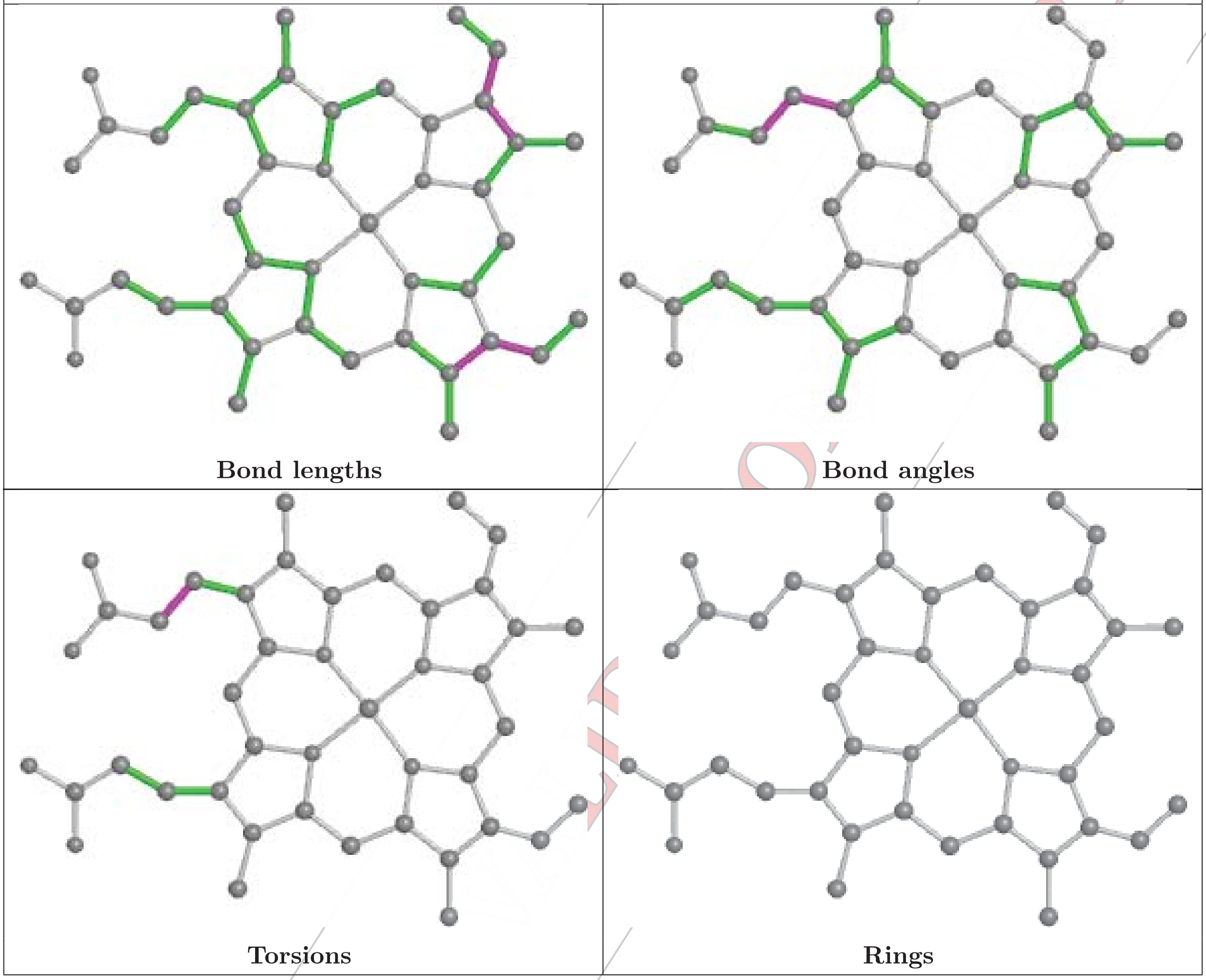

CONFIDENTIAL

Ligand HEM E 502

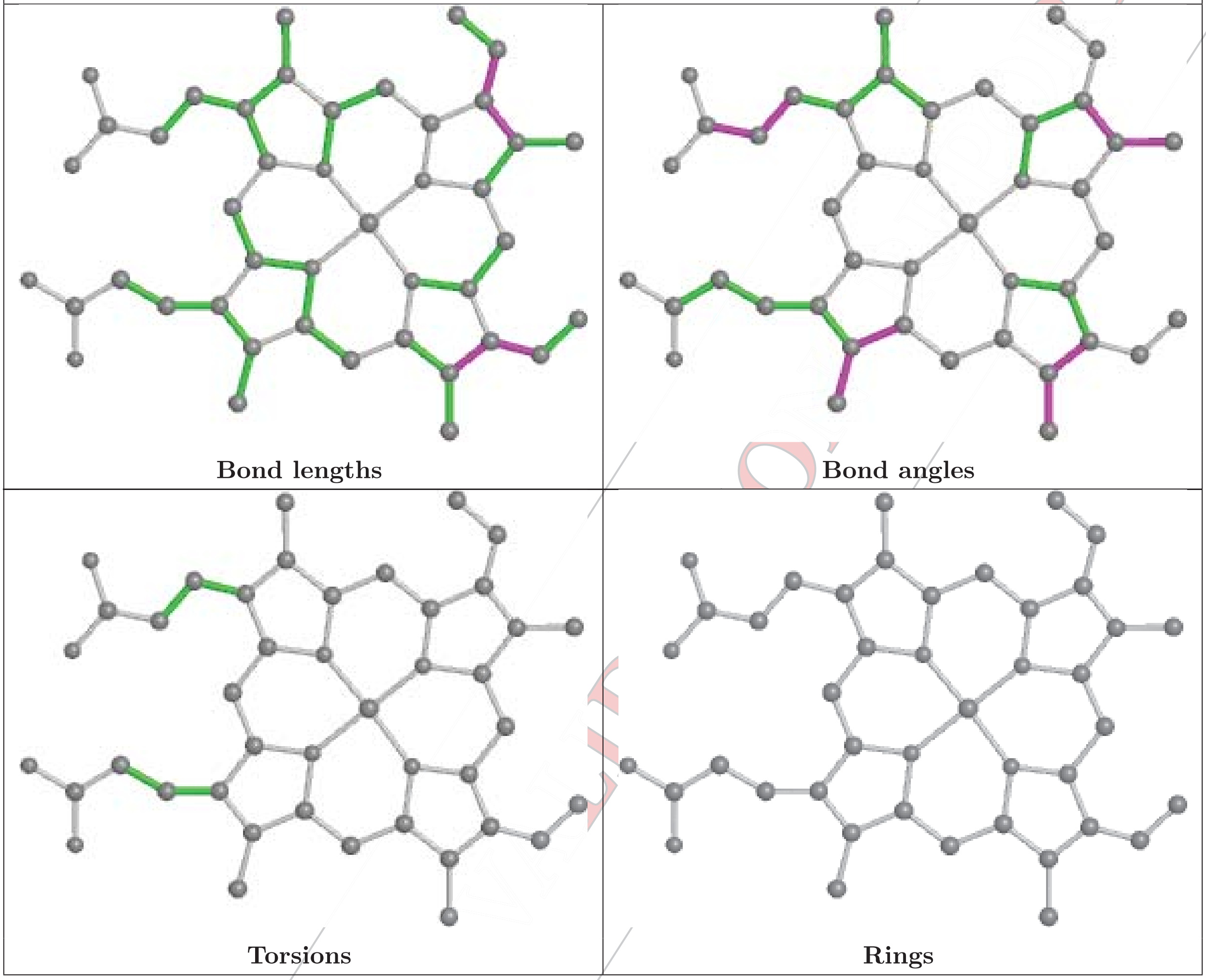

Ligand DLX E 503

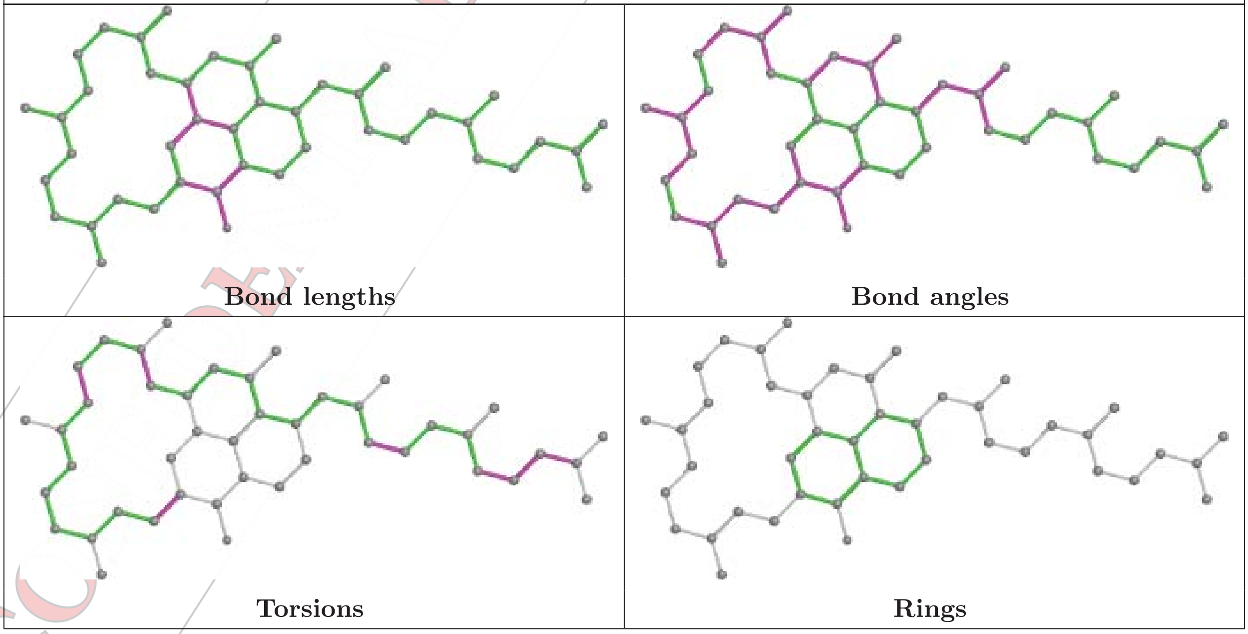

Ligand DLX E 504

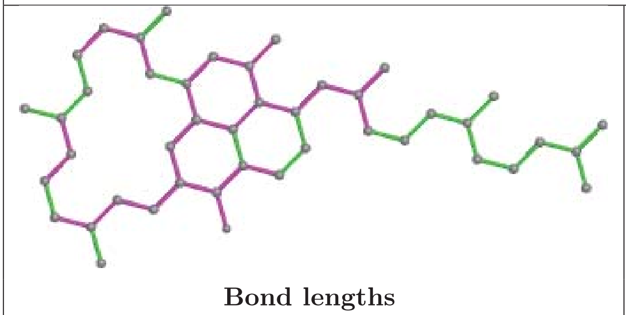

Bond lengths

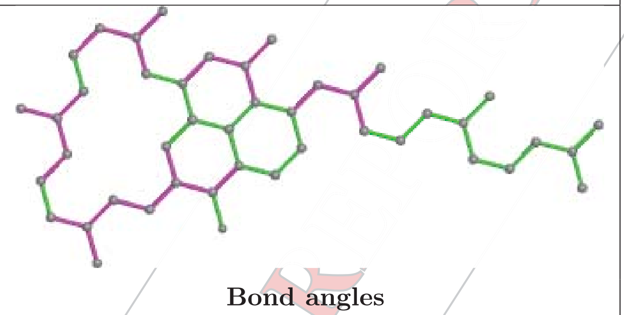

Bond angles

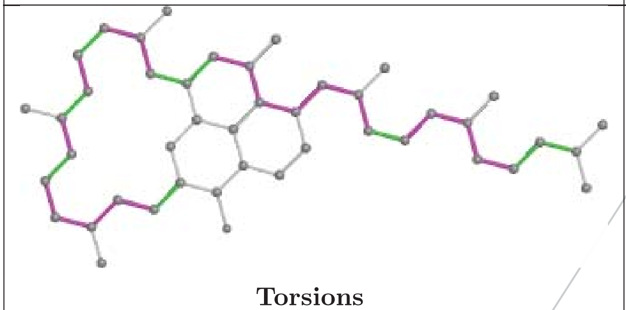

Torsions

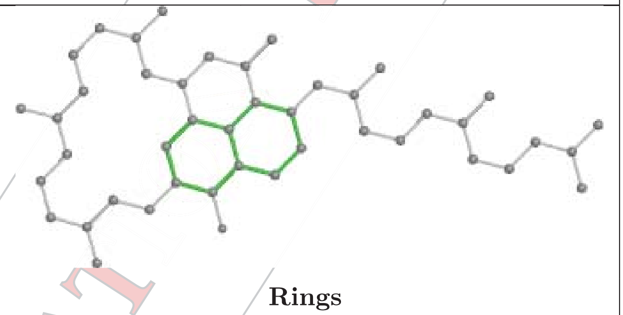

Rings

Ligand PGV E 505

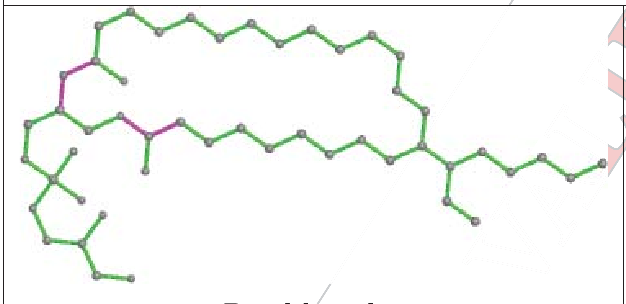

Bond lengths

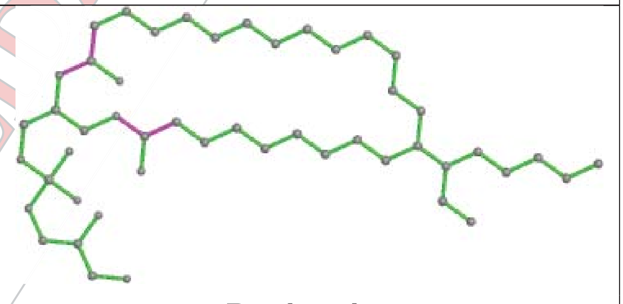

Bond angles

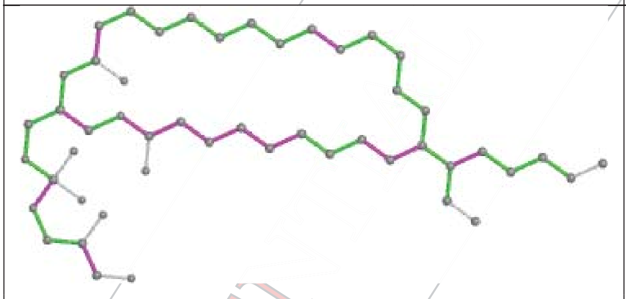

Torsions

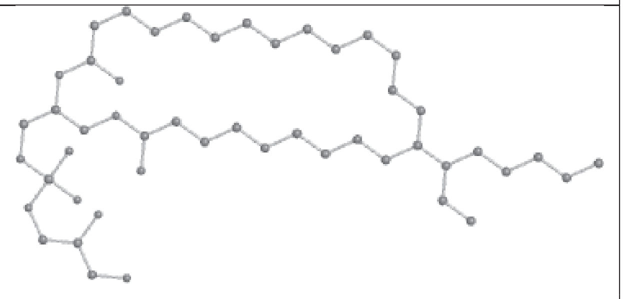

Rings

CONFIDENTIAL VALIDATION REPORT

Ligand DLX F 301	
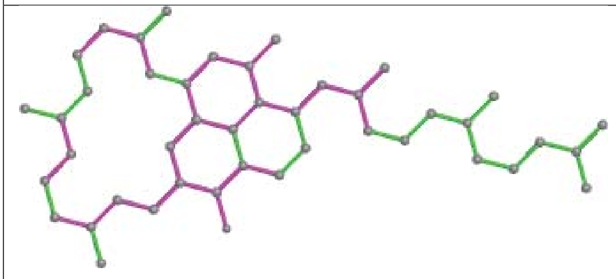 Bond lengths	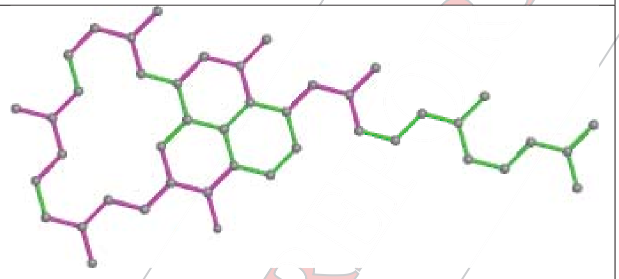 Bond angles
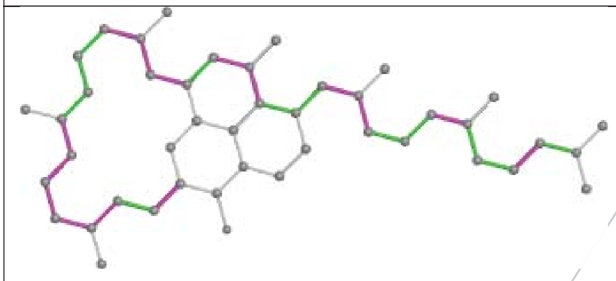 Torsions	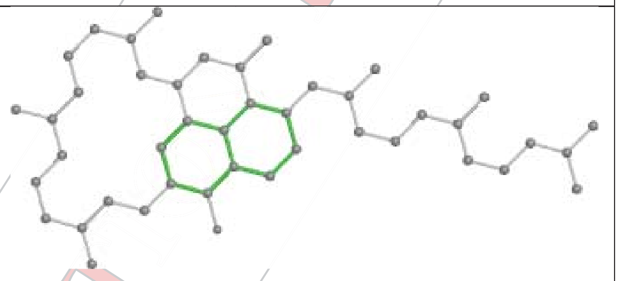 Rings

CONFIDENTIAL VALIDATION REPORT

Ligand HEC F 302

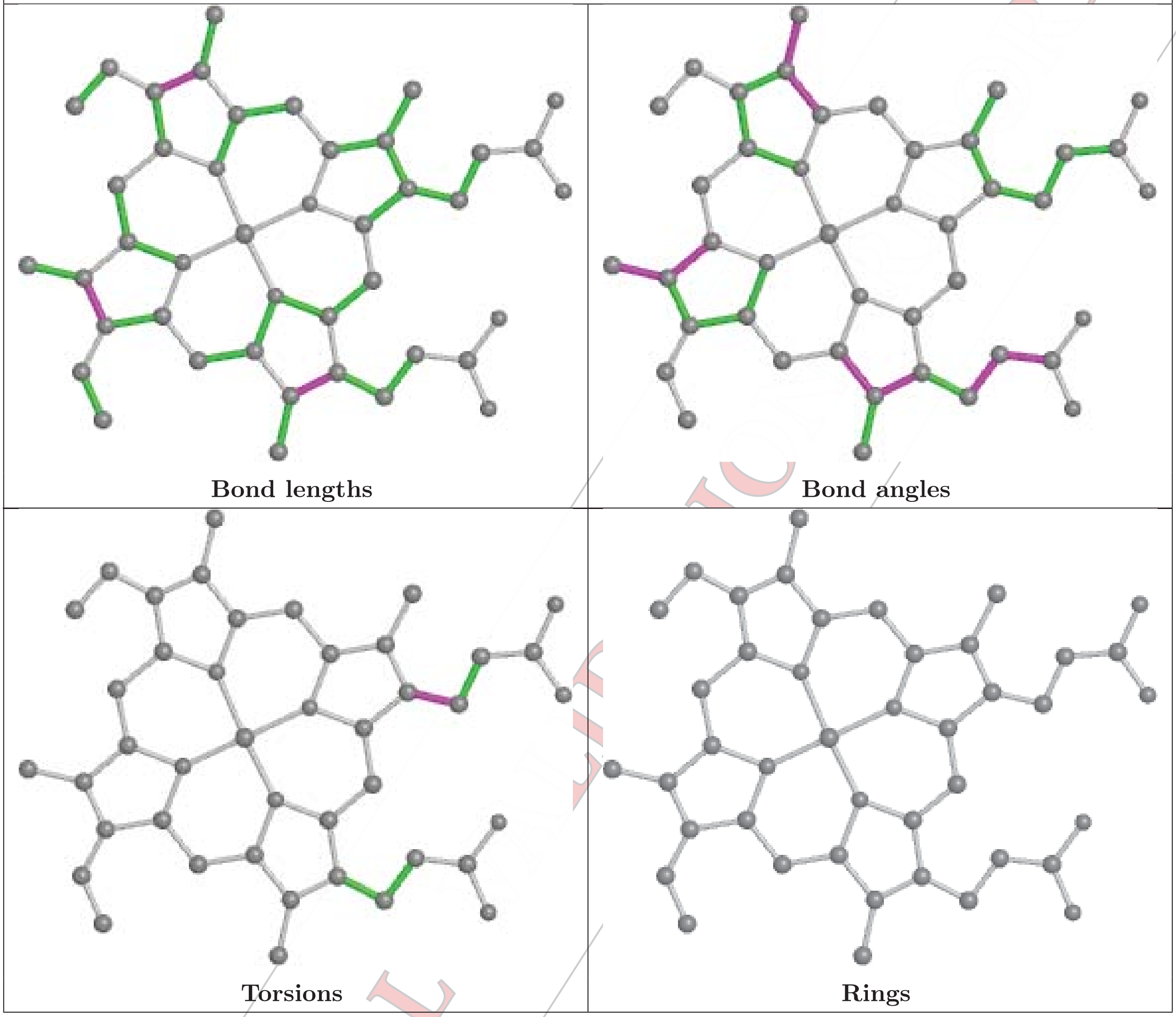

5.7 Other polymers (i)

There are no such residues in this entry.

5.8 Polymer linkage issues (i)

There are no chain breaks in this entry.

CONFIDENTIAL

Full wwPDB/EMDataBank EM Map/Model Validation Report (i)

Aug 14, 2019 – 04:08 PM JST

Deposition ID	:	D_1300013263
PDB ID	:	*(not yet assigned)*

This is a Full wwPDB/EMDataBank EM Map/Model Validation Report.

This report is produced by the wwPDB Deposition System during initial deposition but before annotation of the structure.

We welcome your comments at *validation@mail.wwpdb.org*
A user guide is available at
https://www.wwpdb.org/validation/2017/EMValidationReportHelp
with specific help available everywhere you see the (i) symbol.

MolProbity	:	4.02b-467
Mogul	:	1.8.0 (224370), CSD as540be (2019)
Percentile statistics	:	20171227.v01 (using entries in the PDB archive December 27th 2017)
Ideal geometry (proteins)	:	Engh & Huber (2001)
Ideal geometry (DNA, RNA)	:	Parkinson et. al. (1996)
Validation Pipeline (wwPDB-VP)	:	2.4

PRELIMINARY VALIDATION REPORT

1 Overall quality at a glance (i)

The following experimental techniques were used to determine the structure:
ELECTRON MICROSCOPY

The reported resolution of this entry is unknown.

Percentile scores (ranging between 0-100) for global validation metrics of the entry are shown in the following graphic. The table shows the number of entries on which the scores are based.

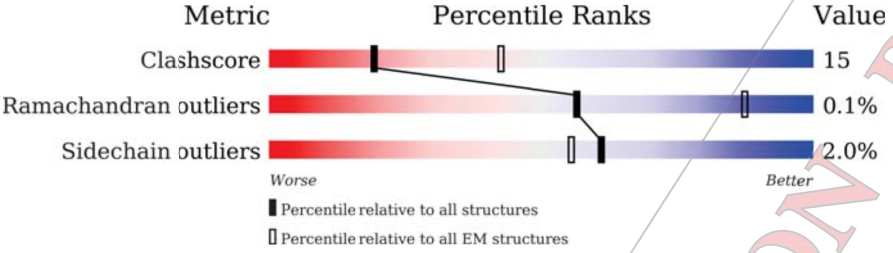

Metric	Whole archive (#Entries)	EM structures (#Entries)
Clashscore	136327	1886
Ramachandran outliers	132723	1663
Sidechain outliers	132532	1531

The table below summarises the geometric issues observed across the polymeric chains. The red, orange, yellow and green segments on the bar indicate the fraction of residues that contain outliers for >=3, 2, 1 and 0 types of geometric quality criteria. A grey segment represents the fraction of residues that are not modelled. The numeric value for each fraction is indicated below the corresponding segment, with a dot representing fractions <=5%

Mol	Chain	Length	Quality of chain
1	A	181	40% 32% • 26%
1	D	181	38% 34% • 26%
2	B	410	76% 20% •
2	E	410	75% 21% •
3	C	240	65% 30% • •
3	F	240	65% 30% • •

The following table lists non-polymeric compounds, carbohydrate monomers and non-standard residues in protein, DNA, RNA chains that are outliers for geometric or electron-density-fit criteria:

PRELIMINARY VALIDATION REPORT

Mol	Type	Chain	Res	Chirality	Geometry	Clashes	Electron density
6	AMY	B	503	-	-	X	-
7	NQA	E	505	-	-	X	-

PRELIMINARY VALIDATION REPORT

2 Entry composition (i)

There are 9 unique types of molecules in this entry. The entry contains 12993 atoms, of which 0 are hydrogens and 0 are deuteriums.

In the tables below, the AltConf column contains the number of residues with at least one atom in alternate conformation and the Trace column contains the number of residues modelled with at most 2 atoms.

- Molecule 1 is a protein called Rieske-I iron sulfur protein.

Mol	Chain	Residues	Atoms					AltConf	Trace
			Total	C	N	O	S		
1	A	134	1012	668	169	169	6	0	0
1	D	134	1012	668	169	169	6	0	0

- Molecule 2 is a protein called cytochrome b subunit.

Mol	Chain	Residues	Atoms					AltConf	Trace
			Total	C	N	O	S		
2	B	395	3226	2208	491	515	12	0	0
2	E	395	3226	2208	491	515	12	0	0

- Molecule 3 is a protein called Cytochrome c.

Mol	Chain	Residues	Atoms					AltConf	Trace
			Total	C	N	O	S		
3	C	236	1917	1263	312	334	8	0	0
3	F	236	1917	1263	312	334	8	0	0

- Molecule 4 is FE2/S2 (INORGANIC) CLUSTER (three-letter code: FES) (formula: Fe_2S_2).

PRELIMINARY VALIDATION REPORT

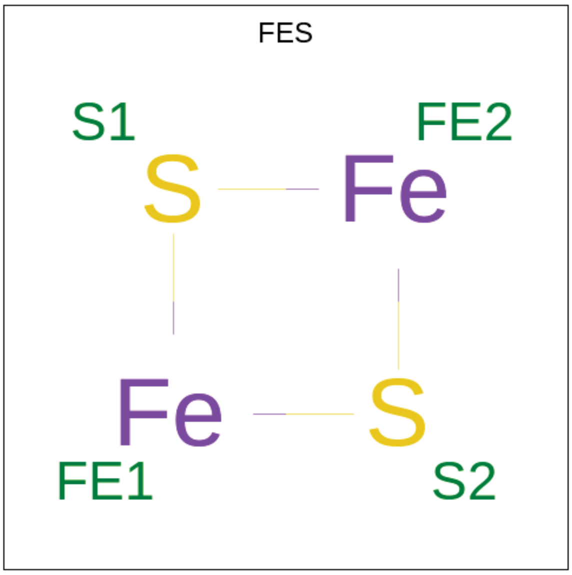

Mol	Chain	Residues	Atoms			AltConf
			Total	Fe	S	
4	A	1	4	2	2	0
			Total	Fe	S	
4	D	1	4	2	2	0

- Molecule 5 is PROTOPORPHYRIN IX CONTAINING FE (three-letter code: HEM) (formula: $C_{34}H_{32}FeN_4O_4$).

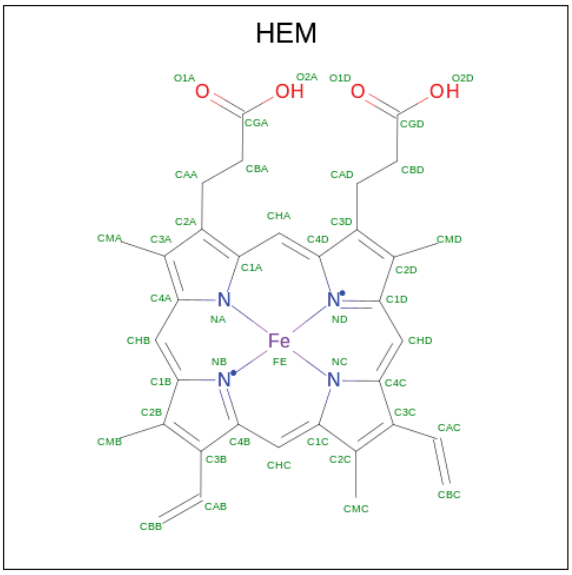

Mol	Chain	Residues	Atoms					AltConf
			Total	C	Fe	N	O	
5	B	1	86	68	2	8	8	0

Continued on next page...

PRELIMINARY VALIDATION REPORT

Continued from previous page...

Mol	Chain	Residues	Atoms	AltConf
5	B	1	Total C Fe N O<br>86 68 2 8 8	0
5	E	1	Total C Fe N O<br>86 68 2 8 8	0
5	E	1	Total C Fe N O<br>86 68 2 8 8	0

- Molecule 6 is ANTIMYCIN (three-letter code: AMY) (formula: $C_{27}H_{38}N_2O_9$).

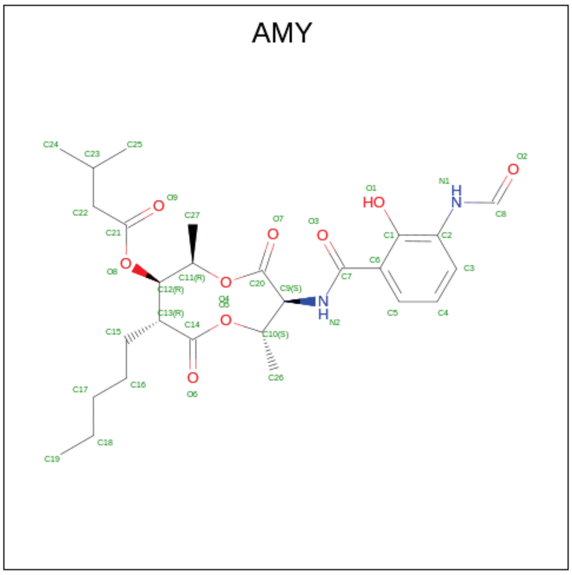

Mol	Chain	Residues	Atoms	AltConf
6	B	1	Total C N O<br>38 27 2 9	0
6	E	1	Total C N O<br>38 27 2 9	0

- Molecule 7 is a ligand with the chemical component id NQA but there is no existing wwPDB Chemical Component Dictionary definition for NQA. Consequently no firm identification of ligand chemistry can be made. Once the structure is annotated then an identification and diagram will be given here.

Mol	Chain	Residues	Atoms	AltConf
7	B	1	Total C O<br>94 90 4	0
7	B	1	Total C O<br>94 90 4	0
7	E	1	Total C O<br>94 90 4	0

Continued on next page...

PRELIMINARY VALIDATION REPORT

Continued from previous page...

Mol	Chain	Residues	Atoms	AltConf
7	E	1	Total C O<br>94 90 4	0

- Molecule 8 is (1R)-2-{[{[(2S)-2,3-DIHYDROXYPROPYL]OXY}(HYDROXY)PHOSPHO RYL]OXY}-1-[(PALMITOYLOXY)METHYL]ETHYL (11E)-OCTADEC-11-ENOATE (three-letter code: PGV) (formula: $C_{40}H_{77}O_{10}P$).

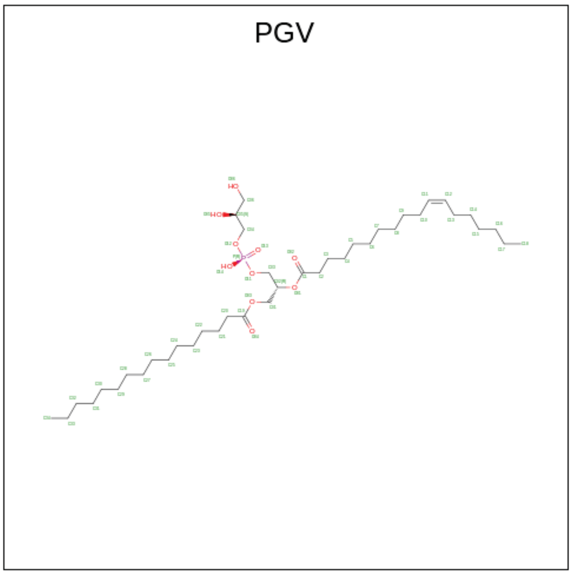

Mol	Chain	Residues	Atoms	AltConf
8	B	1	Total C O P<br>102 80 20 2	0
8	B	1	Total C O P<br>102 80 20 2	0
8	E	1	Total C O P<br>51 40 10 1	0

- Molecule 9 is HEME C (three-letter code: HEC) (formula: $C_{34}H_{34}FeN_4O_4$).

PRELIMINARY VALIDATION REPORT

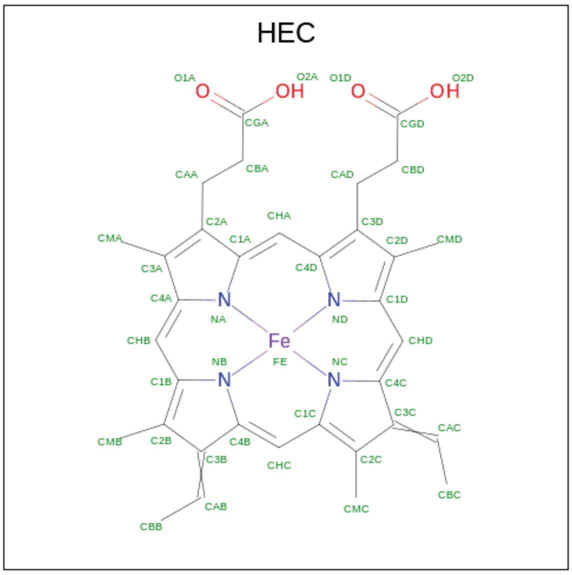

Mol	Chain	Residues	Atoms	AltConf
9	C	1	Total C Fe N O<br>43 34 1 4 4	0
9	F	1	Total C Fe N O<br>43 34 1 4 4	0

PRELIMINARY VALIDATION REPORT

3 Residue-property plots (i)

These plots are drawn for all protein, RNA and DNA chains in the entry. The first graphic for a chain summarises the proportions of the various outlier classes displayed in the second graphic. The second graphic shows the sequence view annotated by issues in geometry. Residues are color-coded according to the number of geometric quality criteria for which they contain at least one outlier: green = 0, yellow = 1, orange = 2 and red = 3 or more. Stretches of 2 or more consecutive residues without any outlier are shown as a green connector. Residues present in the sample, but not in the model, are shown in grey.

- Molecule 1: Rieske-I iron sulfur protein

Chain A:

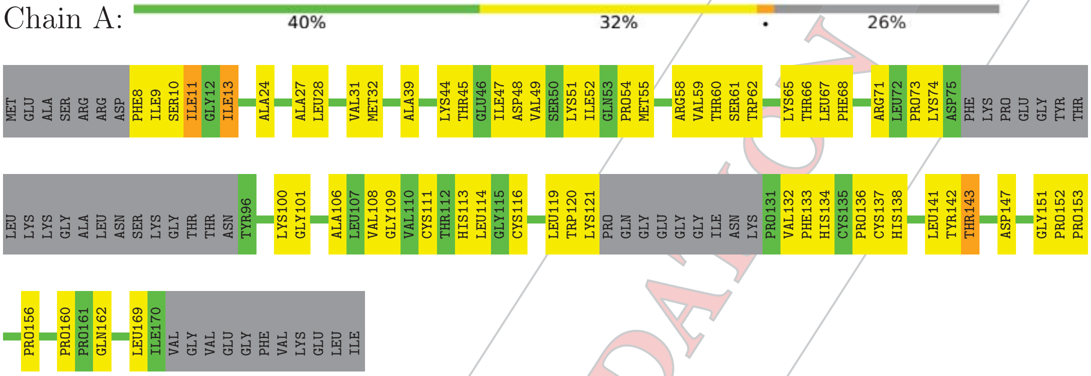

- Molecule 1: Rieske-I iron sulfur protein

Chain D:

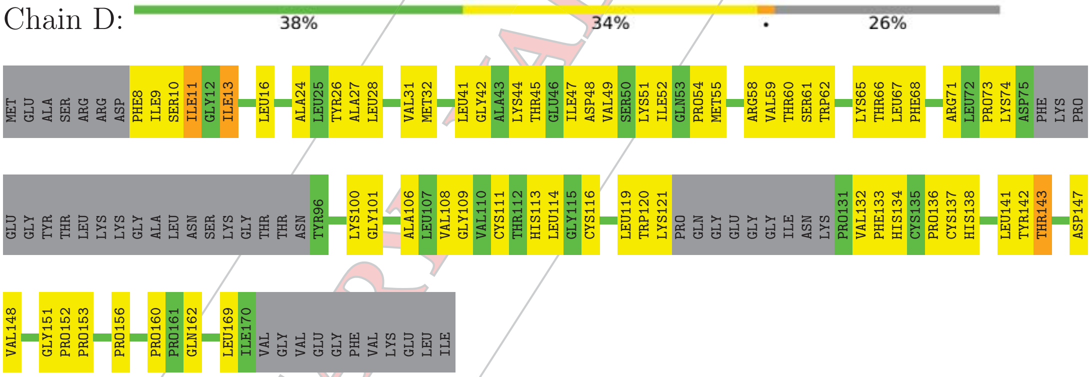

- Molecule 2: cytochrome b subunit

Chain B:

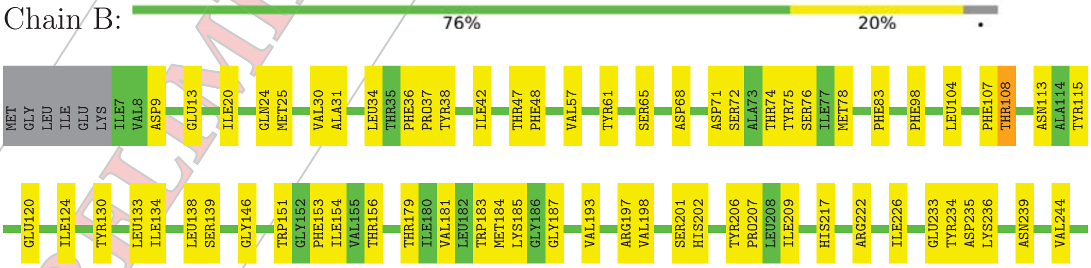

PRELIMINARY VALIDATION REPORT

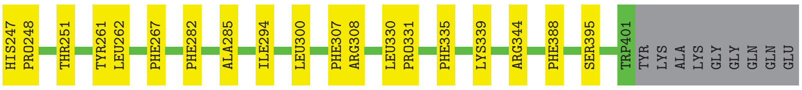

- Molecule 2: cytochrome b subunit

Chain E:

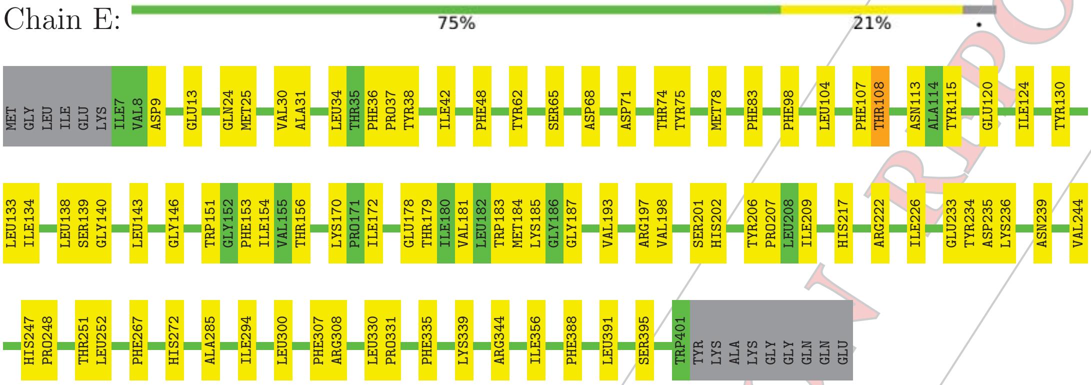

- Molecule 3: Cytochrome c

Chain C:

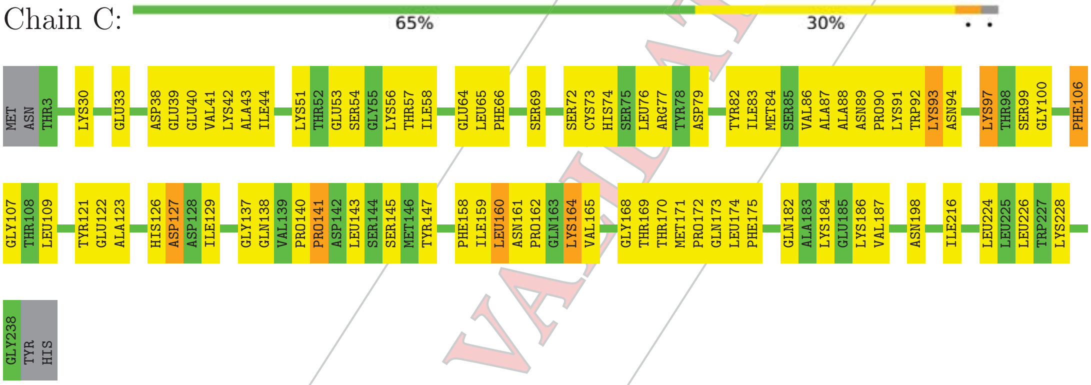

- Molecule 3: Cytochrome c

Chain F:

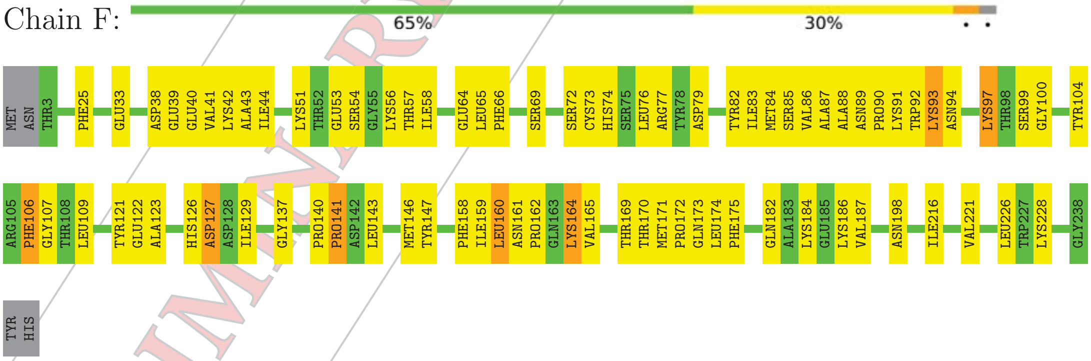

PRELIMINARY VALIDATION REPORT

4 Experimental information ⓘ

Property	Value	Source
Reconstruction method	Not provided	Depositor
Imposed symmetry	POINT, Not provided	Depositor
Number of images used	Not provided	Depositor
Resolution determination method	Not provided	Depositor
CTF correction method	Not provided	Depositor
Microscope	Not provided	Depositor
Voltage (kV)	Not provided	Depositor
Electron dose $(e^{-}/\text{Å}^2)$	Not provided	Depositor
Minimum defocus (nm)	Not provided	Depositor
Maximum defocus (nm)	Not provided	Depositor
Magnification	Not provided	Depositor
Image detector	Not provided	Depositor

PRELIMINARY VALIDATION REPORT

5 Model quality (i)

5.1 Standard geometry (i)

Bond lengths and bond angles in the following residue types are not validated in this section: AMY, PGV, NQA, FES, HEC, HEM

The Z score for a bond length (or angle) is the number of standard deviations the observed value is removed from the expected value. A bond length (or angle) with $|Z| > 5$ is considered an outlier worth inspection. RMSZ is the root-mean-square of all Z scores of the bond lengths (or angles).

Mol	Chain	Bond lengths		Bond angles	
		RMSZ	$\#\|Z\| > 2$	RMSZ	$\#\|Z\| > 2$
1	A	0.34	0/1039	0.52	0/1410
1	D	0.34	0/1039	0.52	0/1410
2	B	0.51	0/3346	0.51	0/4570
2	E	0.51	0/3346	0.51	0/4570
3	C	0.44	0/1974	0.53	0/2674
3	F	0.44	0/1974	0.53	0/2674
All	All	0.46	0/12718	0.52	0/17308

Chiral center outliers are detected by calculating the chiral volume of a chiral center and verifying if the center is modelled as a planar moiety or with the opposite hand.A planarity outlier is detected by checking planarity of atoms in a peptide group, atoms in a mainchain group or atoms of a sidechain that are expected to be planar.

Mol	Chain	#Chirality outliers	#Planarity outliers
1	A	0	1
1	D	0	1
2	B	0	1
2	E	0	1
All	All	0	4

There are no bond length outliers.

There are no bond angle outliers.

There are no chirality outliers.

All (4) planarity outliers are listed below:

Mol	Chain	Res	Type	Group
1	A	151	GLY	Peptide
2	B	108	THR	Peptide
1	D	151	GLY	Peptide

Continued on next page...

PRELIMINARY VALIDATION REPORT

Continued from previous page...

Mol	Chain	Res	Type	Group
2	E	108	THR	Peptide

5.2 Too-close contacts (i)

In the following table, the Non-H and H(model) columns list the number of non-hydrogen atoms and hydrogen atoms in the chain respectively. The H(added) column lists the number of hydrogen atoms added and optimized by MolProbity. The Clashes column lists the number of clashes within the asymmetric unit, whereas Symm-Clashes lists symmetry related clashes.

Mol	Chain	Non-H	H(model)	H(added)	Clashes	Symm-Clashes
1	A	1012	0	1057	44	0
1	D	1012	0	1057	51	0
2	B	3226	0	3280	67	0
2	E	3226	0	3280	73	0
3	C	1917	0	1929	70	0
3	F	1917	0	1929	72	0
4	A	4	0	0	1	0
4	D	4	0	0	0	0
5	B	86	0	60	8	0
5	E	86	0	60	8	0
6	B	38	0	37	25	0
6	E	38	0	37	20	0
7	B	94	0	0	6	0
7	E	94	0	0	11	0
8	B	102	0	152	6	0
8	E	51	0	76	4	0
9	C	43	0	29	6	0
9	F	43	0	29	8	0
All	All	12993	0	13012	395	0

The all-atom clashscore is defined as the number of clashes found per 1000 atoms (including hydrogen atoms). The all-atom clashscore for this structure is 15.

All (395) close contacts within the same asymmetric unit are listed below, sorted by their clash magnitude.

Atom-1	Atom-2	Interatomic distance (Å)	Clash overlap (Å)
3:C:161:ASN:ND2	3:C:164:LYS:HB2	1.43	1.31
2:E:42:ILE:HD11	6:E:503:AMY:O1	1.27	1.30
2:B:42:ILE:HD11	6:B:503:AMY:O1	1.23	1.27
3:F:161:ASN:ND2	3:F:164:LYS:HB2	1.54	1.22

Continued on next page...

PRELIMINARY VALIDATION REPORT

Continued from previous page...

Atom-1	Atom-2	Interatomic distance (Å)	Clash overlap (Å)
6:B:503:AMY:H162	6:B:503:AMY:H253	1.28	1.09
6:E:503:AMY:H13	6:E:503:AMY:H192	1.09	1.08
6:B:503:AMY:H13	6:B:503:AMY:H192	1.10	1.05
3:F:161:ASN:ND2	3:F:164:LYS:HD3	1.72	1.05
3:C:161:ASN:ND2	3:C:164:LYS:HD3	1.73	1.03
2:E:222:ARG:HH12	6:E:503:AMY:H193	1.23	1.01
3:F:161:ASN:HD21	3:F:164:LYS:HD3	1.26	0.96
6:B:503:AMY:H152	7:B:504:NQA:C46	1.96	0.95
6:E:503:AMY:H192	6:E:503:AMY:C13	1.95	0.94
6:E:503:AMY:C19	6:E:503:AMY:H13	1.97	0.94
3:C:161:ASN:HD21	3:C:164:LYS:HD3	1.31	0.93
6:B:503:AMY:H13	6:B:503:AMY:C19	2.01	0.91
6:B:503:AMY:C25	6:B:503:AMY:H162	2.01	0.90
5:E:501:HEM:HBB2	5:E:501:HEM:HHC	1.55	0.87
2:B:42:ILE:CD1	6:B:503:AMY:O1	2.18	0.87
5:B:501:HEM:HHC	5:B:501:HEM:HBB2	1.55	0.87
3:C:161:ASN:ND2	3:C:164:LYS:CB	2.34	0.86
6:B:503:AMY:C13	6:B:503:AMY:H192	2.02	0.86
3:F:159:ILE:HD13	9:F:501:HEC:HMB2	1.54	0.86
5:E:501:HEM:HHD	5:E:501:HEM:HBC2	1.60	0.83
3:C:161:ASN:HD21	3:C:164:LYS:HB2	1.44	0.83
5:B:501:HEM:HBC2	5:B:501:HEM:HHD	1.60	0.82
3:C:159:ILE:HD13	9:C:501:HEC:HMB2	1.64	0.80
3:F:159:ILE:CD1	9:F:501:HEC:HMB2	2.12	0.80
2:E:42:ILE:CD1	6:E:503:AMY:O1	2.21	0.80
2:B:37:PRO:HG3	8:B:506:PGV:H012	1.66	0.78
2:B:20:ILE:HD13	8:B:507:PGV:H222	1.67	0.77
2:E:37:PRO:HG3	8:E:506:PGV:H012	1.66	0.76
2:B:113:ASN:ND2	2:B:339:LYS:O	2.16	0.76
1:A:24:ALA:HB1	7:B:505:NQA:C30	2.15	0.75
2:E:34:LEU:CD1	6:E:503:AMY:H3	2.17	0.75
3:F:161:ASN:ND2	3:F:164:LYS:CB	2.44	0.74
1:A:111:CYS:HG	1:A:142:TYR:HH	1.27	0.74
1:A:134:HIS:HB3	1:A:141:LEU:HD23	1.70	0.74
2:E:113:ASN:ND2	2:E:339:LYS:O	2.16	0.74
3:C:159:ILE:CD1	9:C:501:HEC:HMB2	2.17	0.73
2:E:34:LEU:HD13	6:E:503:AMY:H3	1.70	0.73
1:D:111:CYS:HG	1:D:142:TYR:HH	1.32	0.73
1:D:134:HIS:HB3	1:D:141:LEU:HD23	1.70	0.73
1:A:147:ASP:HA	1:A:156:PRO:HB3	1.72	0.72
2:B:34:LEU:HD13	6:B:503:AMY:H3	1.72	0.72

Continued on next page...

PRELIMINARY VALIDATION REPORT

Continued from previous page...

Atom-1	Atom-2	Interatomic distance (Å)	Clash overlap (Å)
6:B:503:AMY:C21	6:B:503:AMY:H273	2.19	0.71
2:B:34:LEU:CD1	6:B:503:AMY:H3	2.20	0.71
2:B:222:ARG:HH12	6:B:503:AMY:H193	1.55	0.71
1:D:147:ASP:HA	1:D:156:PRO:HB3	1.72	0.71
3:C:169:THR:HG22	3:C:171:MET:H	1.56	0.71
3:F:169:THR:HG22	3:F:171:MET:H	1.56	0.71
3:F:161:ASN:HD21	3:F:164:LYS:HB2	1.52	0.69
2:E:222:ARG:NH1	6:E:503:AMY:H193	2.04	0.68
1:D:28:LEU:HD11	7:E:505:NQA:C29	2.24	0.68
3:C:73:CYS:HB3	3:C:140:PRO:HG2	1.77	0.67
3:F:73:CYS:HB3	3:F:140:PRO:HG2	1.77	0.66
6:B:503:AMY:O8	6:B:503:AMY:H253	1.96	0.66
2:E:24:GLN:OE1	2:E:222:ARG:NH2	2.31	0.64
3:C:160:LEU:HB3	3:C:187:VAL:HG21	1.79	0.64
1:D:8:PHE:HE2	1:D:10:SER:HG	1.46	0.64
1:A:100:LYS:HG3	1:A:101:GLY:H	1.64	0.63
3:C:41:VAL:HG23	3:C:42:LYS:H	1.63	0.63
3:F:41:VAL:HG23	3:F:42:LYS:H	1.63	0.63
2:B:24:GLN:OE1	2:B:222:ARG:NH2	2.31	0.63
1:D:100:LYS:HG3	1:D:101:GLY:H	1.64	0.63
2:E:30:VAL:CG2	6:E:503:AMY:H4	2.28	0.63
2:E:248:PRO:HA	2:E:251:THR:HG22	1.81	0.62
3:F:160:LEU:HB3	3:F:187:VAL:HG21	1.80	0.62
3:F:38:ASP:OD2	3:F:184:LYS:NZ	2.33	0.62
3:F:44:ILE:HG23	3:F:58:ILE:HD13	1.81	0.62
2:B:248:PRO:HA	2:B:251:THR:HG22	1.82	0.61
2:B:30:VAL:CG2	6:B:503:AMY:H4	2.30	0.61
3:C:44:ILE:HG23	3:C:58:ILE:HD13	1.81	0.61
3:C:65:LEU:HD11	3:C:186:LYS:HD2	1.83	0.61
6:E:503:AMY:H261	6:E:503:AMY:O3	2.00	0.61
2:E:226:ILE:HD13	6:E:503:AMY:C4	2.30	0.61
2:B:226:ILE:HD13	6:B:503:AMY:C4	2.31	0.61
3:C:38:ASP:OD2	3:C:184:LYS:NZ	2.33	0.61
1:A:8:PHE:HE2	1:A:10:SER:HG	1.49	0.61
3:C:161:ASN:HD22	3:C:164:LYS:HB2	1.60	0.61
3:C:147:TYR:HB3	3:C:198:ASN:HD22	1.66	0.60
6:E:503:AMY:C21	6:E:503:AMY:H273	2.31	0.60
3:F:56:LYS:NZ	3:F:64:GLU:OE1	2.34	0.60
1:A:113:HIS:HB3	1:A:153:PRO:HA	1.83	0.60
1:D:111:CYS:HB3	1:D:116:CYS:H	1.67	0.60
6:B:503:AMY:H261	6:B:503:AMY:O3	2.02	0.59

Continued on next page...

PRELIMINARY VALIDATION REPORT

Continued from previous page...

Atom-1	Atom-2	Interatomic distance (Å)	Clash overlap (Å)
3:C:56:LYS:NZ	3:C:64:GLU:OE1	2.34	0.59
1:D:113:HIS:HB3	1:D:153:PRO:HA	1.83	0.59
2:E:307:PHE:O	2:E:308:ARG:HG3	2.02	0.59
3:F:147:TYR:HB3	3:F:198:ASN:HD22	1.66	0.59
3:F:65:LEU:HD11	3:F:186:LYS:HD2	1.83	0.59
6:E:503:AMY:O9	6:E:503:AMY:H273	2.03	0.59
3:F:161:ASN:ND2	3:F:164:LYS:CD	2.59	0.59
1:A:111:CYS:HB3	1:A:116:CYS:H	1.67	0.59
9:C:501:HEC:HBA2	9:C:501:HEC:HHA	1.84	0.58
2:E:247:HIS:CD2	2:E:251:THR:HG21	2.38	0.58
3:C:161:ASN:ND2	3:C:164:LYS:CD	2.58	0.58
2:B:247:HIS:CD2	2:B:251:THR:HG21	2.38	0.58
2:E:226:ILE:HD12	5:E:502:HEM:HBA2	1.85	0.58
9:F:501:HEC:HBA2	9:F:501:HEC:HHA	1.84	0.58
2:B:226:ILE:HD12	5:B:502:HEM:HBA2	1.85	0.58
2:B:307:PHE:O	2:B:308:ARG:HG3	2.02	0.57
3:C:123:ALA:HA	3:C:127:ASP:HB2	1.86	0.57
3:F:123:ALA:HA	3:F:127:ASP:HB2	1.86	0.57
3:C:138:GLN:HB2	1:D:137:CYS:SG	2.44	0.56
3:C:172:PRO:HD3	9:C:501:HEC:HBC2	1.87	0.56
5:E:502:HEM:HMC2	5:E:502:HEM:HBC2	1.87	0.56
2:B:151:TRP:HB3	2:B:294:ILE:HG12	1.88	0.56
1:A:60:THR:HG22	1:A:61:SER:H	1.71	0.56
3:F:172:PRO:HD3	9:F:501:HEC:HBC2	1.87	0.56
2:E:151:TRP:HB3	2:E:294:ILE:HG12	1.88	0.55
3:F:161:ASN:HD21	3:F:164:LYS:CD	2.11	0.55
3:C:137:GLY:HA3	1:D:136:PRO:HG2	1.88	0.55
6:E:503:AMY:H162	6:E:503:AMY:O8	2.07	0.55
5:B:502:HEM:HBA1	5:B:502:HEM:HHA	1.88	0.55
5:B:502:HEM:HBC2	5:B:502:HEM:HMC2	1.87	0.55
1:D:60:THR:HG22	1:D:61:SER:H	1.71	0.55
1:D:106:ALA:O	1:D:162:GLN:NE2	2.40	0.54
5:E:502:HEM:HBA1	5:E:502:HEM:HHA	1.88	0.54
1:D:27:ALA:HB2	7:E:505:NQA:C46	2.37	0.54
2:E:65:SER:HB2	2:E:68:ASP:HB2	1.89	0.54
7:E:504:NQA:C46	7:E:504:NQA:C22	2.86	0.54
1:A:65:LYS:HB3	1:A:66:THR:HG22	1.89	0.54
3:C:99:SER:N	3:C:100:GLY:HA3	2.22	0.54
2:E:9:ASP:O	2:E:13:GLU:HG3	2.08	0.54
1:A:49:VAL:HG21	1:A:169:LEU:HD23	1.90	0.54
1:A:39:ALA:HB1	2:E:187:GLY:HA3	1.90	0.54

Continued on next page...

PRELIMINARY VALIDATION REPORT

Continued from previous page...

Atom-1	Atom-2	Interatomic distance (Å)	Clash overlap (Å)
3:C:161:ASN:HD21	3:C:164:LYS:CD	2.12	0.54
2:B:197:ARG:NH1	1:D:32:MET:O	2.41	0.54
2:B:9:ASP:O	2:B:13:GLU:HG3	2.08	0.54
3:F:91:LYS:HA	3:F:92:TRP:C	2.27	0.54
2:B:65:SER:HB2	2:B:68:ASP:HB2	1.89	0.53
3:F:99:SER:N	3:F:100:GLY:HA3	2.22	0.53
1:A:106:ALA:O	1:A:162:GLN:NE2	2.41	0.53
2:E:30:VAL:HG21	6:E:503:AMY:C4	2.37	0.53
3:C:74:HIS:HE1	3:C:141:PRO:HD2	1.74	0.53
3:C:91:LYS:HA	3:C:92:TRP:C	2.27	0.53
1:D:49:VAL:HG21	1:D:169:LEU:HD23	1.90	0.53
1:A:62:TRP:HB2	1:A:67:LEU:HD22	1.91	0.53
3:C:39:GLU:HA	3:C:42:LYS:HB2	1.91	0.53
1:D:65:LYS:HB3	1:D:66:THR:HG22	1.89	0.53
1:A:31:VAL:HG11	2:B:57:VAL:HG13	1.91	0.52
3:F:74:HIS:HE1	3:F:141:PRO:HD2	1.74	0.52
1:D:31:VAL:HG22	2:E:83:PHE:HB2	1.91	0.52
2:B:217:HIS:NE2	5:B:502:HEM:ND	2.57	0.52
2:B:235:ASP:OD2	2:B:239:ASN:ND2	2.43	0.52
7:B:504:NQA:C36	2:E:209:ILE:HG22	2.40	0.52
3:C:86:VAL:HG11	3:C:92:TRP:CD1	2.45	0.52
2:B:30:VAL:HG21	6:B:503:AMY:C4	2.39	0.52
2:E:335:PHE:O	2:E:395:SER:HB3	2.09	0.52
5:B:502:HEM:HBB2	5:B:502:HEM:HMB1	1.91	0.52
2:B:335:PHE:O	2:B:395:SER:HB3	2.09	0.52
2:E:217:HIS:NE2	5:E:502:HEM:ND	2.58	0.51
6:B:503:AMY:H162	6:B:503:AMY:O8	2.10	0.51
1:D:62:TRP:HB2	1:D:67:LEU:HD22	1.91	0.51
2:E:30:VAL:HG21	6:E:503:AMY:H4	1.91	0.51
2:B:30:VAL:HG21	6:B:503:AMY:H4	1.92	0.51
3:C:127:ASP:OD1	3:F:97:LYS:NZ	2.43	0.51
3:F:39:GLU:HA	3:F:42:LYS:HB2	1.91	0.51
3:F:86:VAL:HG11	3:F:92:TRP:CD1	2.45	0.51
5:E:502:HEM:HMB1	5:E:502:HEM:HBB2	1.91	0.51
1:D:111:CYS:SG	1:D:142:TYR:OH	2.48	0.51
1:D:132:VAL:HG12	1:D:143:THR:HA	1.93	0.51
3:F:72:SER:HA	3:F:84:MET:HG2	1.93	0.51
3:C:72:SER:HA	3:C:84:MET:HG2	1.93	0.50
2:E:235:ASP:OD2	2:E:239:ASN:ND2	2.43	0.50
1:A:27:ALA:O	1:A:31:VAL:HG23	2.12	0.50
1:D:27:ALA:O	1:D:31:VAL:HG23	2.12	0.50

Continued on next page...

PRELIMINARY VALIDATION REPORT

Continued from previous page...

Atom-1	Atom-2	Interatomic distance (Å)	Clash overlap (Å)
3:F:160:LEU:HD12	3:F:160:LEU:C	2.31	0.50
1:A:132:VAL:HG12	1:A:143:THR:HA	1.93	0.50
3:F:171:MET:HG3	9:F:501:HEC:NC	2.27	0.50
3:F:93:LYS:HD3	3:F:93:LYS:N	2.27	0.50
3:C:97:LYS:NZ	3:F:127:ASP:OD1	2.44	0.49
3:C:93:LYS:HD3	3:C:93:LYS:N	2.27	0.49
1:D:28:LEU:CD1	7:E:505:NQA:C29	2.90	0.49
2:B:209:ILE:HG22	7:E:504:NQA:C36	2.42	0.49
3:F:91:LYS:HB3	3:F:94:ASN:N	2.27	0.49
3:C:91:LYS:HB3	3:C:94:ASN:N	2.28	0.49
3:C:69:SER:OG	3:C:69:SER:O	2.30	0.49
3:C:171:MET:HG3	9:C:501:HEC:NC	2.27	0.49
2:B:267:PHE:HA	3:C:216:ILE:HD11	1.95	0.49
3:C:160:LEU:C	3:C:160:LEU:HD12	2.32	0.48
3:C:53:GLU:O	3:C:54:SER:OG	2.30	0.48
1:D:143:THR:HG23	1:D:147:ASP:O	2.13	0.48
1:A:143:THR:HG23	1:A:147:ASP:O	2.13	0.48
3:F:161:ASN:N	3:F:162:PRO:HD3	2.29	0.48
1:D:55:MET:O	1:D:120:TRP:NE1	2.45	0.48
2:E:222:ARG:HH12	6:E:503:AMY:C19	2.09	0.48
3:F:109:LEU:HD23	3:F:109:LEU:H	1.79	0.48
3:C:182:GLN:O	3:C:186:LYS:HG2	2.14	0.48
1:D:24:ALA:HB1	7:E:505:NQA:C30	2.44	0.48
2:E:179:THR:HG23	2:E:183:TRP:HB2	1.95	0.47
1:A:136:PRO:HG2	3:F:137:GLY:HA3	1.96	0.47
2:B:179:THR:HG23	2:B:183:TRP:HB2	1.95	0.47
1:A:31:VAL:HG11	2:B:57:VAL:CG1	2.45	0.47
2:B:113:ASN:ND2	2:B:344:ARG:HH11	2.12	0.47
2:B:120:GLU:O	2:B:124:ILE:HG12	2.15	0.47
2:B:181:VAL:HA	2:B:187:GLY:HA2	1.96	0.47
3:C:86:VAL:O	3:C:89:ASN:N	2.48	0.47
3:F:87:ALA:HA	3:F:88:ALA:HA	1.65	0.47
2:E:138:LEU:HD11	2:E:156:THR:HG21	1.97	0.47
3:C:77:ARG:C	3:C:79:ASP:H	2.17	0.47
2:E:113:ASN:ND2	2:E:344:ARG:HH11	2.12	0.47
2:E:181:VAL:HA	2:E:187:GLY:HA2	1.97	0.47
1:D:49:VAL:O	1:D:71:ARG:NH1	2.45	0.47
3:C:109:LEU:H	3:C:109:LEU:HD23	1.79	0.47
2:E:139:SER:OG	2:E:202:HIS:HB2	2.15	0.47
3:F:182:GLN:O	3:F:186:LYS:HG2	2.14	0.47
2:B:138:LEU:HD11	2:B:156:THR:HG21	1.97	0.46

Continued on next page...

PRELIMINARY VALIDATION REPORT

Continued from previous page...

Atom-1	Atom-2	Interatomic distance (Å)	Clash overlap (Å)
3:F:86:VAL:O	3:F:89:ASN:N	2.48	0.46
2:B:236:LYS:HD2	2:B:236:LYS:HA	1.67	0.46
1:A:45:THR:O	1:A:47:ILE:HG13	2.16	0.46
2:B:139:SER:OG	2:B:202:HIS:HB2	2.15	0.46
2:E:120:GLU:O	2:E:124:ILE:HG12	2.15	0.46
3:F:69:SER:OG	3:F:69:SER:O	2.30	0.46
3:F:77:ARG:C	3:F:79:ASP:H	2.17	0.46
2:E:267:PHE:HA	3:F:216:ILE:HD11	1.97	0.46
3:F:106:PHE:HA	3:F:107:GLY:HA2	1.71	0.46
3:F:121:TYR:O	3:F:122:GLU:HG2	2.15	0.46
3:C:170:THR:HG21	1:D:136:PRO:O	2.16	0.46
3:C:121:TYR:O	3:C:122:GLU:HG2	2.15	0.46
1:A:73:PRO:C	1:A:74:LYS:HD3	2.37	0.45
3:F:161:ASN:HD22	3:F:164:LYS:HD3	1.74	0.45
3:C:161:ASN:N	3:C:162:PRO:HD3	2.32	0.45
1:A:31:VAL:O	2:B:61:TYR:OH	2.34	0.45
3:C:170:THR:HG23	1:D:137:CYS:O	2.16	0.45
1:D:16:LEU:HD21	3:F:221:VAL:HG13	1.98	0.45
3:C:69:SER:OG	3:C:174:LEU:HD21	2.17	0.45
3:F:69:SER:OG	3:F:174:LEU:HD21	2.17	0.45
2:B:47:THR:OG1	2:B:261:TYR:OH	2.16	0.45
1:D:73:PRO:C	1:D:74:LYS:HD3	2.37	0.45
3:F:160:LEU:C	3:F:160:LEU:CD1	2.85	0.45
2:B:83:PHE:HZ	7:B:505:NQA:C16	2.30	0.45
3:F:53:GLU:O	3:F:54:SER:OG	2.30	0.45
1:D:45:THR:O	1:D:47:ILE:HG13	2.15	0.45
2:B:71:ASP:O	2:B:75:TYR:HB3	2.17	0.45
2:E:83:PHE:HZ	7:E:505:NQA:C16	2.30	0.45
1:A:49:VAL:O	1:A:71:ARG:NH1	2.45	0.45
1:D:13:ILE:H	1:D:13:ILE:HG12	1.49	0.45
1:D:152:PRO:N	1:D:153:PRO:HD2	2.32	0.45
1:A:32:MET:O	2:E:197:ARG:HD3	2.16	0.44
2:E:71:ASP:O	2:E:75:TYR:HB3	2.17	0.44
2:B:244:VAL:HG13	2:B:244:VAL:O	2.17	0.44
2:B:48:PHE:CE1	5:B:501:HEM:HBB1	2.52	0.44
1:A:28:LEU:HD11	7:B:505:NQA:C43	2.47	0.44
2:E:193:VAL:CG1	2:E:197:ARG:HH21	2.30	0.44
2:E:48:PHE:CE1	5:E:501:HEM:HBB1	2.52	0.44
3:F:228:LYS:HA	3:F:228:LYS:HD3	1.68	0.44
2:B:193:VAL:CG1	2:B:197:ARG:HH21	2.30	0.44
2:B:104:LEU:HD23	8:B:506:PGV:H221	1.99	0.44

Continued on next page...

PRELIMINARY VALIDATION REPORT

Continued from previous page...

Atom-1	Atom-2	Interatomic distance (Å)	Clash overlap (Å)
3:C:160:LEU:C	3:C:160:LEU:CD1	2.85	0.44
3:C:126:HIS:CE1	3:C:129:ILE:HG23	2.52	0.44
2:E:130:TYR:CZ	2:E:134:ILE:HD11	2.53	0.44
1:D:26:TYR:HD2	7:E:505:NQA:C46	2.30	0.44
3:F:126:HIS:CE1	3:F:129:ILE:HG23	2.52	0.44
2:B:113:ASN:HB2	2:B:339:LYS:O	2.18	0.44
2:B:146:GLY:O	2:B:285:ALA:HB2	2.18	0.44
2:E:154:ILE:HD12	2:E:185:LYS:HE3	2.00	0.44
1:A:65:LYS:HA	1:A:66:THR:HA	1.59	0.44
1:D:41:LEU:HA	1:D:42:GLY:HA2	1.65	0.44
1:A:137:CYS:O	3:F:170:THR:HG23	2.18	0.44
1:A:152:PRO:N	1:A:153:PRO:HD2	2.32	0.44
1:A:138:HIS:ND1	4:A:501:FES:S2	2.84	0.44
3:F:171:MET:HG3	9:F:501:HEC:C4C	2.48	0.44
3:C:171:MET:HG3	9:C:501:HEC:C4C	2.48	0.44
3:F:51:LYS:HD2	3:F:57:THR:HG22	1.99	0.44
2:B:115:TYR:HB2	2:B:331:PRO:HB3	2.00	0.43
2:E:113:ASN:HB2	2:E:339:LYS:O	2.18	0.43
3:F:91:LYS:HB2	3:F:91:LYS:HE2	1.82	0.43
2:B:130:TYR:CZ	2:B:134:ILE:HD11	2.53	0.43
2:E:252:LEU:HD22	3:F:226:LEU:HD22	2.00	0.43
2:E:146:GLY:O	2:E:285:ALA:HB2	2.18	0.43
2:E:138:LEU:HD12	2:E:300:LEU:HD11	2.00	0.43
1:D:26:TYR:HE2	7:E:505:NQA:C16	2.32	0.43
2:B:138:LEU:HD12	2:B:300:LEU:HD11	2.01	0.43
2:E:115:TYR:HB2	2:E:331:PRO:HB3	2.00	0.43
3:F:158:PHE:HA	3:F:165:VAL:HG21	2.00	0.43
2:B:98:PHE:CE2	2:B:133:LEU:HD11	2.54	0.43
3:C:168:GLY:O	1:D:138:HIS:HB3	2.17	0.43
2:E:104:LEU:HD23	8:E:506:PGV:H221	1.99	0.43
2:E:98:PHE:CE2	2:E:133:LEU:HD11	2.54	0.43
3:F:161:ASN:CG	3:F:164:LYS:HB2	2.31	0.43
1:A:54:PRO:HB3	1:A:73:PRO:HD3	2.01	0.43
2:E:153:PHE:HZ	2:E:184:MET:HB2	1.84	0.43
1:A:9:ILE:O	1:A:13:ILE:HG12	2.18	0.43
2:B:153:PHE:HZ	2:B:184:MET:HB2	1.84	0.43
2:B:154:ILE:HD12	2:B:185:LYS:HE3	2.00	0.43
2:B:30:VAL:HG13	2:B:31:ALA:O	2.19	0.43
2:E:244:VAL:HG13	2:E:244:VAL:O	2.17	0.43
1:D:24:ALA:HB1	7:E:505:NQA:C28	2.48	0.43
2:B:107:PHE:HE2	8:B:506:PGV:H311	1.84	0.43

Continued on next page...

PRELIMINARY VALIDATION REPORT

Continued from previous page...

Atom-1	Atom-2	Interatomic distance (Å)	Clash overlap (Å)
1:D:68:PHE:CE2	1:D:109:GLY:HA3	2.53	0.43
1:D:9:ILE:O	1:D:13:ILE:HG12	2.18	0.43
3:F:92:TRP:HA	3:F:93:LYS:HA	1.84	0.43
3:C:158:PHE:HA	3:C:165:VAL:HG21	2.00	0.43
3:C:228:LYS:HA	3:C:228:LYS:HD3	1.67	0.43
3:C:83:ILE:O	3:C:89:ASN:HB2	2.19	0.43
7:E:505:NQA:C47	7:E:505:NQA:C18	2.96	0.43
3:F:41:VAL:C	3:F:43:ALA:H	2.22	0.43
1:A:13:ILE:H	1:A:13:ILE:HG12	1.49	0.42
7:B:505:NQA:C47	7:B:505:NQA:C18	2.96	0.42
3:C:51:LYS:HD2	3:C:57:THR:HG22	1.99	0.42
1:D:51:LYS:HZ1	1:D:58:ARG:HH11	1.66	0.42
2:E:30:VAL:HG13	2:E:31:ALA:O	2.19	0.42
1:A:68:PHE:CE2	1:A:109:GLY:HA3	2.53	0.42
1:A:114:LEU:HD23	1:A:114:LEU:HA	1.89	0.42
3:C:87:ALA:HA	3:C:88:ALA:HA	1.65	0.42
2:E:356:ILE:HA	2:E:356:ILE:HD13	1.92	0.42
6:E:503:AMY:N2	6:E:503:AMY:O1	2.51	0.42
3:F:66:PHE:CE1	3:F:143:LEU:HD12	2.54	0.42
3:F:85:SER:HA	3:F:86:VAL:HA	1.90	0.42
1:A:121:LYS:H	1:A:133:PHE:HB3	1.84	0.42
3:C:41:VAL:C	3:C:43:ALA:H	2.22	0.42
1:D:121:LYS:H	1:D:133:PHE:HB3	1.84	0.42
6:B:503:AMY:O1	6:B:503:AMY:N2	2.52	0.42
2:B:74:THR:O	2:B:78:MET:HB2	2.20	0.42
2:B:201:SER:OG	2:B:202:HIS:N	2.52	0.42
2:B:206:TYR:HB2	2:B:207:PRO:HD3	2.02	0.42
6:B:503:AMY:C25	6:B:503:AMY:H273	2.49	0.42
2:E:201:SER:OG	2:E:202:HIS:N	2.52	0.42
2:E:36:PHE:N	2:E:37:PRO:HD2	2.35	0.42
2:B:282:PHE:O	3:C:145:SER:OG	2.36	0.42
3:C:226:LEU:HD23	3:C:226:LEU:HA	1.86	0.42
3:C:66:PHE:CE1	3:C:143:LEU:HD12	2.54	0.42
2:E:62:TYR:OH	2:E:143:LEU:O	2.31	0.42
2:E:206:TYR:HB2	2:E:207:PRO:HD3	2.02	0.42
3:F:82:TYR:HB2	3:F:90:PRO:HD3	2.02	0.42
2:B:36:PHE:N	2:B:37:PRO:HD2	2.35	0.42
3:C:30:LYS:HE3	3:C:30:LYS:HB3	1.89	0.42
2:E:107:PHE:HE2	8:E:506:PGV:H311	1.84	0.42
1:A:51:LYS:HZ1	1:A:58:ARG:HH11	1.67	0.42
3:C:82:TYR:HB2	3:C:90:PRO:HD3	2.02	0.42

Continued on next page...

PRELIMINARY VALIDATION REPORT

Continued from previous page...

Atom-1	Atom-2	Interatomic distance (Å)	Clash overlap (Å)
2:E:193:VAL:HG12	2:E:197:ARG:HH21	1.85	0.42
1:A:8:PHE:HB3	1:A:11:ILE:HG22	2.01	0.42
1:D:108:VAL:H	1:D:160:PRO:HD3	1.85	0.42
6:B:503:AMY:C7	6:B:503:AMY:H261	2.49	0.41
1:D:54:PRO:HB3	1:D:73:PRO:HD3	2.01	0.41
2:E:236:LYS:HD2	2:E:236:LYS:HA	1.67	0.41
2:E:74:THR:O	2:E:78:MET:HB2	2.20	0.41
3:F:159:ILE:HD11	9:F:501:HEC:HMB2	1.99	0.41
2:B:193:VAL:HG12	2:B:197:ARG:HH21	1.85	0.41
3:C:169:THR:HA	1:D:138:HIS:HD2	1.85	0.41
3:C:33:GLU:OE1	3:C:33:GLU:HA	2.21	0.41
1:A:113:HIS:CD2	1:A:114:LEU:HG	2.55	0.41
2:B:262:LEU:HA	2:B:262:LEU:HD23	1.89	0.41
6:B:503:AMY:C27	6:B:503:AMY:C21	2.86	0.41
3:C:224:LEU:HA	3:C:224:LEU:HD12	1.85	0.41
3:C:91:LYS:HB2	3:C:91:LYS:HE2	1.82	0.41
2:B:38:TYR:CD1	2:B:226:ILE:HD11	2.56	0.41
2:B:107:PHE:CE2	8:B:506:PGV:H311	2.56	0.41
3:C:76:LEU:HD12	3:C:143:LEU:HB2	2.02	0.41
1:D:113:HIS:CD2	1:D:114:LEU:HG	2.55	0.41
3:F:33:GLU:OE1	3:F:33:GLU:HA	2.20	0.41
3:F:83:ILE:O	3:F:89:ASN:HB2	2.19	0.41
1:A:108:VAL:H	1:A:160:PRO:HD3	1.85	0.41
6:B:503:AMY:H182	6:B:503:AMY:H272	2.02	0.41
1:D:49:VAL:O	1:D:49:VAL:HG12	2.21	0.41
2:B:233:GLU:HG3	2:B:234:TYR:HD1	1.85	0.41
3:C:38:ASP:C	3:C:40:GLU:H	2.24	0.41
2:E:139:SER:HB2	2:E:198:VAL:HG13	2.03	0.41
3:F:146:MET:HG2	9:F:501:HEC:HMA3	2.03	0.41
3:F:99:SER:O	3:F:99:SER:OG	2.34	0.41
1:A:48:ASP:O	1:A:51:LYS:HG2	2.20	0.41
1:D:8:PHE:HB3	1:D:11:ILE:HG22	2.01	0.41
2:E:272:HIS:CE1	3:F:25:PHE:HB3	2.56	0.41
1:A:55:MET:O	1:A:120:TRP:NE1	2.45	0.41
6:B:503:AMY:H11	6:B:503:AMY:O9	2.21	0.41
1:D:48:ASP:O	1:D:51:LYS:HG2	2.20	0.41
6:E:503:AMY:C7	6:E:503:AMY:H261	2.50	0.41
3:F:76:LEU:HD12	3:F:143:LEU:HB2	2.02	0.41
3:F:173:GLN:C	3:F:175:PHE:H	2.24	0.41
3:C:106:PHE:HA	3:C:107:GLY:HA2	1.71	0.41
3:C:173:GLN:C	3:C:175:PHE:H	2.24	0.41

Continued on next page...

PRELIMINARY VALIDATION REPORT

Continued from previous page...

Atom-1	Atom-2	Interatomic distance (Å)	Clash overlap (Å)
3:F:38:ASP:C	3:F:40:GLU:H	2.24	0.41
2:E:38:TYR:CD1	2:E:226:ILE:HD11	2.56	0.40
2:E:247:HIS:HA	2:E:248:PRO:HA	1.87	0.40
3:F:104:TYR:HA	3:F:109:LEU:HA	2.03	0.40
2:B:25:MET:HB3	2:B:247:HIS:HB2	2.03	0.40
1:D:119:LEU:O	1:D:133:PHE:HB2	2.22	0.40
2:E:178:GLU:HG2	2:E:181:VAL:HG12	2.03	0.40
2:E:25:MET:HB3	2:E:247:HIS:HB2	2.03	0.40
2:E:391:LEU:HD23	2:E:391:LEU:HA	1.87	0.40
1:A:119:LEU:O	1:A:133:PHE:HB2	2.22	0.40
1:A:49:VAL:O	1:A:49:VAL:HG12	2.21	0.40
2:E:107:PHE:CE2	8:E:506:PGV:H311	2.56	0.40
2:E:233:GLU:HG3	2:E:234:TYR:HD1	1.85	0.40
2:E:294:ILE:HD12	2:E:294:ILE:HA	1.88	0.40
2:B:139:SER:HB2	2:B:198:VAL:HG13	2.03	0.40
2:B:330:LEU:HD12	2:B:388:PHE:HE1	1.86	0.40
2:B:72:SER:O	2:B:76:SER:HB3	2.22	0.40
1:D:148:VAL:HG11	1:D:153:PRO:HG2	2.04	0.40
2:E:140:GLY:HA3	2:E:202:HIS:CE1	2.57	0.40
8:B:507:PGV:H311	8:B:507:PGV:H282	1.89	0.40
2:E:170:LYS:HE3	2:E:172:ILE:HG21	2.03	0.40
2:E:330:LEU:HD12	2:E:388:PHE:HE1	1.86	0.40

There are no symmetry-related clashes.

5.3 Torsion angles (i)

5.3.1 Protein backbone (i)

In the following table, the Percentiles column shows the percent Ramachandran outliers of the chain as a percentile score with respect to all PDB entries followed by that with respect to all EM entries.

The Analysed column shows the number of residues for which the backbone conformation was analysed, and the total number of residues.

Mol	Chain	Analysed	Favoured	Allowed	Outliers	Percentiles	
1	A	128/181 (71%)	107 (84%)	21 (16%)	0	100	100
1	D	128/181 (71%)	107 (84%)	21 (16%)	0	100	100
2	B	393/410 (96%)	362 (92%)	31 (8%)	0	100	100

Continued on next page...

PRELIMINARY VALIDATION REPORT

Continued from previous page...

Mol	Chain	Analysed	Favoured	Allowed	Outliers	Percentiles	
2	E	393/410 (96%)	362 (92%)	31 (8%)	0	100	100
3	C	234/240 (98%)	205 (88%)	28 (12%)	1 (0%)	36	36
3	F	234/240 (98%)	205 (88%)	28 (12%)	1 (0%)	36	36
All	All	1510/1662 (91%)	1348 (89%)	160 (11%)	2 (0%)	56	53

All (2) Ramachandran outliers are listed below:

Mol	Chain	Res	Type
3	C	141	PRO
3	F	141	PRO

5.3.2 Protein sidechains ⓘ

In the following table, the Percentiles column shows the percent sidechain outliers of the chain as a percentile score with respect to all PDB entries followed by that with respect to all EM entries.

The Analysed column shows the number of residues for which the sidechain conformation was analysed, and the total number of residues.

Mol	Chain	Analysed	Rotameric	Outliers	Percentiles	
1	A	108/145 (74%)	102 (94%)	6 (6%)	23	23
1	D	108/145 (74%)	102 (94%)	6 (6%)	23	23
2	B	339/350 (97%)	338 (100%)	1 (0%)	93	93
2	E	339/350 (97%)	338 (100%)	1 (0%)	93	93
3	C	206/210 (98%)	200 (97%)	6 (3%)	45	45
3	F	206/210 (98%)	200 (97%)	6 (3%)	45	45
All	All	1306/1410 (93%)	1280 (98%)	26 (2%)	61	58

All (26) residues with a non-rotameric sidechain are listed below:

Mol	Chain	Res	Type
1	A	11	ILE
1	A	13	ILE
1	A	44	LYS
1	A	52	ILE
1	A	59	VAL
1	A	143	THR

Continued on next page...

PRELIMINARY VALIDATION REPORT

Continued from previous page...

Mol	Chain	Res	Type
2	B	108	THR
3	C	93	LYS
3	C	97	LYS
3	C	106	PHE
3	C	127	ASP
3	C	160	LEU
3	C	164	LYS
1	D	11	ILE
1	D	13	ILE
1	D	44	LYS
1	D	52	ILE
1	D	59	VAL
1	D	143	THR
2	E	108	THR
3	F	93	LYS
3	F	97	LYS
3	F	106	PHE
3	F	127	ASP
3	F	160	LEU
3	F	164	LYS

Some sidechains can be flipped to improve hydrogen bonding and reduce clashes. All (15) such sidechains are listed below:

Mol	Chain	Res	Type
1	A	113	HIS
2	B	51	GLN
2	B	147	GLN
2	B	321	ASN
2	B	373	ASN
3	C	161	ASN
3	C	198	ASN
3	C	232	ASN
1	D	113	HIS
2	E	51	GLN
2	E	321	ASN
2	E	373	ASN
3	F	161	ASN
3	F	198	ASN
3	F	232	ASN

PRELIMINARY VALIDATION REPORT

5.3.3 RNA ⓘ

There are no RNA molecules in this entry.

5.4 Non-standard residues in protein, DNA, RNA chains ⓘ

There are no non-standard protein/DNA/RNA residues in this entry.

5.5 Carbohydrates ⓘ

There are no carbohydrates in this entry.

5.6 Ligand geometry ⓘ

Of 17 ligands modelled in this entry, 4 could not be matched to an existing wwPDB Chemical Component Dictionary definition at this stage - leaving 13 for Mogul analysis.

In the following table, the Counts columns list the number of bonds (or angles) for which Mogul statistics could be retrieved, the number of bonds (or angles) that are observed in the model and the number of bonds (or angles) that are defined in the Chemical Component Dictionary. The Link column lists molecule types, if any, to which the group is linked. The Z score for a bond length (or angle) is the number of standard deviations the observed value is removed from the expected value. A bond length (or angle) with $|Z| > 2$ is considered an outlier worth inspection. RMSZ is the root-mean-square of all Z scores of the bond lengths (or angles).

Mol	Type	Chain	Res	Link	Bond lengths			Bond angles		
					Counts	RMSZ	$\#\|Z\| > 2$	Counts	RMSZ	$\#\|Z\| > 2$
4	FES	A	501	1	0,4,4	0.00	-	-		
5	HEM	B	501	2	27,50,50	2.03	4 (14%)	17,82,82	1.55	4 (23%)
5	HEM	B	502	2	27,50,50	1.82	4 (14%)	17,82,82	1.72	5 (29%)
6	AMY	B	503	-	39,39,39	1.86	8 (20%)	36,53,53	2.11	9 (25%)
8	PGV	B	506	2	50,50,50	0.88	3 (6%)	53,56,56	0.86	1 (1%)
8	PGV	B	507	-	50,50,50	0.88	4 (8%)	53,56,56	0.86	2 (3%)
9	HEC	C	501	3	26,50,50	2.44	4 (15%)	18,82,82	2.21	6 (33%)
4	FES	D	501	1	0,4,4	0.00	-	-		
5	HEM	E	501	2	27,50,50	2.03	4 (14%)	17,82,82	1.54	4 (23%)
5	HEM	E	502	2	27,50,50	1.83	4 (14%)	17,82,82	1.73	5 (29%)
6	AMY	E	503	-	39,39,39	1.86	8 (20%)	36,53,53	2.03	8 (22%)
8	PGV	E	506	2	50,50,50	0.88	3 (6%)	53,56,56	0.86	1 (1%)
9	HEC	F	501	3	26,50,50	2.45	4 (15%)	18,82,82	2.22	6 (33%)

PRELIMINARY VALIDATION REPORT

In the following table, the Chirals column lists the number of chiral outliers, the number of chiral centers analysed, the number of these observed in the model and the number defined in the Chemical Component Dictionary. Similar counts are reported in the Torsion and Rings columns. '-' means no outliers of that kind were identified.

Mol	Type	Chain	Res	Link	Chirals	Torsions	Rings
4	FES	A	501	1	-	-	0/1/1/1
5	HEM	B	501	2	-	1/6/54/54	-
5	HEM	B	502	2	-	2/6/54/54	-
6	AMY	B	503	-	-	17/37/52/52	0/1/2/2
8	PGV	B	506	2	-	31/55/55/55	-
8	PGV	B	507	-	-	22/55/55/55	-
9	HEC	C	501	3	-	5/6/54/54	-
4	FES	D	501	1	-	-	0/1/1/1
5	HEM	E	501	2	-	1/6/54/54	-
5	HEM	E	502	2	-	2/6/54/54	-
6	AMY	E	503	-	-	19/37/52/52	0/1/2/2
8	PGV	E	506	2	-	31/55/55/55	-
9	HEC	F	501	3	-	5/6/54/54	-

All (50) bond length outliers are listed below:

Mol	Chain	Res	Type	Atoms	Z	Observed(Å)	Ideal(Å)
9	F	501	HEC	C3B-C2B	-7.74	1.32	1.40
9	C	501	HEC	C3B-C2B	-7.64	1.32	1.40
5	B	501	HEM	C3C-C2C	-6.42	1.31	1.40
5	E	501	HEM	C3C-C2C	-6.39	1.31	1.40
9	F	501	HEC	C3C-C2C	-6.05	1.34	1.40
9	C	501	HEC	C3C-C2C	-6.02	1.34	1.40
5	E	501	HEM	C3B-C2B	-5.48	1.32	1.40
5	B	501	HEM	C3B-C2B	-5.39	1.32	1.40
6	B	503	AMY	C2-C1	5.28	1.48	1.40
6	E	503	AMY	C2-C1	5.27	1.48	1.40
9	C	501	HEC	C3D-C2D	5.00	1.52	1.37
9	F	501	HEC	C3D-C2D	5.00	1.52	1.37
5	E	502	HEM	C3B-C2B	-4.84	1.33	1.40
5	B	502	HEM	C3B-C2B	-4.79	1.33	1.40
5	E	502	HEM	C3C-C2C	-4.70	1.33	1.40
5	B	502	HEM	C3C-C2C	-4.68	1.33	1.40
6	B	503	AMY	O4-C20	4.67	1.45	1.34
6	E	503	AMY	O5-C14	4.67	1.45	1.34
6	B	503	AMY	O5-C14	4.65	1.45	1.34
6	E	503	AMY	O4-C20	4.57	1.45	1.34

Continued on next page...

PRELIMINARY VALIDATION REPORT

Continued from previous page...

Mol	Chain	Res	Type	Atoms	Z	Observed(Å)	Ideal(Å)
6	E	503	AMY	C6-C1	4.03	1.48	1.41
6	E	503	AMY	O8-C21	4.02	1.45	1.34
6	B	503	AMY	O8-C21	4.02	1.45	1.34
6	B	503	AMY	C2-N1	-3.99	1.35	1.41
6	B	503	AMY	C6-C1	3.98	1.47	1.41
6	E	503	AMY	C2-N1	-3.97	1.35	1.41
5	E	502	HEM	C3B-CAB	3.22	1.54	1.47
5	B	502	HEM	C3B-CAB	3.18	1.54	1.47
5	E	502	HEM	C3C-CAC	3.17	1.54	1.47
5	B	502	HEM	C3C-CAC	3.16	1.54	1.47
5	B	501	HEM	C3B-CAB	3.06	1.54	1.47
5	E	501	HEM	C3B-CAB	3.03	1.54	1.47
5	B	501	HEM	C3C-CAC	2.86	1.53	1.47
5	E	501	HEM	C3C-CAC	2.83	1.53	1.47
8	E	506	PGV	O03-C19	2.65	1.41	1.33
8	B	506	PGV	O03-C19	2.64	1.41	1.33
8	B	507	PGV	O01-C02	-2.57	1.40	1.46
9	F	501	HEC	CAD-C3D	2.38	1.55	1.52
9	C	501	HEC	CAD-C3D	2.34	1.55	1.52
6	E	503	AMY	O4-C11	-2.30	1.42	1.46
8	B	507	PGV	O03-C19	2.30	1.40	1.33
8	E	506	PGV	O01-C1	2.27	1.40	1.34
8	B	506	PGV	O01-C1	2.27	1.40	1.34
6	B	503	AMY	O5-C10	-2.25	1.42	1.46
8	B	507	PGV	O03-C01	-2.24	1.40	1.45
8	B	506	PGV	O01-C02	-2.21	1.41	1.46
8	E	506	PGV	O01-C02	-2.18	1.41	1.46
6	E	503	AMY	O5-C10	-2.15	1.43	1.46
8	B	507	PGV	O01-C1	2.05	1.40	1.34
6	B	503	AMY	O4-C11	-2.03	1.43	1.46

All (51) bond angle outliers are listed below:

Mol	Chain	Res	Type	Atoms	Z	Observed(^{o})	Ideal(^{o})
6	B	503	AMY	O4-C20-C9	6.61	120.68	110.28
6	E	503	AMY	O4-C20-C9	6.28	120.16	110.28
6	B	503	AMY	O8-C21-C22	4.96	120.67	111.45
9	C	501	HEC	C1D-C2D-C3D	-4.58	103.81	107.00
9	F	501	HEC	C1D-C2D-C3D	-4.57	103.82	107.00
6	E	503	AMY	O8-C21-C22	4.42	119.67	111.45
6	B	503	AMY	O4-C20-O7	-4.30	118.66	124.08
8	B	506	PGV	O01-C1-C2	4.26	120.80	111.51

Continued on next page...

PRELIMINARY VALIDATION REPORT

Continued from previous page...

Mol	Chain	Res	Type	Atoms	Z	Observed(^{o})	Ideal(^{o})
8	E	506	PGV	O01-C1-C2	4.25	120.79	111.51
6	E	503	AMY	O4-C20-O7	-4.15	118.85	124.08
8	B	507	PGV	O01-C1-C2	3.96	120.15	111.51
6	E	503	AMY	O5-C14-O6	-3.74	119.37	124.08
9	C	501	HEC	CAD-CBD-CGD	-3.74	106.28	112.66
9	F	501	HEC	CMC-C2C-C1C	-3.73	122.74	128.46
9	F	501	HEC	CAD-CBD-CGD	-3.71	106.33	112.66
6	B	503	AMY	O2-C8-N1	-3.70	121.00	125.79
9	C	501	HEC	CMC-C2C-C1C	-3.70	122.78	128.46
6	E	503	AMY	O2-C8-N1	-3.65	121.06	125.79
6	B	503	AMY	O5-C14-O6	-3.48	119.70	124.08
5	E	502	HEM	CAD-CBD-CGD	-3.34	106.95	112.66
5	B	502	HEM	CAD-CBD-CGD	-3.34	106.95	112.66
9	F	501	HEC	CMC-C2C-C3C	3.02	129.38	125.82
9	C	501	HEC	CMC-C2C-C3C	2.96	129.31	125.82
5	B	501	HEM	CAD-CBD-CGD	-2.91	107.69	112.66
5	E	501	HEM	CAD-CBD-CGD	-2.89	107.72	112.66
6	E	503	AMY	C2-N1-C8	-2.88	121.08	126.74
5	E	501	HEM	CAA-CBA-CGA	-2.84	107.81	112.66
6	B	503	AMY	C2-N1-C8	-2.82	121.20	126.74
5	B	501	HEM	CAA-CBA-CGA	-2.81	107.86	112.66
5	B	502	HEM	CMC-C2C-C3C	2.74	129.95	124.80
5	E	502	HEM	CMC-C2C-C3C	2.74	129.94	124.80
5	E	501	HEM	CBD-CAD-C3D	-2.58	107.55	112.47
5	B	501	HEM	CBD-CAD-C3D	-2.57	107.56	112.47
8	B	507	PGV	O03-C19-C20	2.52	120.03	111.93
5	E	502	HEM	C4A-C3A-C2A	2.51	108.74	107.00
6	E	503	AMY	C9-N2-C7	2.49	126.11	121.49
6	B	503	AMY	C9-N2-C7	2.46	126.05	121.49
5	E	502	HEM	CAA-CBA-CGA	-2.41	108.55	112.66
5	B	502	HEM	C4A-C3A-C2A	2.41	108.67	107.00
5	B	502	HEM	CAA-CBA-CGA	-2.40	108.56	112.66
5	B	502	HEM	CMB-C2B-C3B	2.39	129.28	124.80
9	C	501	HEC	CAA-CBA-CGA	-2.38	108.59	112.66
5	E	502	HEM	CMB-C2B-C3B	2.37	129.25	124.80
9	F	501	HEC	CAA-CBA-CGA	-2.37	108.61	112.66
6	B	503	AMY	C12-O8-C21	-2.30	113.76	117.58
5	E	501	HEM	CMD-C2D-C1D	-2.09	125.26	128.46
9	F	501	HEC	CMB-C2B-C1B	-2.06	125.30	128.46
6	B	503	AMY	O9-C21-C22	-2.06	120.10	124.71
5	B	501	HEM	CMD-C2D-C1D	-2.06	125.30	128.46
9	C	501	HEC	CMB-C2B-C1B	-2.04	125.32	128.46

Continued on next page...

PRELIMINARY VALIDATION REPORT

Continued from previous page...

Mol	Chain	Res	Type	Atoms	Z	Observed(^{o})	Ideal(^{o})
6	E	503	AMY	O9-C21-C22	-2.01	120.21	124.71

There are no chirality outliers.

All (136) torsion outliers are listed below:

Mol	Chain	Res	Type	Atoms
5	B	502	HEM	C1A-C2A-CAA-CBA
5	B	502	HEM	C3A-C2A-CAA-CBA
5	E	502	HEM	C1A-C2A-CAA-CBA
5	E	502	HEM	C3A-C2A-CAA-CBA
8	B	507	PGV	O12-C04-C05-C06
8	B	507	PGV	C04-C05-C06-O06
6	B	503	AMY	C13-C14-O5-C10
6	B	503	AMY	O6-C14-O5-C10
6	B	503	AMY	O2-C8-N1-C2
6	B	503	AMY	C11-C12-C13-C15
6	B	503	AMY	O8-C12-C13-C14
6	B	503	AMY	C11-C12-O8-C21
6	B	503	AMY	C13-C12-O8-C21
6	B	503	AMY	C12-C13-C14-O6
5	E	501	HEM	C3D-CAD-CBD-CGD
9	F	501	HEC	C1A-C2A-CAA-CBA
9	F	501	HEC	C3A-C2A-CAA-CBA
9	F	501	HEC	C4D-C3D-CAD-CBD
8	E	506	PGV	C03-O11-P-O14
8	E	506	PGV	O02-C1-O01-C02
9	C	501	HEC	C1A-C2A-CAA-CBA
9	C	501	HEC	C3A-C2A-CAA-CBA
9	C	501	HEC	C4D-C3D-CAD-CBD
8	B	506	PGV	C03-O11-P-O14
8	B	506	PGV	O02-C1-O01-C02
5	B	501	HEM	C3D-CAD-CBD-CGD
6	E	503	AMY	C13-C14-O5-C10
6	E	503	AMY	O6-C14-O5-C10
6	E	503	AMY	O2-C8-N1-C2
6	E	503	AMY	O4-C11-C12-C13
6	E	503	AMY	O4-C11-C12-O8
6	E	503	AMY	C27-C11-C12-O8
6	E	503	AMY	O8-C12-C13-C14
6	E	503	AMY	C13-C12-O8-C21
6	E	503	AMY	C12-C13-C14-O6
8	E	506	PGV	C2-C1-O01-C02

Continued on next page...

PRELIMINARY VALIDATION REPORT

Continued from previous page...

Mol	Chain	Res	Type	Atoms
8	B	506	PGV	C2-C1-O01-C02
8	E	506	PGV	C20-C19-O03-C01
8	B	506	PGV	C20-C19-O03-C01
6	E	503	AMY	C6-C7-N2-C9
6	B	503	AMY	C6-C7-N2-C9
8	E	506	PGV	O04-C19-O03-C01
8	B	506	PGV	O04-C19-O03-C01
8	B	507	PGV	O12-C04-C05-O05
8	E	506	PGV	C19-C20-C21-C22
8	B	506	PGV	C19-C20-C21-C22
8	B	507	PGV	C19-C20-C21-C22
8	E	506	PGV	C03-O11-P-O12
8	B	506	PGV	C03-O11-P-O12
6	E	503	AMY	O3-C7-N2-C9
6	B	503	AMY	O3-C7-N2-C9
8	E	506	PGV	C29-C30-C31-C32
8	E	506	PGV	C1-C2-C3-C4
8	B	506	PGV	C1-C2-C3-C4
8	B	506	PGV	C29-C30-C31-C32
8	B	507	PGV	C6-C7-C8-C9
8	B	507	PGV	C27-C28-C29-C30
8	E	506	PGV	C23-C24-C25-C26
8	B	506	PGV	C23-C24-C25-C26
8	E	506	PGV	C21-C22-C23-C24
8	B	506	PGV	C21-C22-C23-C24
8	E	506	PGV	C04-C05-C06-O06
8	B	506	PGV	C04-C05-C06-O06
8	B	507	PGV	C2-C3-C4-C5
8	B	507	PGV	C4-C5-C6-C7
6	E	503	AMY	C22-C21-O8-C12
6	E	503	AMY	O9-C21-O8-C12
8	E	506	PGV	C30-C31-C32-C33
8	B	506	PGV	C30-C31-C32-C33
8	E	506	PGV	C2-C3-C4-C5
8	B	506	PGV	C2-C3-C4-C5
8	E	506	PGV	C3-C4-C5-C6
8	B	506	PGV	C3-C4-C5-C6
8	B	507	PGV	C23-C24-C25-C26
8	B	507	PGV	C20-C21-C22-C23
6	B	503	AMY	O8-C21-C22-C23
8	B	507	PGV	C28-C29-C30-C31
8	B	507	PGV	O05-C05-C06-O06

Continued on next page...

PRELIMINARY VALIDATION REPORT

Continued from previous page...

Mol	Chain	Res	Type	Atoms
8	E	506	PGV	O05-C05-C06-O06
8	B	506	PGV	O05-C05-C06-O06
8	E	506	PGV	C01-C02-O01-C1
8	B	506	PGV	C01-C02-O01-C1
8	E	506	PGV	C22-C23-C24-C25
8	B	506	PGV	C22-C23-C24-C25
8	E	506	PGV	C5-C6-C7-C8
8	B	506	PGV	C5-C6-C7-C8
8	E	506	PGV	O01-C02-C03-O11
8	B	506	PGV	O01-C02-C03-O11
6	B	503	AMY	C15-C16-C17-C18
8	B	507	PGV	C24-C25-C26-C27
8	B	506	PGV	C31-C32-C33-C34
8	E	506	PGV	C31-C32-C33-C34
6	B	503	AMY	O9-C21-C22-C23
8	B	507	PGV	C21-C22-C23-C24
8	E	506	PGV	C20-C21-C22-C23
8	B	506	PGV	C20-C21-C22-C23
8	E	506	PGV	C03-O11-P-O13
8	B	506	PGV	C03-O11-P-O13
9	F	501	HEC	C3D-CAD-CBD-CGD
9	C	501	HEC	C3D-CAD-CBD-CGD
8	E	506	PGV	C15-C16-C17-C18
8	B	506	PGV	C15-C16-C17-C18
8	B	507	PGV	O01-C02-C03-O11
6	B	503	AMY	O8-C12-C13-C15
6	B	503	AMY	O7-C20-O4-C11
9	F	501	HEC	C2D-C3D-CAD-CBD
9	C	501	HEC	C2D-C3D-CAD-CBD
6	E	503	AMY	O7-C20-O4-C11
8	E	506	PGV	C6-C7-C8-C9
8	B	507	PGV	C25-C26-C27-C28
8	B	506	PGV	C6-C7-C8-C9
8	E	506	PGV	C11-C12-C13-C14
8	B	506	PGV	C11-C12-C13-C14
8	B	507	PGV	C01-C02-C03-O11
8	B	507	PGV	C9-C10-C11-C12
8	B	506	PGV	C14-C15-C16-C17
8	E	506	PGV	C14-C15-C16-C17
6	E	503	AMY	C15-C16-C17-C18
6	B	503	AMY	C13-C15-C16-C17
6	B	503	AMY	C16-C17-C18-C19

Continued on next page...

PRELIMINARY VALIDATION REPORT

Continued from previous page...

Mol	Chain	Res	Type	Atoms
6	E	503	AMY	C16-C17-C18-C19
8	B	507	PGV	C15-C16-C17-C18
8	E	506	PGV	O12-C04-C05-C06
8	B	506	PGV	O12-C04-C05-C06
6	E	503	AMY	C13-C15-C16-C17
8	B	507	PGV	C11-C10-C9-C8
8	E	506	PGV	O12-C04-C05-O05
8	B	506	PGV	O12-C04-C05-O05
8	E	506	PGV	C9-C10-C11-C12
8	B	506	PGV	C9-C10-C11-C12
6	E	503	AMY	O9-C21-C22-C23
8	E	506	PGV	C12-C13-C14-C15
8	B	506	PGV	C12-C13-C14-C15
8	B	507	PGV	O03-C01-C02-O01
8	B	507	PGV	C11-C12-C13-C14
6	E	503	AMY	O8-C21-C22-C23

There are no ring outliers.

12 monomers are involved in 86 short contacts:

Mol	Chain	Res	Type	Clashes	Symm-Clashes
4	A	501	FES	1	0
5	B	501	HEM	3	0
5	B	502	HEM	5	0
6	B	503	AMY	25	0
8	B	506	PGV	4	0
8	B	507	PGV	2	0
9	C	501	HEC	6	0
5	E	501	HEM	3	0
5	E	502	HEM	5	0
6	E	503	AMY	20	0
8	E	506	PGV	4	0
9	F	501	HEC	8	0

The following is a two-dimensional graphical depiction of Mogul quality analysis of bond lengths, bond angles, torsion angles, and ring geometry for all instances of the Ligand of Interest. In addition, ligands with molecular weight > 250 and outliers as shown on the validation Tables will also be included. For torsion angles, if less then 5% of the Mogul distribution of torsion angles is within 10 degrees of the torsion angle in question, then that torsion angle is considered an outlier. Any bond that is central to one or more torsion angles identified as an outlier by Mogul will be highlighted in the graph. For rings, the root-mean-square deviation (RMSD) between the ring in question and similar rings identified by Mogul is calculated over all ring torsion angles. If the

PRELIMINARY VALIDATION REPORT

average RMSD is greater than 60 degrees and the minimal RMSD between the ring in question and any Mogul-identified rings is also greater than 60 degrees, then that ring is considered an outlier. The outliers are highlighted in purple. The color gray indicates Mogul did not find sufficient equivalents in the CSD to analyse the geometry.

Ligand HEM B 501

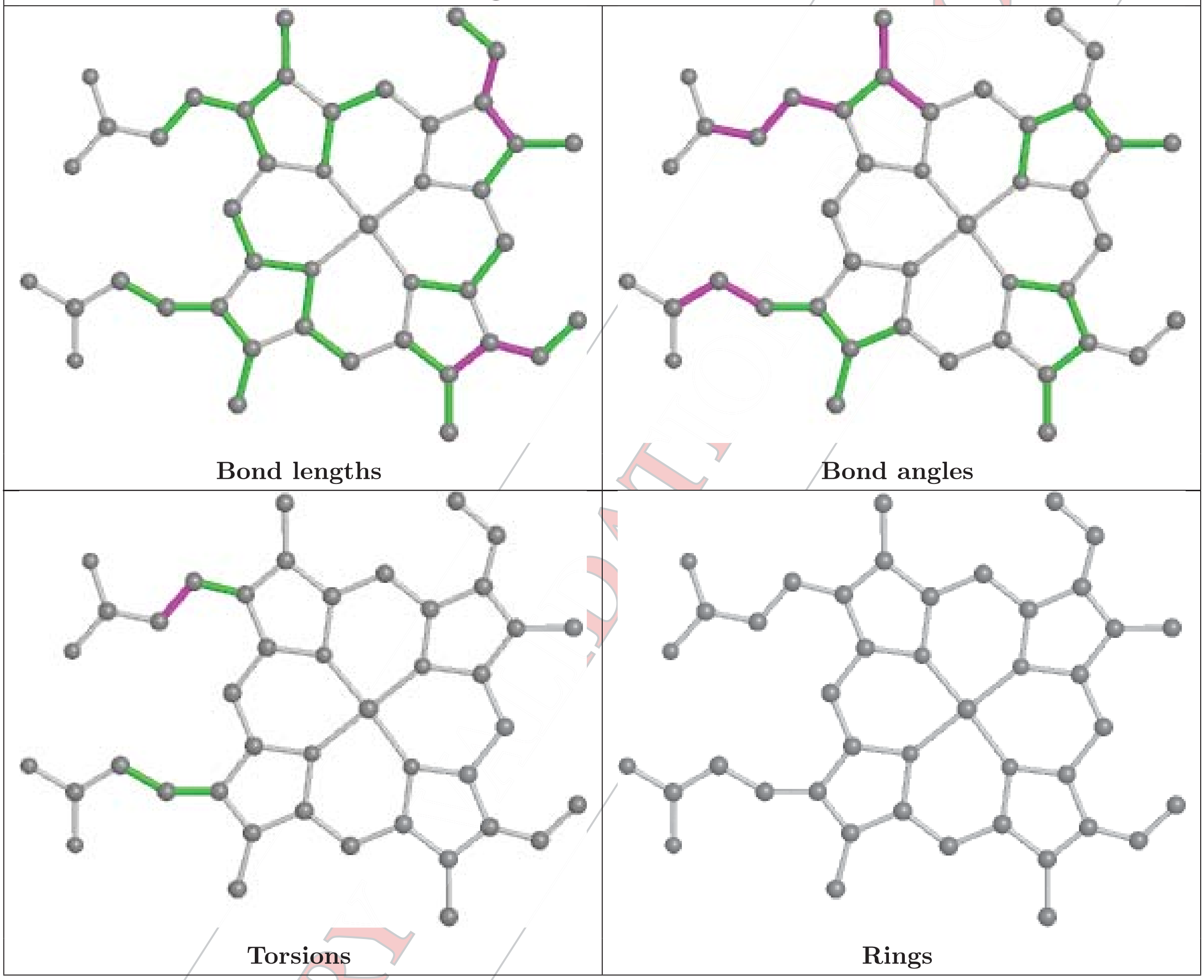

PRELIMINARY REPORT

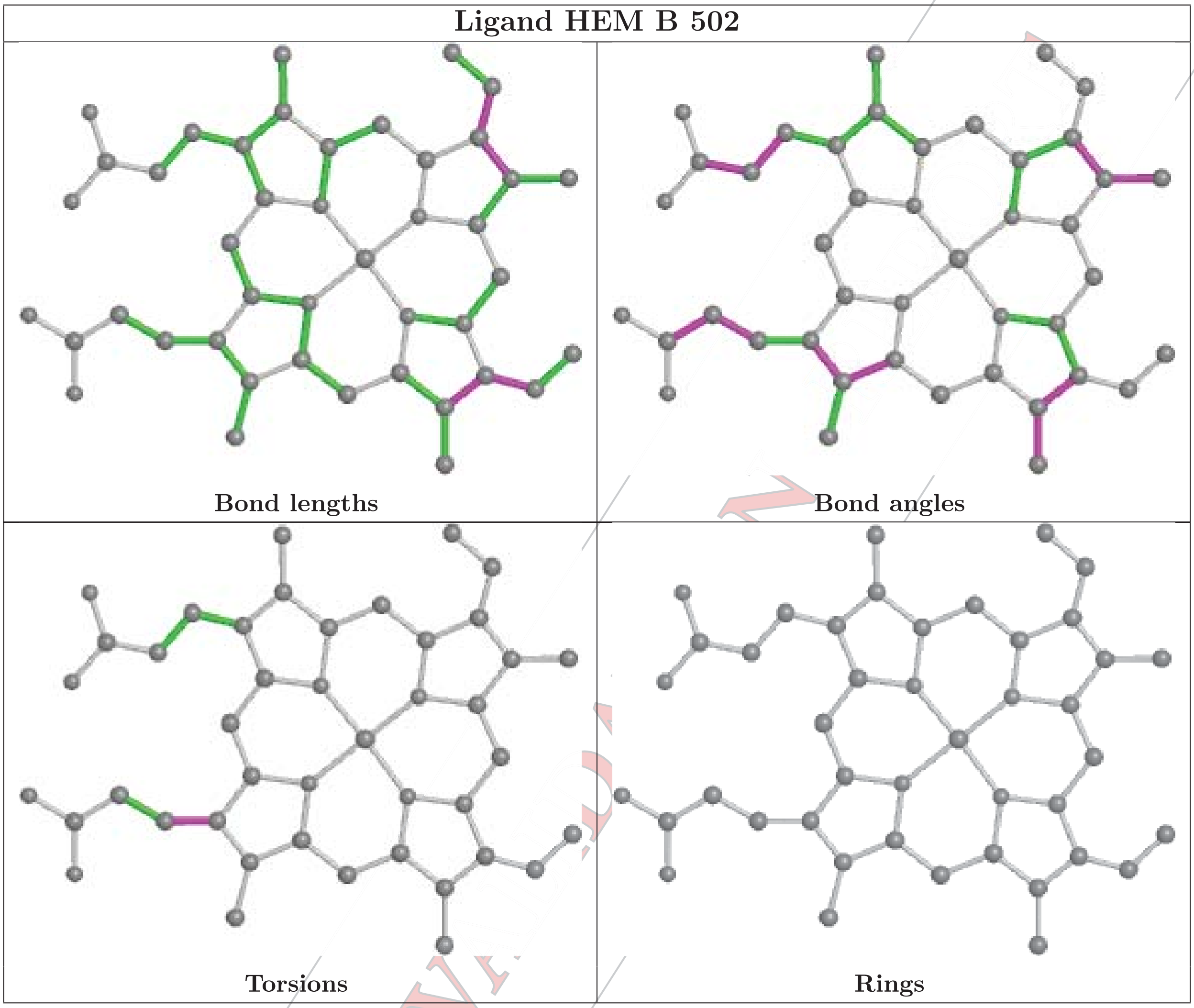

PRELIMINARY VALIDATION

Ligand AMY B 503

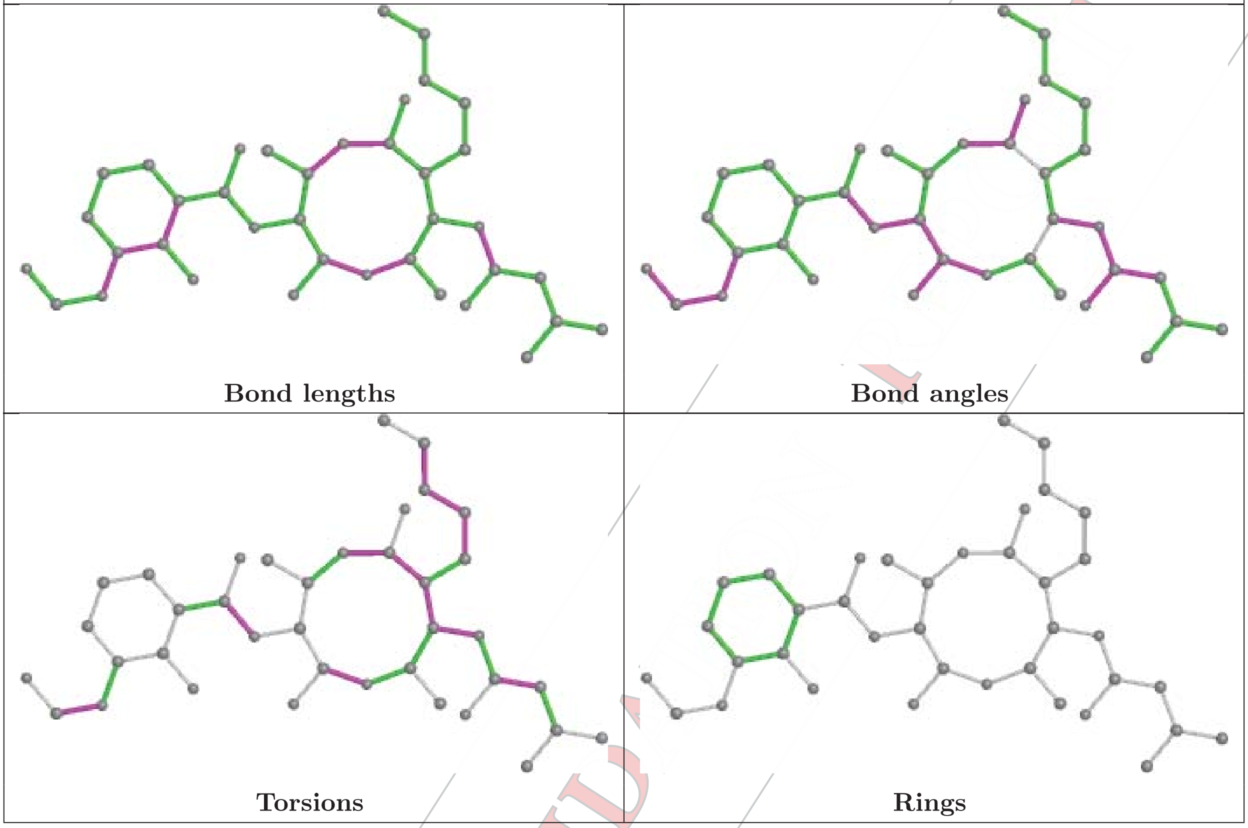

Ligand PGV B 506

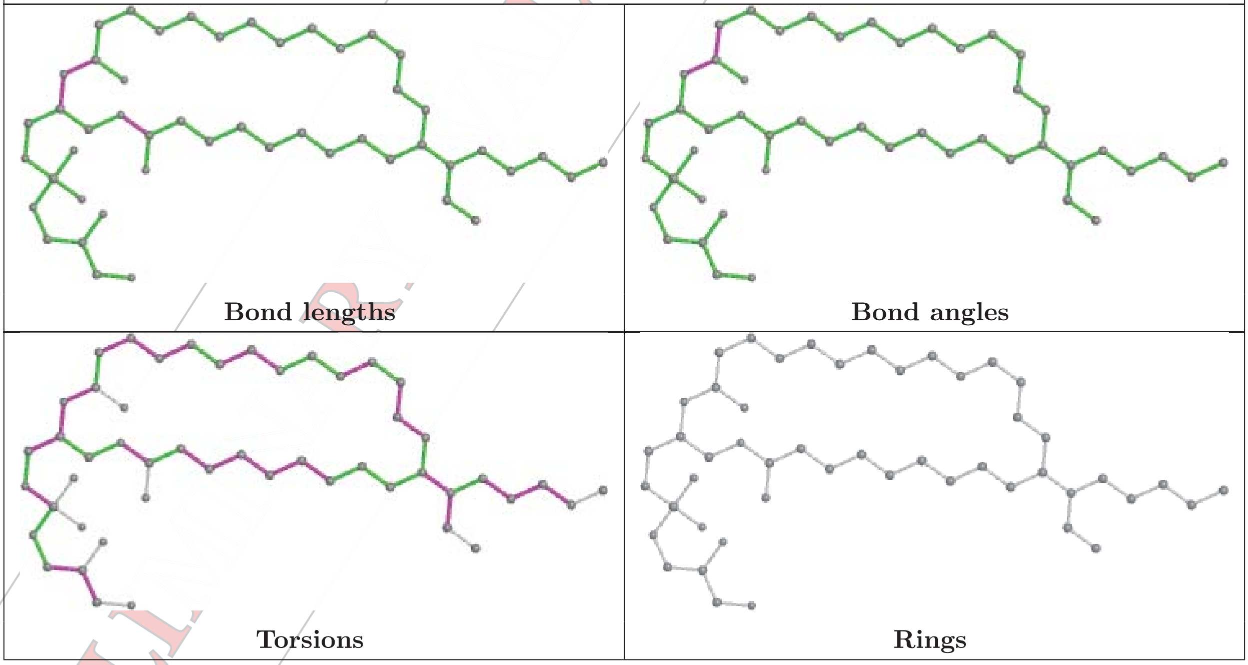

Ligand PGV B 507

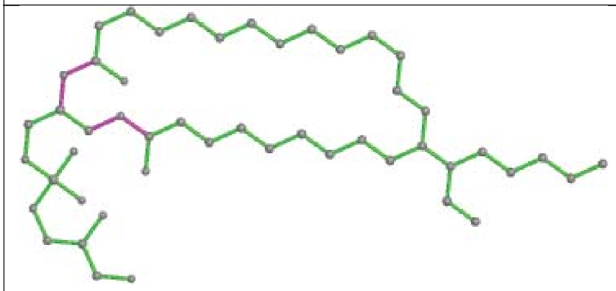

Bond lengths

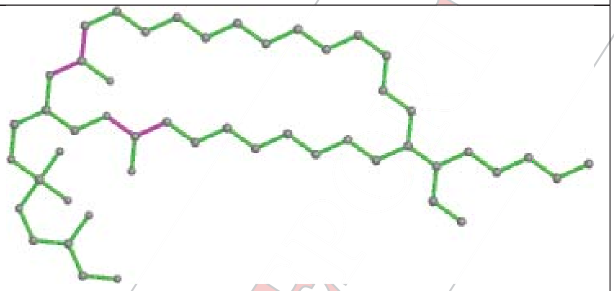

Bond angles

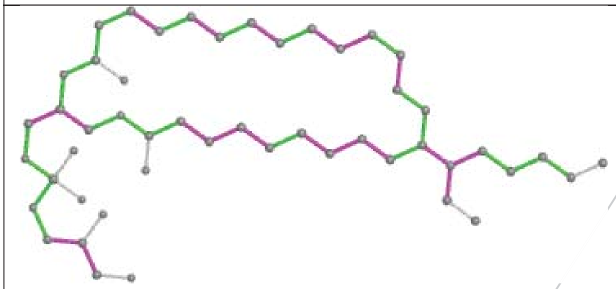

Torsions

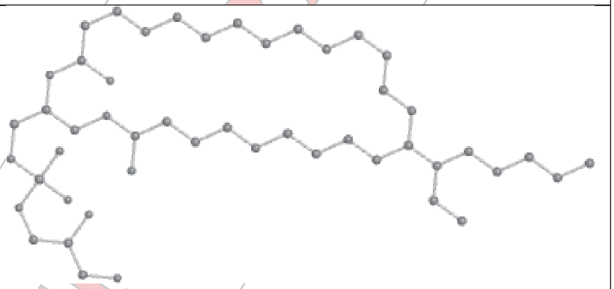

Rings

PRELIMINARY VALIDATION

Ligand HEC C 501

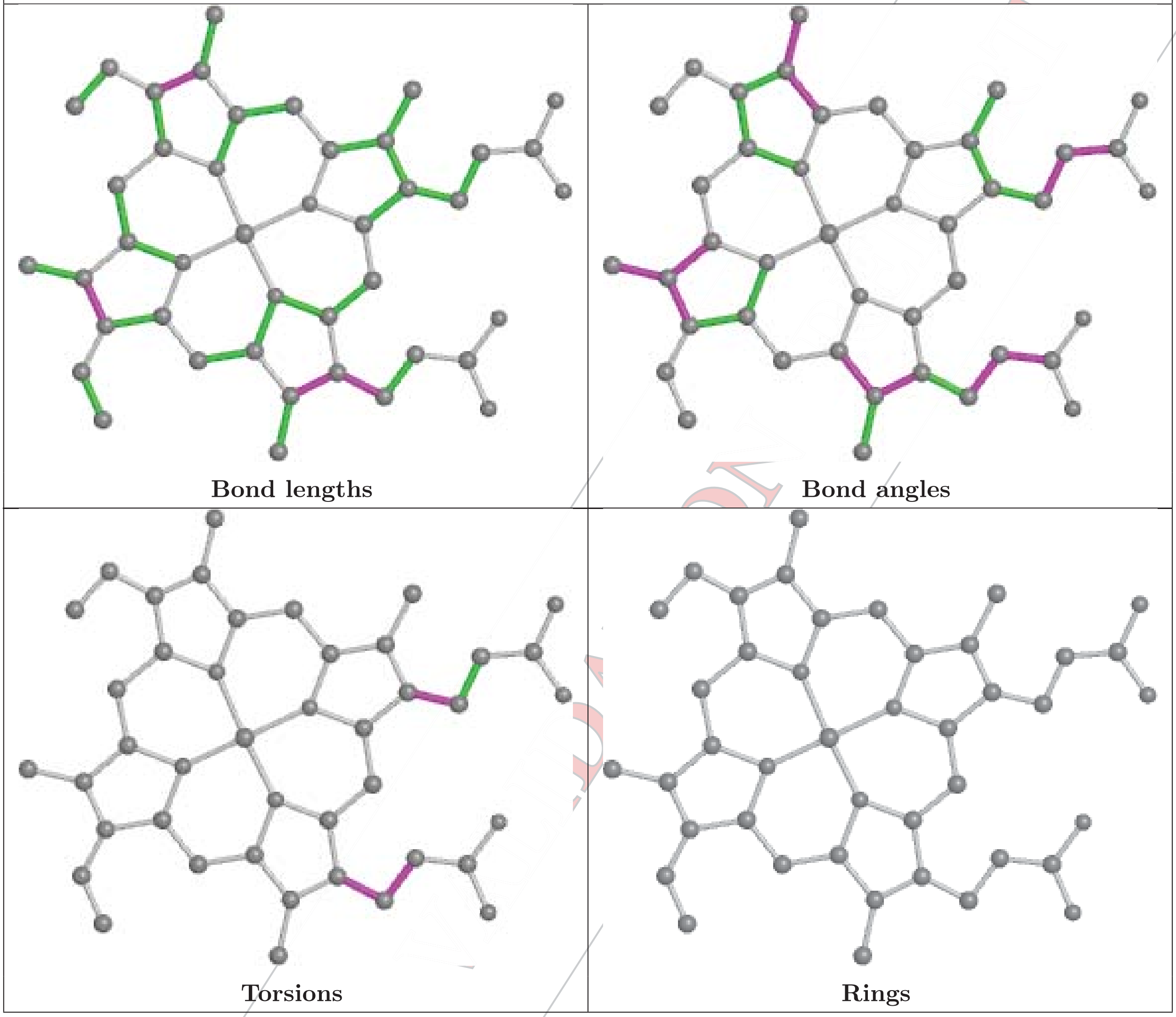

PRELIMINARY

Ligand HEM E 501

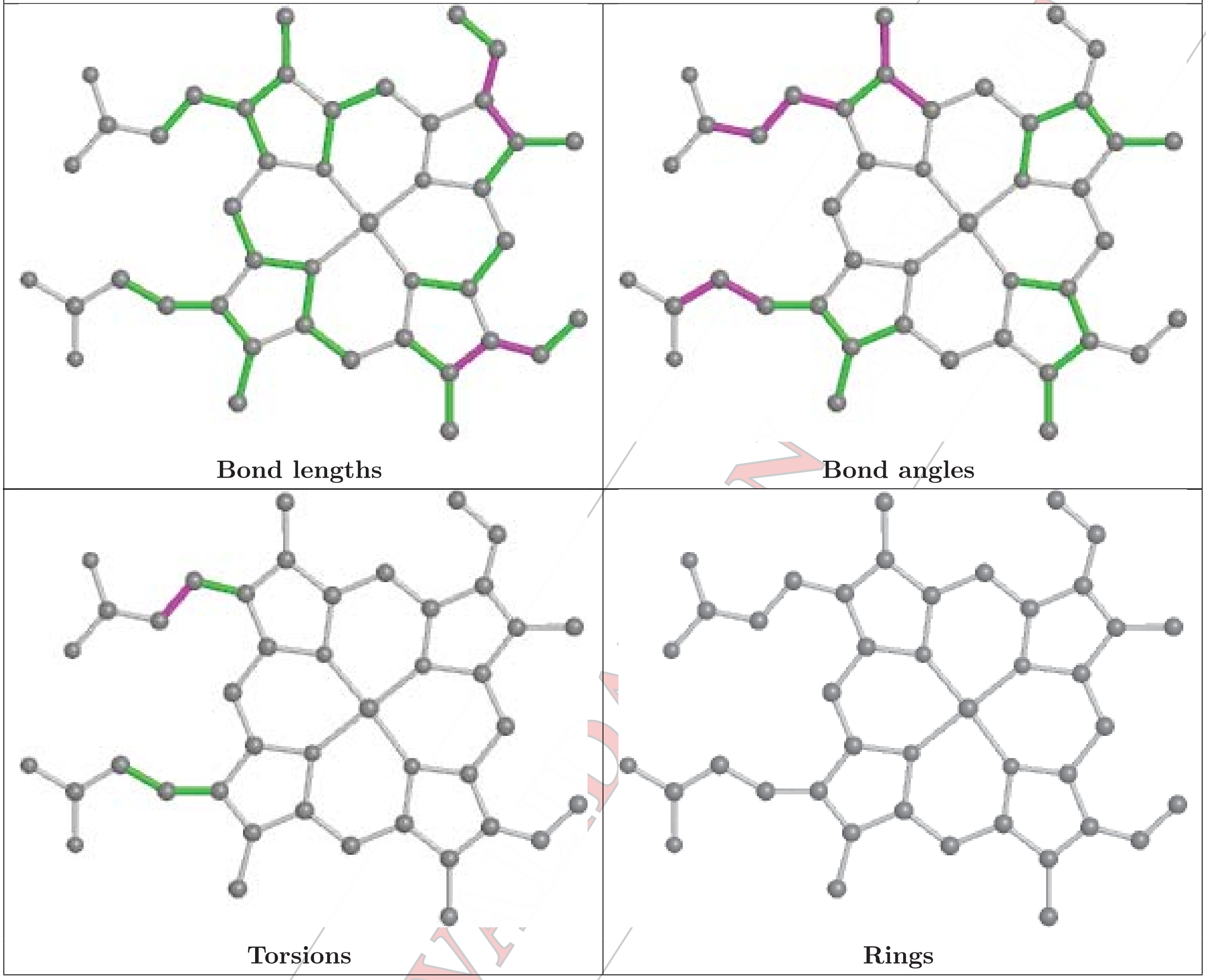

PRELIMINARY VALIDATION

Ligand HEM E 502

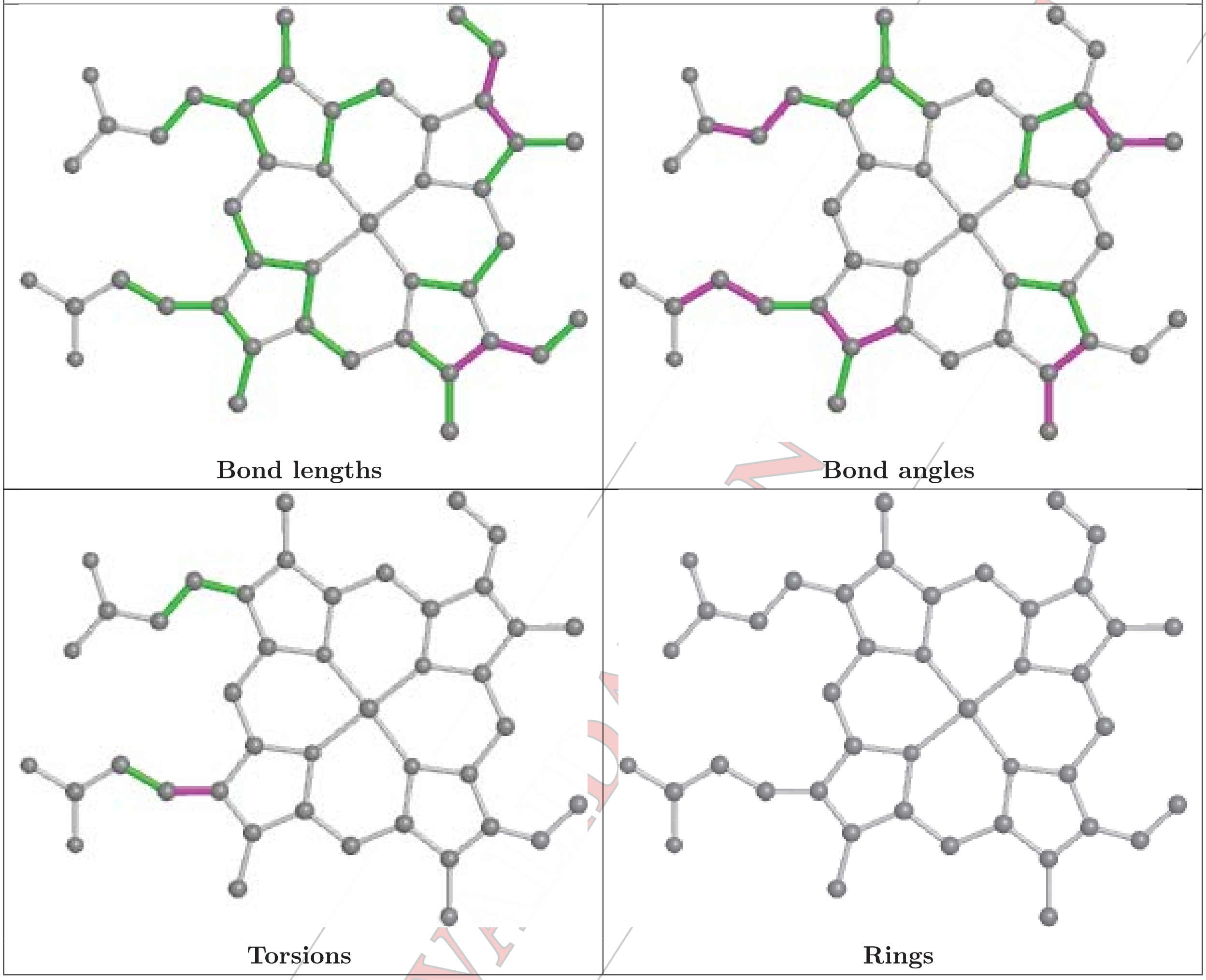

PRELIMINARY VALIDATION

Ligand AMY E 503

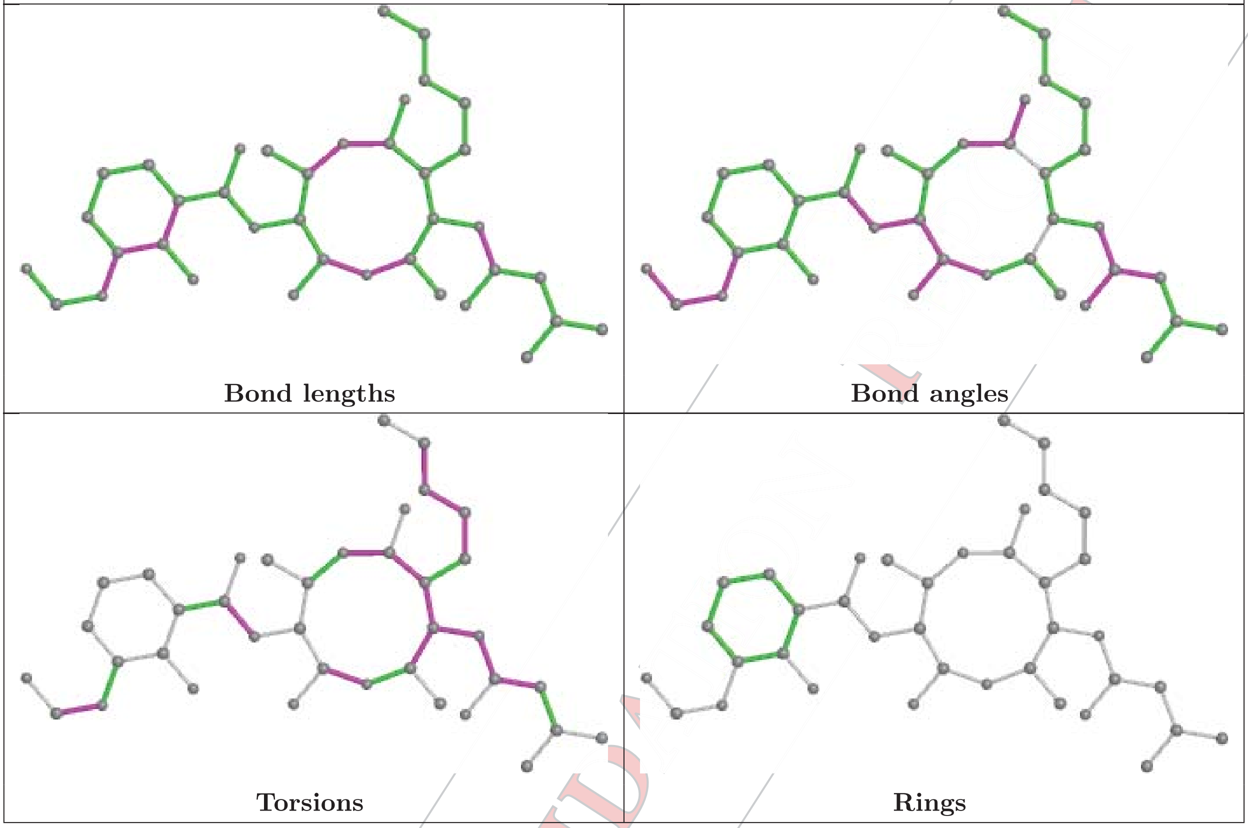

Ligand PGV E 506

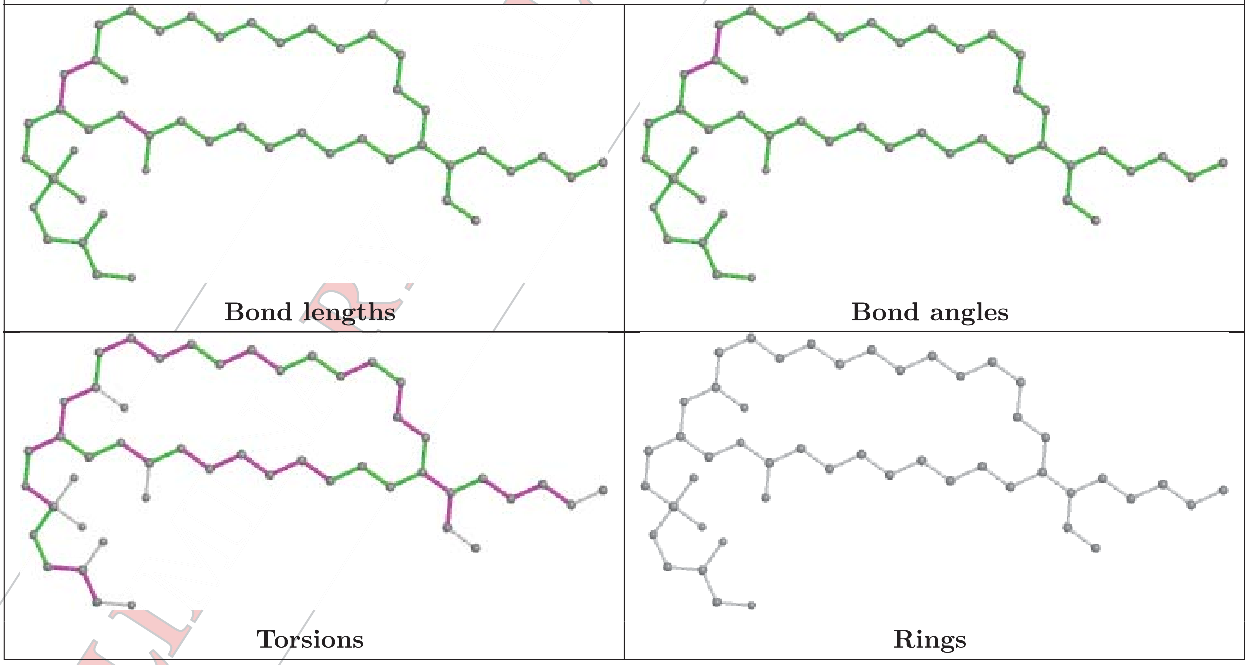

Ligand HEC F 501

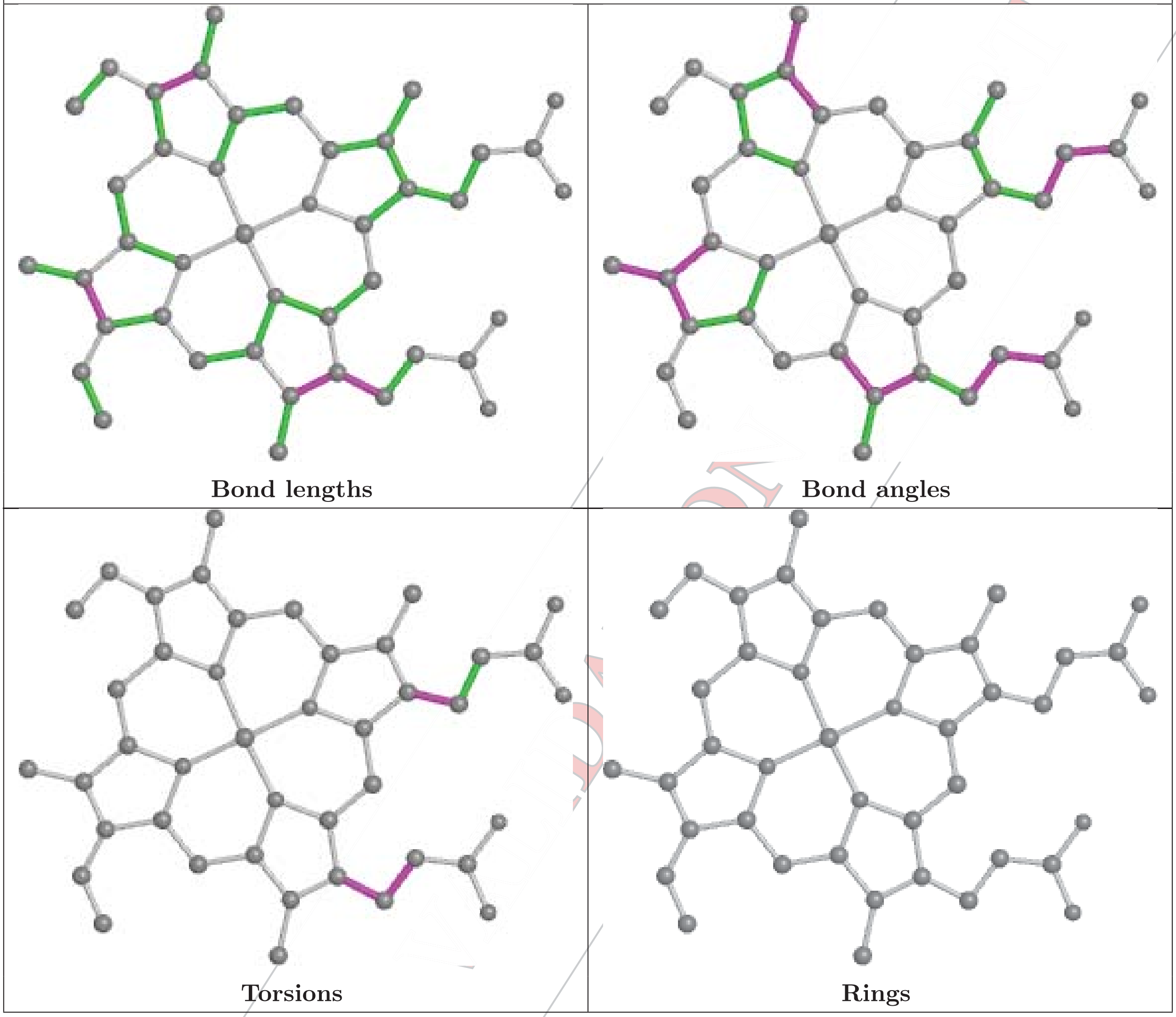

Ligand NQA B 504

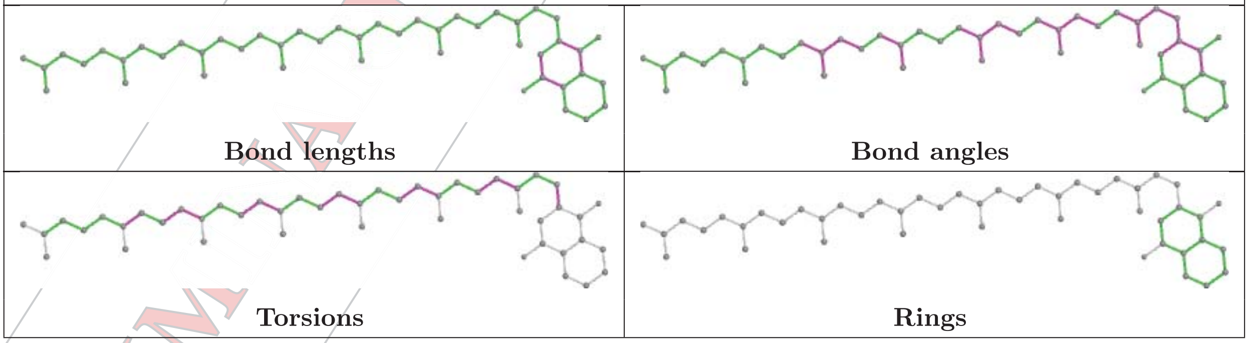

Ligand NQA E 505

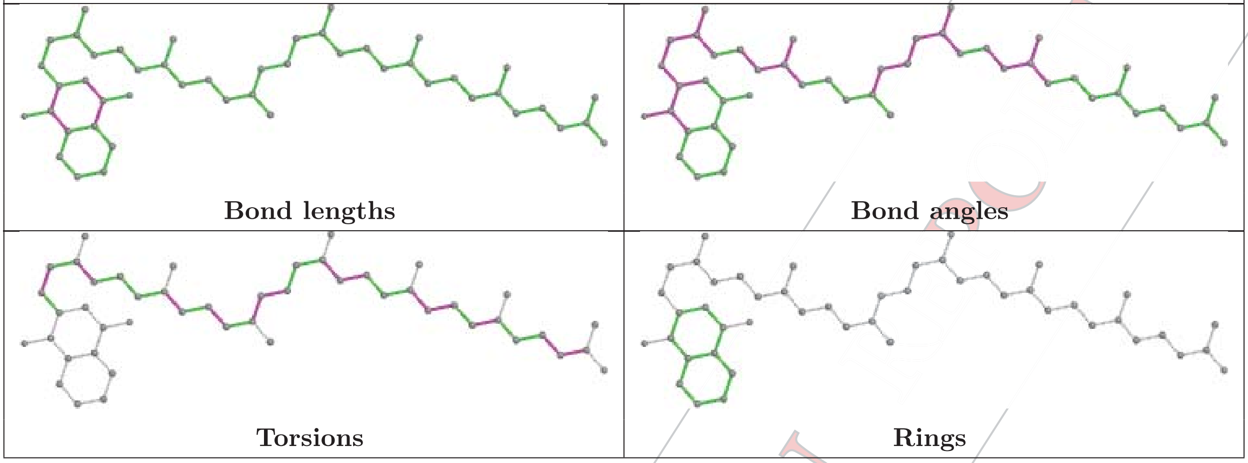

Ligand NQA B 505

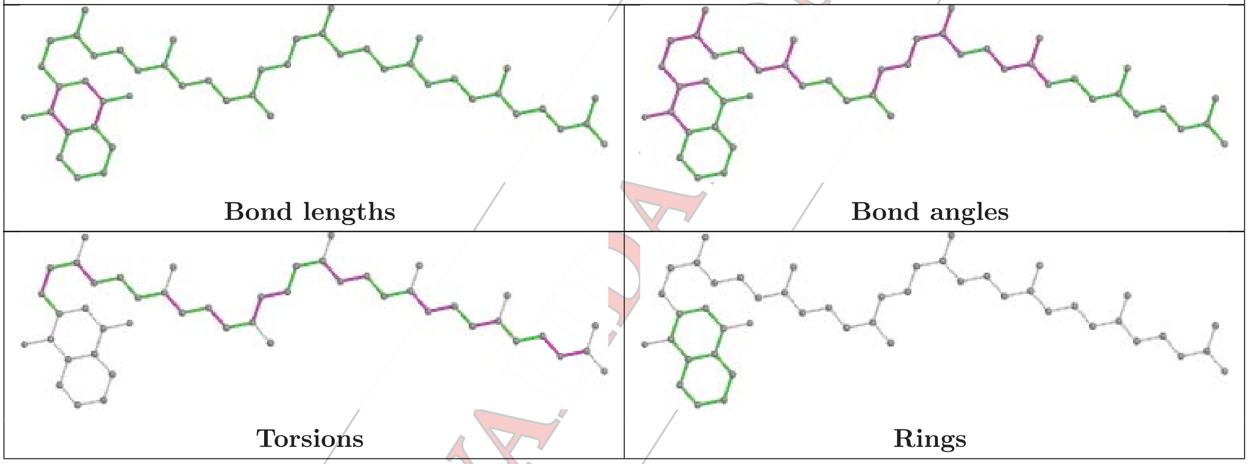

Ligand NQA E 504

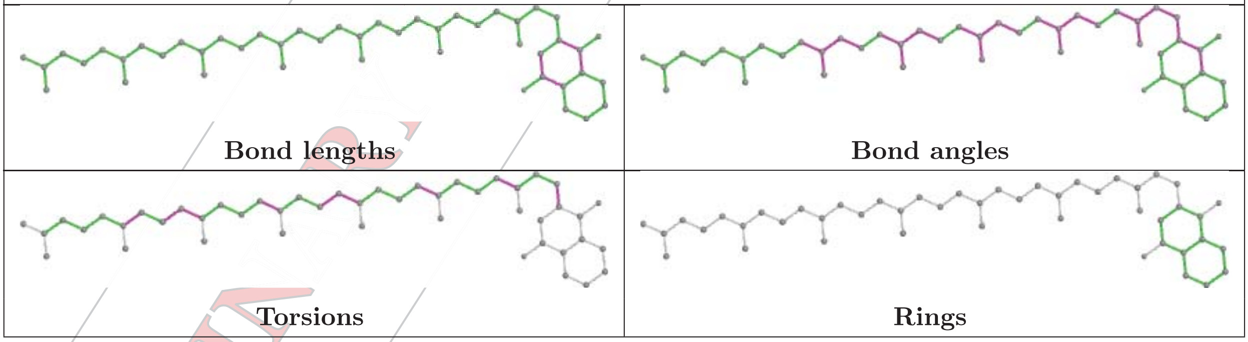

5.7 Other polymers ⓘ

There are no such residues in this entry.

5.8 Polymer linkage issues ⓘ

There are no chain breaks in this entry.

PRELIMINARY VALIDATION
